# Assembly and Specific Recognition of K29- and K33-Linked Polyubiquitin

**DOI:** 10.1016/j.molcel.2015.01.042

**Published:** 2015-04-02

**Authors:** Martin A. Michel, Paul R. Elliott, Kirby N. Swatek, Michal Simicek, Jonathan N. Pruneda, Jane L. Wagstaff, Stefan M.V. Freund, David Komander

**Affiliations:** 1 Medical Research Council Laboratory of Molecular Biology, Francis Crick Avenue, Cambridge CB2 0QH, UK

## Abstract

Protein ubiquitination regulates many cellular processes via attachment of structurally and functionally distinct ubiquitin (Ub) chains. Several atypical chain types have remained poorly characterized because the enzymes mediating their assembly and receptors with specific binding properties have been elusive. We found that the human HECT E3 ligases UBE3C and AREL1 assemble K48/K29- and K11/K33-linked Ub chains, respectively, and can be used in combination with DUBs to generate K29- and K33-linked chains for biochemical and structural analyses. Solution studies indicate that both chains adopt open and dynamic conformations. We further show that the N-terminal Npl4-like zinc finger (NZF1) domain of the K29/K33-specific deubiquitinase TRABID specifically binds K29/K33-linked diUb, and a crystal structure of this complex explains TRABID specificity and suggests a model for chain binding by TRABID. Our work uncovers linkage-specific components in the Ub system for atypical K29- and K33-linked Ub chains, providing tools to further understand these unstudied posttranslational modifications.

## Highlights

• The HECT E3 ligases UBE3C and AREL1 assemble K29- and K33-linked polyubiquitin, respectively
• K29- and K33-linked chains adopt open conformations in solution
• The N-terminal NZF1 domain of TRABID specifically recognizes K29/K33-diubiquitin
• A structure of a K33 filament bound to NZF1 domains explains TRABID specificity

## Graphical Abstract

**Figure:**
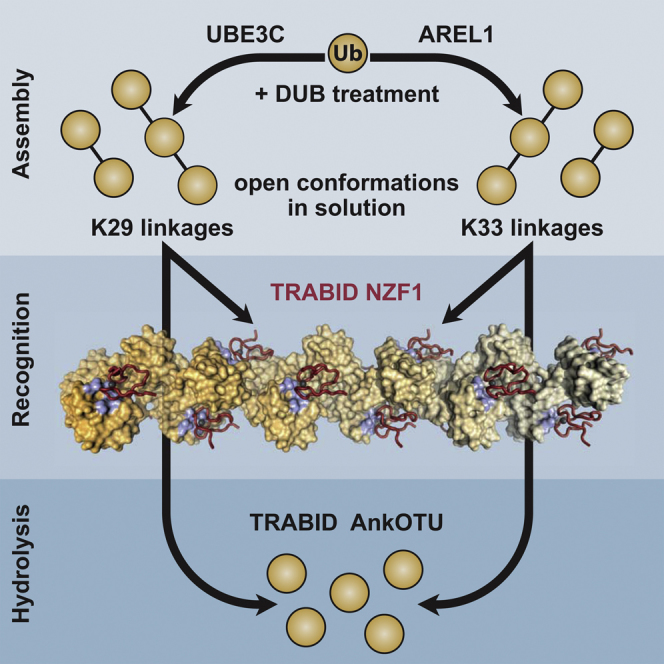

## Teaser

Michel et al. reveal that UBE3C and AREL1 assemble K29- and K33-linked polyubiquitin, respectively, on substrates and as unanchored chains. They further identify a K29/K33-specific ubiquitin binding domain in TRABID and structurally characterize how TRABID recognizes K29/K33 filaments.

## Introduction

Protein ubiquitination is an important posttranslational modification that affects virtually every cellular process. Its best-studied function is the degradation of proteins by the proteasome (Hershko and Ciechanover, 1998). However, ubiquitination also regulates alternative degradation pathways, such as ER-associated degradation, autophagy, and mitophagy (Christianson and Ye, 2014; Shaid et al., 2013). In addition, ubiquitination has many non-degradative roles in protein kinase signaling, DNA damage response, intracellular trafficking, transcription, and translation (Komander and Rape, 2012).

During ubiquitination, the 76-amino acid protein ubiquitin (Ub) is attached via its C terminus to, most commonly, Lys residues on substrate proteins. The versatility of Ub in regulating cellular processes arises from its ability to form a wide variety of polyUb chains (Komander and Rape, 2012). Ub has seven internal Lys residues and an N-terminal amino group, all of which can be ubiquitinated, leading to the formation of polyUb chains. Proteomic analyses have revealed that all Ub chain linkages exist simultaneously in cells (Kim et al., 2011; Wagner et al., 2011; Xu et al., 2009). Chains can be homotypic, in which only one linkage type is present, but also heterotypic, in which multiple linkages form mixed and branched structures (Komander and Rape, 2012). Importantly, different linkage types have distinct cellular roles. K48-linked Ub chains are proteasomal degradation signals, whereas K63-linked Ub chains are mainly non-degradative. For the remaining six “atypical” linkage types, cellular roles are less clear. K11-linked chains are important in cell-cycle regulation, where they seem to constitute an alternative proteasomal degradation signal (Wickliffe et al., 2011) but also have other roles (Bremm and Komander, 2011), whereas M1-linked chains have independent non-degradative roles, in particular during NFκB activation and apoptosis (Iwai et al., 2014). For the remaining four chain types, linked via K6, K27, K29, and K33, very little is known, and proteins generating and recognizing these chains in eukaryotic cells are still elusive (Kulathu and Komander, 2012).

The process of ubiquitination is facilitated by an enzymatic cascade in which an E1 Ub-activating enzyme transfers Ub onto the active-site Cys of an E2 Ub-conjugating enzyme (Schulman and Harper, 2009; Ye and Rape, 2009). The E2 enzyme can directly discharge its Ub onto substrates, usually with the help of a RING or U-box E3 ligase (Deshaies and Joazeiro, 2009). Alternatively, a subset of E2 enzymes can perform a transthiolation reaction by transferring Ub onto the active-site Cys of a HECT or RBR E3 ligase. When charged with Ub, HECT and RBR E3 ligases modify substrates in an E2-independent manner (Berndsen and Wolberger, 2014). Importantly, a number of E2 enzymes as well as several HECT and RBR E3 ligases are known to assemble polyUb in a linkage-specific fashion (Kulathu and Komander, 2012; Mattiroli and Sixma, 2014). Based on this knowledge, we have previously described enzymatic assembly systems for K11- and K6-linked chains using a modified E2- and a HECT-like E3 ligase, respectively (Bremm et al., 2010; Hospenthal et al., 2013).

When polyUb chains are generated, they are recognized by Ub binding domains (UBDs), some of which bind polyUb chains in a linkage-specific manner (Husnjak and Dikic, 2012). Linkage-specific UBDs for M1-linked chains have been described (e.g., Sato et al., 2011) but are unknown for the remaining atypical chain types. Deubiquitinases (DUBs) hydrolyze Ub chains, in some cases with linkage preference (Clague et al., 2013; Komander et al., 2009). Characterization of the ovarian tumor (OTU) DUB family has revealed enzymes to hydrolyze atypical chain types specifically (Keusekotten et al., 2013; Licchesi et al., 2012; Mevissen et al., 2013; Ritorto et al., 2014; Rivkin et al., 2013).

In our search for assembly systems of atypical Ub chain types, we confirmed an earlier report showing that UBE3C primarily assembles K29- and K48-linked chains (You and Pickart, 2001) and further discovered that the HECT E3 ligase apoptosis-resistant E3 ubiquitin protein ligase 1 (AREL1), also known as KIAA0317 (Kim et al., 2013), assembles atypical K11- and K33-linked chains in autoubiquitination reactions and predominantly K33-linkages in free chains and on reported substrates. Treatment of assembly reactions with linkage-specific DUBs enabled purification of K29- and K33-linked polyUb in quantities suitable for biophysical and structural studies. This enabled the structural characterization of the polymers and of the K29/K33 linkage-specific OTU family DUB TRABID (Licchesi et al., 2012). DiUb of both linkage types adopt open conformations in solution, similar to K63-linked polyUb. We found that the TRABID N terminus, which contains three Npl4-type zinc finger (NZF) UBDs, specifically binds K29- and K33-linked diUb, and specificity can be attributed to the first NZF domain (NZF1). A crystal structure of NZF1 bound to K33-linked diUb reveals an intriguing filamentous structure for K33 polymers in which NZF1 binds each Ub-Ub interface. A similar binding mode is observed for K29-linkages in solution studies, together suggesting a model for TRABID interaction with atypical chains. Inactive TRABID localizes to Ub-rich puncta in cells, and this is attenuated when the K29/K33-specific binding mode is disrupted by point mutations. Our work unlocks K29- and K33-linked Ub chains for biochemical studies and provides a launching point for future discoveries related to these atypical Ub signals.

## Results

### HECT E3 Ligases Assemble Atypical Ub Chains

The HECT family of E3 ligases contains 28 members, many of which have important cellular functions (Rotin and Kumar, 2009). Much research has focused on the first discovered member, E6AP (Scheffner et al., 1993), and on the NEDD4 family, which comprises Rsp5 in yeast and nine enzymes in humans (Rotin and Kumar, 2009). Interestingly, although E6AP assembles K48-linked chains, the NEDD4 family specifically assembles K63 linkages in vitro. Elegant biochemical and structural work has identified many features important for linkage specificity (Kamadurai et al., 2009, 2013; Kim and Huibregtse, 2009; Maspero et al., 2013). Because of their ability to dictate linkage specificity and many hints in the literature (Tran et al., 2013; You and Pickart, 2001), we characterized human HECT E3 ligases to investigate their ability to assemble atypical chains.

**Figure 1:**
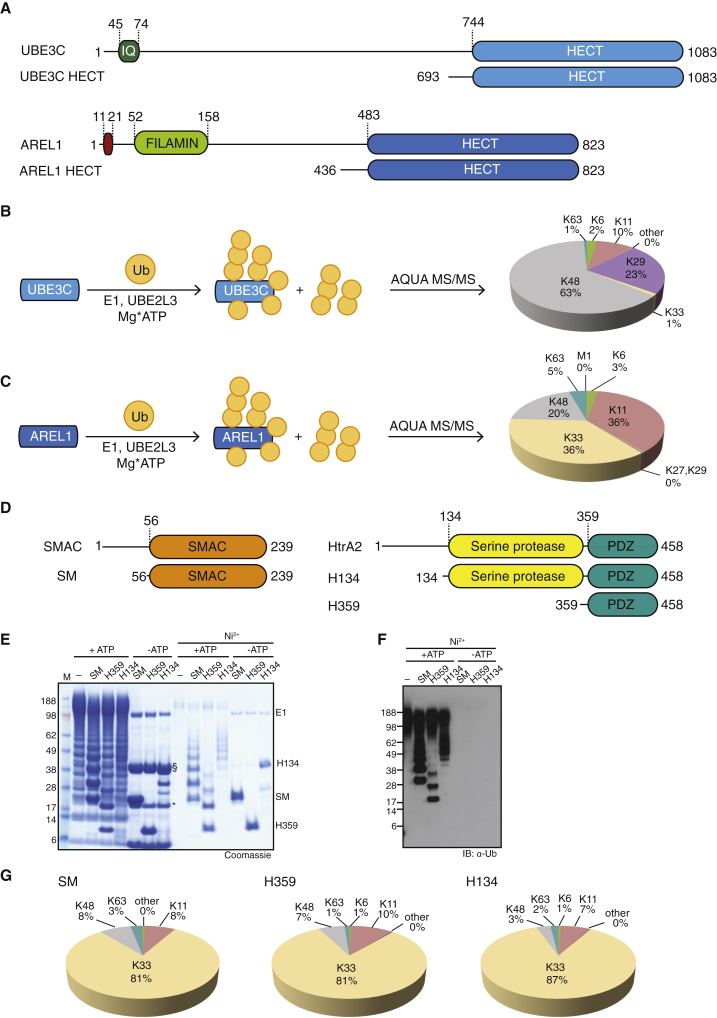
Role of HECT E3 Ligases in Assembling Atypical Ub Chains (A) Domain structures of UBE3C and AREL1 (KIAA0317) (top) and constructs used in this study (bottom). (B) Schematic of an assembly reaction with UBE3C, UBE2L3 (UbcH7), E1, and WT Ub (left). The linkage composition in the reaction mixture was analyzed by AQUA-based MS/MS (right). (C) Reaction as in (B) with AREL1, UBE2L3, E1, and WT Ub. (D) Domain structures of the pro-apoptotic proteins SMAC and HtrA2 (top) and the expressed constructs used in this work (bottom). (E) AREL1 is able to assemble chains onto SMAC and HtrA2 in an in vitro ubiquitination reaction that depends on ATP. Ubiquitinated, His6-tagged substrates are enriched following Ni^2+^ affinity binding. SM, SMAC (56–239); H359, HtrA2 (359–458); H134, HtrA2 (134–458); §, AREL1; ^∗^, UBE2L3. (F) Western blot against Ub of the Ni^2+^-enriched reaction from (E). (G) AQUA MS/MS profiles of the ubiquitinated substrates purified from (E).

One way to assess which linkage types are assembled is to utilize a panel of Ub mutants in which each Lys is mutated to Arg either inclusively (K0) or with the exception of one position (Kx-only) (Figure S1A). This analysis reproduced K63 specificity of NEDD4L (amino acids [aa] 576–955) (Figure S1B) and indicated a broader specificity of the unstudied HECT E3 ligase AREL1 (aa 436–823, Figure 1A; Figure S1C), which seemed to assemble K33 linkages efficiently.

Using Ub mutants for chain assembly has multiple caveats. To understand which linkage types are assembled in E3 ligase reactions with wild-type (WT) Ub, we used absolute quantification (AQUA)-based mass spectrometry (Kirkpatrick et al., 2006). For this, tryptic digests of chain assembly reactions are spiked with isotope-labeled GlyGly-modified standard peptides derived from each potential linkage site, allowing absolute quantification of all chain types (Kirkpatrick et al., 2006). NEDD4L assembled K63 chains almost exclusively (96%) (Figure S1D), whereas UBE3C assembled K48 (63%), K29 (23%), and K11 linkages (10%) (Figure 1B), as reported previously (Maspero et al., 2013; You and Pickart, 2001). Interestingly, AREL1 assembled 36% of K33, 36% of K11, 20% of K48, and small amounts of K63 and K6 linkages (Figure 1C). The high abundance of K11 linkages in AREL1 reactions contrasts with the finding from K11-only Ub that was incorporated poorly into chains (Figure S1C), suggesting that mutated Lys residues are crucial for assembly of this linkage type by AREL1. Abrogating K11 linkage production by AREL1 using Ub K11R led to 71% of K33 and 24% of K48 linkages (Figure S1E). The fact that K48 linkages stayed relatively constant indicated that this chain type is assembled as a constant byproduct of AREL1.

A recent characterization of AREL1 function (Kim et al., 2013) has suggested that the pro-apoptotic proteins SMAC, HtrA2, and ARTS are among its substrates and further indicated that they interact via the AREL1 HECT domain (rather than an auxiliary N-terminal domain). We expressed fragments of SMAC and HtrA2 (Figure 1D) and used these proteins as in vitro AREL1 substrates. AREL1 ubiquitinated all proteins efficiently (Figures 1E and 1F). Strikingly, AQUA analysis of modified substrates showed that AREL1 had assembled >80% of K33 linkages in the polyUb chains on all three substrates (Figure 1G).

### Generating K29- and K33-Linked PolyUb

**Figure 2:**
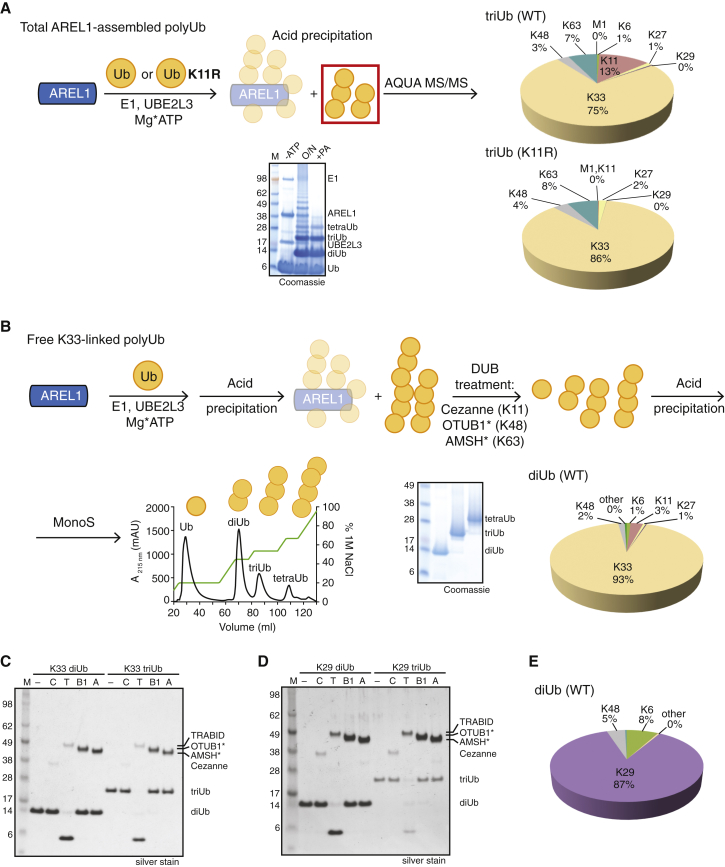
Purification of Unanchored K29/K33 PolyUb Chains (A) Schematic of the assembly of K33-linked Ub chains using either WT or K11R Ub (top). AQUA profiles of triUb using either WT (top right) or K11R Ub (bottom right; K6 linkage was excluded from the quantitative analysis because of the K11R substitution). Bottom: corresponding SDS-PAGE gel for assembly of free chains. −ATP, initial reaction without ATP addition; O/N, overnight incubation of the assembly reaction; +PA, perchloric acid treatment of the assembly reaction. (B) Schematic representation of the purification of K33-linked polyUb chains. Following the assembly reaction, perchloric acid treatment removes the ubiquitinated and unmodified forms of E1, E2, and E3. Linkage-selective DUBs are then used to remove undesired Ub linkages. An additional perchloric acid step is required to inactivate the DUBs prior to cation exchange chromatography (bottom), which resolves the homotypic chains based on linkage length. Bottom center: SDS-PAGE of purified K33-linked di-, tri-, and tetraUb. Bottom right: AQUA MS/MS of purified K33-linked diUb. (C) Deubiquitinase assay of purified K33-linked di- and triUb. –, no DUB; C, 200 nM Cezanne (K11-specific); T, 350 nM TRABID (K29/K33-specific); B1, 1 μM OTUB1^∗^ (K48-specific); A, 1 μM AMSH^∗^ (K63-specific). (D) K29-linked polyUb chains can be purified analogous to the schematic shown in (B). Purified K29-linked di- and triUb were treated with DUBs as in (C). (E) AQUA mass spectrometry profile of purified K29 diUb.

AREL1 and UBE3C also assembled free Ub chains. Precipitation of enzymes by perchloric acid in an assembly reaction enriched free polyUb chains of varying lengths (see gel in Figure 2A). AREL1 assembled WT Ub into di- and triUb with (for triUb) 75% of K33-linkages and only 13% of K11-linked and 7% of K63-linked chains (Figure 2A). Using K11R Ub, we generated up to 86% of K33 linkages in triUb (Figure 2A). To generate pure K29 and K33 chains from WT Ub, we acid-precipitated the reaction and treated the free chains with a panel of linkage-specific DUBs consisting of K11-specific Cezanne (Mevissen et al., 2013) as well as enhanced versions of K48-specific OTUB1^∗^ (an UBE2D2-OTUB1 fusion) and K63-specific AMSH^∗^ (a STAM2-AMSH fusion) (Figure 2B; Figures S2A and S2B; Supplemental Experimental Procedures). The resulting K29- and K33-linked polyUb chains were purified by cation exchange and were 87% and 93% pure, respectively; uncleavable by Cezanne, OTUB1^∗^, or AMSH^∗^; but hydrolyzed efficiently by the K29/K33-specific DUB TRABID (Licchesi et al., 2012) (Figures 2C–2E; Figures S2C and S2D; also see below).

### K29- and K33-Linked diUbs Adopt Open Conformations in Solution

With new linkage types at hand, we set out to understand their structural features. We crystallized K33-linked di- and triUbs (Figure S3). The K33-linked diUb crystallized in space group *I*4, not observed previously for Ub, and a structure to 1.85 Å resolution revealed eight molecules forming four identical Ub dimers with clear electron density for the K33 linkages (Figures S3A and S3B; Table 1). In this structure, K33-linked diUb is compact, and distal and proximal Ub moieties interact symmetrically via their Ile36 hydrophobic patches (Figure S3A). The symmetric interface did not provide a model for the conformation of a longer K33 chain.

A second crystal structure for K33-linked triUb was obtained at 1.68 Å in space group *P*2_1_2_1_2_1_ with similar unit cell dimensions as the Ub reference structure (1ubq; Vijay-Kumar et al., 1987), and also contains only one Ub molecule per asymmetric unit (Table 1). Examination of adjacent asymmetric units only allowed one possibility for K33 chain formation (Figure S3C), although the C termini and isopeptide linkages were poorly ordered and not built in the model (Figure S3D). The Ub moieties in K33 chains were related by translational symmetry and, in contrast to the compact diUb structure, adopted an open conformation in which Ub moieties do not interact with each other except by two polar side chain contacts (Figure S3C). The distinct conformations of di- and triUb could be due to differences in crystallization conditions but highlight the underlying problem that crystallization may present an incomplete picture of the dynamic states of free polyUb in solution.

We therefore turned to nuclear magnetic resonance (NMR), which is better suited to analyze dynamic Ub chains. When Ub polymers adopt open conformations in solution, the spectra resemble free monoUb, showing a small number of perturbations surrounding the isopeptide linkage. The best example for this is K63-linked Ub (Varadan et al., 2004) which does not form a defined interface in solution (Ye et al., 2012). Contrasting this are compact conformations in which defined interfaces are formed. In K48-linked diUb (Varadan et al., 2002), resonances of proximal and distal Ub moieties adopt distinctly different chemical shift positions (splitting) because of their different chemical environment.

**Figure 3:**
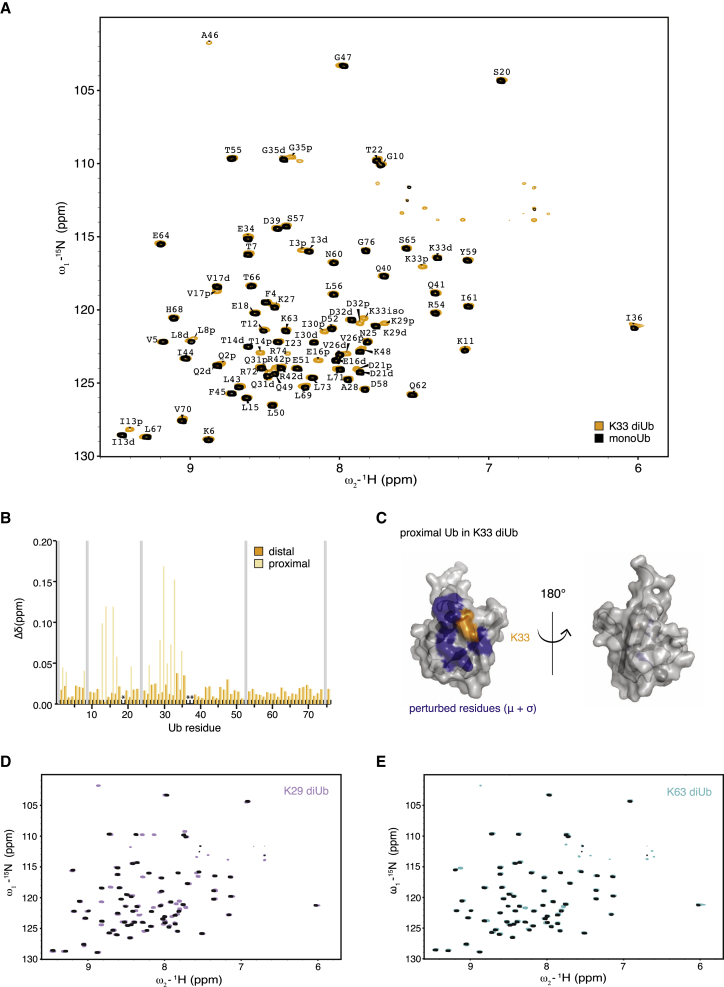
NMR Analysis of K29/K33 Chains (A) BEST-TROSY spectra for ^15^N-K33 diUb (orange) and ^15^N-monoUb (black). Complete assignment of resonances from the proximal (p) or distal (d) moieties from a ^13^C, ^15^N-K33 diUb sample are shown. (B) Chemical shift perturbation of distal (orange) and proximal (beige) resonances with respect to monoUb. Grey bars, exchange-broadened resonances; asterisks, proline residues. (C) Resonances that display a perturbation of more than 1 σ are mapped onto the surface of monoUb (purple) and cluster around the K33 residue (orange). No significant perturbations were observed on the distal Ub moiety, consistent with an open conformation of K33 diUb. (D) BEST-TROSY spectra for ^15^N K29 diUb (purple) and monoUb (black). (E) BEST-TROSY spectra for ^15^N K63 diUb (cyan) and monoUb (black).

We assembled K33-linked diUb from ^13^C, ^15^N-labeled monoUb and measured 2D band-selective excitation short transient transverse relaxation-optimized spectroscopy (BEST-TROSY) spectra, revealing well dispersed peaks similar to monoUb (Figure 3A). Assignment of the spectra revealed splitting of 16 resonances. The small chemical shift perturbations (CSPs) in all split resonances could be attributed to the proximal Ub, whereas the distal Ub was unperturbed (Figure 3B). Mapping of the perturbed residues on Ub revealed a small region surrounding the isopeptide bond at K33 (Figure 3C). Almost identical spectra were obtained for ^15^N-labeled, K29-linked diUb, in which 19 resonances were split and mildly perturbed (Figure 3D). Both diUb spectra resembled the K63 diUb spectrum (Varadan et al., 2004; Figure 3E). Together, this indicates that both K29- and K33-linked diUb do not form defined interfaces in solution but, rather, exist in open conformations.

### TRABID K29/K33 DUB Specificity Is Retained with Longer Chains

**Figure 4:**
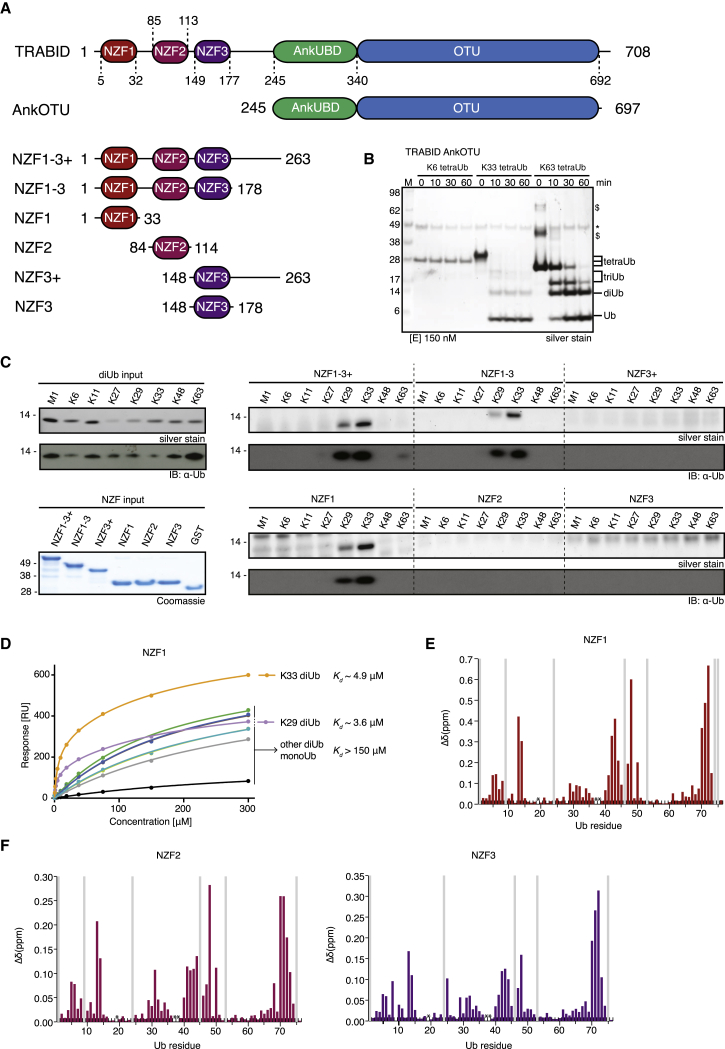
Characterization of TRABID Specificity (A) Domain structure of human TRABID. The AnkOTU fragment has been characterized in detail in Licchesi et al. (2012). Boundaries of the NZF domain fragments analyzed here are shown. (B) Deubiquitination assay of TRABID AnkOTU against K6-, K33-, and K63-linked tetraUb. See Figure S4A for a reaction at a lower DUB concentration. (C) Pull-down analysis of NZF fragments with a panel of diUb covering all linkage types. Left: the input chains and GST-NZF constructs used. Right: pull-down analysis shown by silver stain and anti-Ub western blot. See Figure S4B for additional controls. (D) SPR binding experiment of NZF1 to monoUb and the eight different diUb species with error bars representing SEs. K_d_ values derived from two experiments are shown. See Figure S4C for best-fit parameters and values of SEs. (E) NMR analysis of isolated NZF1 binding to ^15^N-labeled monoUb. The chemical shift perturbation for Ub from binding to 600 μM of NZF1 is shown. Grey bars, exchange-broadened residues; asterisks, proline residues. See Figures S4E–S4G for titration data. (F) NMR analyses as in (E) but for NZF2 and NZF3.

TRABID, a DUB from the OTU family, is the only known protein to date that acts specifically on K29- and K33-linked Ub chains (Licchesi et al., 2012; Mevissen et al., 2013; Figure 4A). In TRABID, a C-terminal OTU domain of the A20 subfamily is preceded by an ankyrin repeat Ub binding domain (AnkUBD) that enables a non-specific OTU domain to preferentially cleave K29- and K33-linked diUb (Licchesi et al., 2012). This construct hydrolyzes K63-linked chains with 40-fold lower activity compared to K29 chains (Virdee et al., 2010). Because of the necessity for chemical synthesis of K27, K29, and K33 linkages, these experiments were so far confined to diUb cleavage. We now confirm that the specificity of TRABID AnkOTU also holds true for longer tetraUb chains. TRABID cleaved K33-linked tetraUb with a significantly higher activity compared with K63-linked chains (Figures 2C, 2D, and 4B; Figure S4A).

### TRABID NZF1 Specifically Binds K29- and K33-Linked Chains

In addition to the C-terminal AnkOTU catalytic domain, TRABID contains three N-terminal NZF domains (Figure 4A) that bind polyUb and are important, together with the AnkUBD, for TRABID localization to characteristic punctate structures in cells (Licchesi et al., 2012). Surprisingly, analyzing the preference of the N-terminal NZF domains in pull-down experiments revealed the specificity of the 3xNZF module (aa 1–263 or 1–178) for K29- and K33-linked diUb, whereas K63-diUb binding was barely detectable (Figure 4C; Figure S4B). This resembled the cleavage specificity of the AnkOTU catalytic domain (Figure 4B; Licchesi et al., 2012). Pull-down experiments with individual NZF domains showed that K29/K33 specificity could be attributed entirely to the N-terminal NZF1 domain (aa 1–33), which bound these chains as well as the 3xNZF modules but did not interact with K63-linked diUb.

To measure affinities, we established a surface plasmon resonance (SPR)-based binding assay in which monoUb and all types of diUb were immobilized, and NZF1 binding was detected by SPR (Figure 4D). Of the nine datasets, seven were fitted to a one-site binding model with NZF1 affinities between 190–370 μM (Figures S4C and S4D). For K29- and K33-linked diUb, fitting to a one-site model resulted in high residuals, and data were fitted to a two-site binding model, revealing significantly higher affinities (K_d_^high^ 3.6 and 4.9 μM, respectively, and 180/200 μM affinities for K_d_^low^) (Figure S4C). The high binding affinities of K29- and K33-linked diUb were consistent with the pull-down experiments (Figure 4C).

Curiously, NZF2 and NZF3 did not bind diUb in pull-down assays. To understand whether these domains can bind Ub, binding studies were performed by NMR using ^15^N-labeled Ub. NZF1 interacts with monoUb, leading to chemical shift perturbation maps that show the characteristic profile for interactions via the Ub Ile44 patch (Figure 4E; Figure S4E). This is consistent with the known binding mode of NZF domains first derived for Npl4 (Alam et al., 2004), and mapping of perturbed residues on Ub suggests similar interactions (Figure S4E). Titrations of NZF2 and NZF3 resulted in similar CSP profiles (Figure 4F; Figure S4E). Using NMR titration experiments, we derived binding constants for the monoUb-NZF interactions, with K_d_ values of ∼440 μM for NZF1, ∼1 mM for NZF2, and ∼540 μM for NZF3 (Figures S4F and S4G), which is in a typical range for monoUb binding to small UBDs. Although this showed that all NZF domains are functional in Ub binding, it did not explain why only NZF1 showed K29/K33 specificity.

### Structure of a K33-Linked Ub Polymer Bound to TRABID NZF1

**Figure 5:**
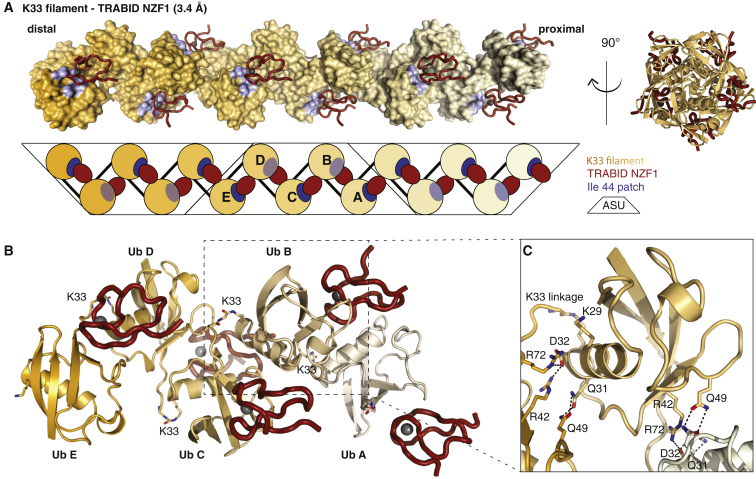
Structure of K33 Filaments Bound to NZF1 (A) Structure of the K33-linked Ub filament as observed in the crystal, showing three adjacent asymmetric units (black outline). Ub molecules are shown as a surface representation with a gradient from orange (distal) to beige (proximal), and Ile44 patches are indicated in blue. NZF1 is shown as a red ribbon with gray Zn^2+^ atoms. A schematic is shown below. Right: view of the filament down the 5-fold symmetry axis. ASU, asymmetric unit. (B) Content of the asymmetric unit, colored as in (A). K33 isopeptide linkages are shown as stick representations; see Figure S5C for electron density. (C) Close-up view of one Ub in the filament, showing interacting residues as a stick representation. Hydrogen bonds are shown as black dashed lines. The K33 and K29 side chains are also shown.

To understand the underlying molecular basis for the K29/K33 specificity of TRABID NZF1, we crystallized the complex with K33-diUb and determined a structure to 3.4 Å resolution. High solvent content (67%, Matthews coefficient ∼ 3.8) led to high-contrast maps (Figure S5A) and allowed building of a complete model with good statistics (Table 1). It was immediately apparent that the arrangement of Ub molecules in the crystal lattice generated seemingly infinite helical polymers (Figures 5A and 5B; Figure S5B). The K33-filament forms a helix with 5-fold symmetry. The helix turns twice between the first and sixth molecule (Figure 5A). The asymmetric unit contains five Ub molecules and five NZF domains (Figures 5A and 5B). The electron density for K33 linkages can be discerned for one isopeptide bond (Figure S5C). The electron density for the isopeptide bonds is weak because of flexibility but also because diUb was crystallized, and each linkage in the asymmetric unit is only at half occupancy. K29 is in close proximity to the tail of the distal Ub, and it is conceivable that K29-linked polymers interact with TRABID NZF1 in a similar manner and can form similar filaments. This was supported by NMR experiments where NZF1 was added to either ^15^N-labeled K33- or K29-linked diUb. NZF1 binding leads to chemical shift perturbations along the same face of the proximal Ub moieties, indicating that the overall orientation of the proximal Ub is similar (Figure S5D).

Interactions between Ub molecules are identical along the filament and involve exclusively polar contacts (Figure 5C). A distal Ub interacts with the Ub helix of a proximal Ub, forming hydrogen bonds between Gln49 (distal Ub) and Gln31 (proximal Ub) and charged interactions between Arg42 and Arg72 (distal Ub) and Asp32 (proximal Ub) (Figure 5C). This exposes the hydrophobic Ile44 and Ile36 patches of each Ub molecule and enables binding of one NZF domain to each Ile44 patch along the filament (Figure 5A).

### Explaining the Specificity of TRABID NZF1 for K33 Linkages

**Figure 6:**
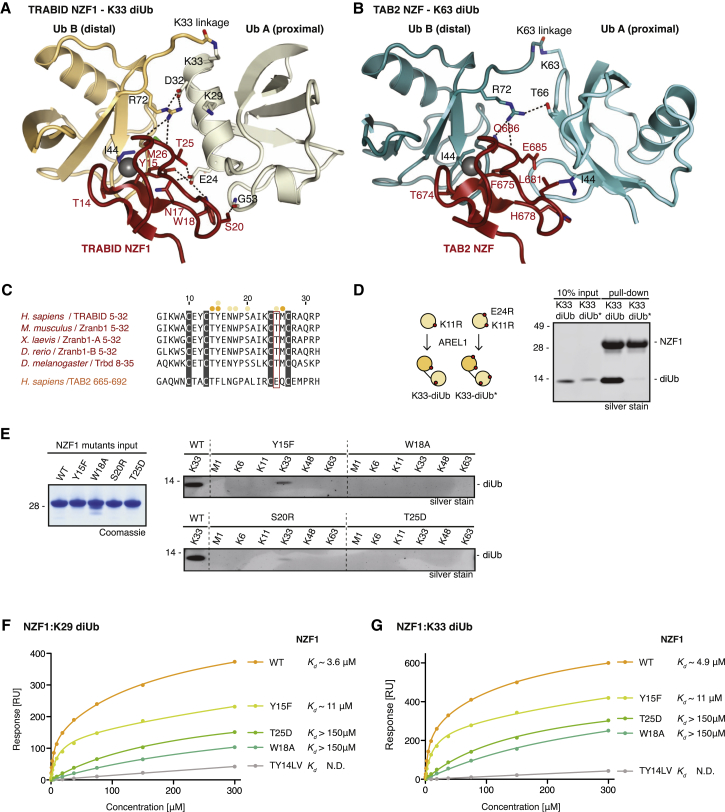
Explaining the K29/K33 Specificity of TRABID NZF1 (A) Detailed view of the interactions between TRABID NZF1 (red) and K33-linked diUb (orange/beige). Interacting residues are labeled, and hydrogen bonds are shown as black dashed lines. (B) As in (A) for the TAB2 NZF interaction with K63-linked diUb (cyan). (C) Sequence alignment of TRABID NZF1 from a diverse range of species and human TAB2 NZF domains. Interacting residues are indicated with orange (distal Ub) and beige (proximal Ub) dots. Thr25 in TRABID NZF1 is replaced with Glu685 in TAB2 NZF, which would prevent K29/K33 binding in TAB2 NZF. (D) Left: Ub chains were assembled into K33 diUb with AREL1 using K11R or K11R/E24R Ub. Right: pull-down assays with TRABID NZF1 and diUb variants. (E) Pull-down assays as in Figure 4C for TRABID NZF1 mutants. (F and G) SPR binding experiment of NZF1 and its mutants against K29 diUb (F) and K33 diUb (G) with the respective K_d_ values indicated. SEs from two experiments are shown as error bars. See Figure S6F for values of SEs and best-fit parameters.

Consistent with other linkage-specific NZF domains, NZF1 of TRABID forms a bidentate interaction across the distal and proximal Ub moieties (Figures 5B and 6A). This has been seen previously for the TAB2 and HOIL-1L NZF domains, which interact specifically with K63- and M1-linked diUb, respectively (Figure 6B; Figure S6A; Kulathu et al., 2009; Sato et al., 2009, 2011), and can be superimposed with TRABID NZF1 with low root-mean-square deviations (RMSDs) (0.5–0.6 Å). TRABID NZF1 binds the distal Ub at the Ile44 patch via the canonical NZF interaction involving Thr14, Tyr15, and Met26 (Figure 6A). This binding mode through the T-Y/F-Φ motif is conserved in all NZF interactions described to date (Figure S6B; Alam et al., 2004) and is consistent with the NMR interaction data in Figures 4E and 4F (see above). The proximal Ub is bound by TRABID NZF1 in an unusual way, at a binding site involving the start of the Ub α helix and two nearby loop regions (Figure 6A). In this interaction, Ub Glu24 makes key interactions with a complementary pocket on NZF1 formed by Tyr15, Asn17, Trp18, and Thr25. The Ub Glu24 side chain can form hydrogen bonds with side chains of these four residues (Figure 6A). In addition, the solvent-exposed TRABID NZF1 Trp18 side chain forms apolar contacts with the Asp52-Gly53 loop of the proximal Ub, and NZF1 Ser20 forms a hydrogen bond with the Gly53 carbonyl group (Figure 6A). All interacting residues in NZF1 are conserved in evolution (Figure 6C).

A comparison of the TAB2 and HOIL-1L diUb complexes reveals how NZF domains have achieved their specificity. Although the canonical interaction with a distal Ub is conserved, the proximal Ub is rotated in each complex to form distinct interactions with a second patch on the NZF domain. In the case of TAB2, the second interaction with a proximal Ub is also via the Ile44 patch (Kulathu et al., 2009; Sato et al., 2009; Figure 6B). In HOIL-1L, a short helical NZF extension contributes the secondary contacts, which are mediated by the Phe4 patch of the proximal Ub (Sato et al., 2011; Figure S6A). Superposition of structures reveals why TAB2 is unable to bind the K33 filament: Glu685 would clash directly with Glu24 of the proximal Ub (Figure S6C). Similarly, TRABID NZF1 Trp18 clashes with the proximal Ub when the TAB2-K63 diUb complex is superimposed (Figure S6D) (although mutation of this residue did not enable high-affinity K63 diUb binding; see below). Finally, the structure also reveals why TRABID NZF2 and NZF3 are unable to bind K33 polymers: Ser20 in NZF1 is replaced by Lys or Arg residues in NZF2/3 (Figure S6E), which affects binding (Figure 6E; see below). However, mutation of Lys165 in NZF3 to Ser did not enhance binding to K29/K33 chains (data not shown), suggesting that the remaining differences play a role as well. It is fascinating that, given their small size, NZF domains have evolved so many distinct binding modes to recognize different linkage types.

### Validation of the TRABID-K33 Chain Interaction

To validate the interaction between TRABID NZF1 and K33-diUb biochemically, we mutated residues in the interfaces. We assembled K33-linked diUb from a Ub K11R/E24R mutant that would abrogate its interactions with the proximal interface of TRABID NZF1. Indeed, TRABID NZF1 is unable to pull down K11R/E24R diUb (Figure 6D), confirming that this Ub residue, which has not been implicated in any other Ub interaction known to us, is important for TRABID NZF1 binding.

Next, NZF1 was mutated (Figures 6E–6G; Figure S6F). NZF1 W18A and T25D were unable to interact with K33-linked diUb in pull-down experiments, and Y15F and S20R significantly weakened binding compared with wild-type NZF1 (Figure 6E). SPR measurements for these mutants interacting with K29- or K33-linked diUb revealed that, although Y15F had to be fitted to a two-site binding model with a lower K_d_^high^ (11 μM), W18A and T25D fit well with a one-site binding model, indicating that they interacted only via the Ub Ile44 patch (Figures 6F and 6G; Figure S6F; Supplemental Experimental Procedures). No binding could be detected when mutating the canonical Thr14/Tyr15 (to Leu/Val, termed TY14LV), consistent with disruption of the Ile44 patch interaction.

This shows the importance of these residues for NZF1 Ub interactions, validates the observed binding mode in the structure for K33-diUb, and further confirms a similar binding mode for K29-linked chains (Figures 5 and 6). Moreover, this emphasizes that conserved residues on previously unknown proximal binding sites in NZF domains (and perhaps other small UBDs) can furnish UBDs with chain preference.

### Localization of Inactive TRABID to Ub-Rich Puncta Relies on NZF1 Binding to Atypical Ub Chains

**Figure 7:**
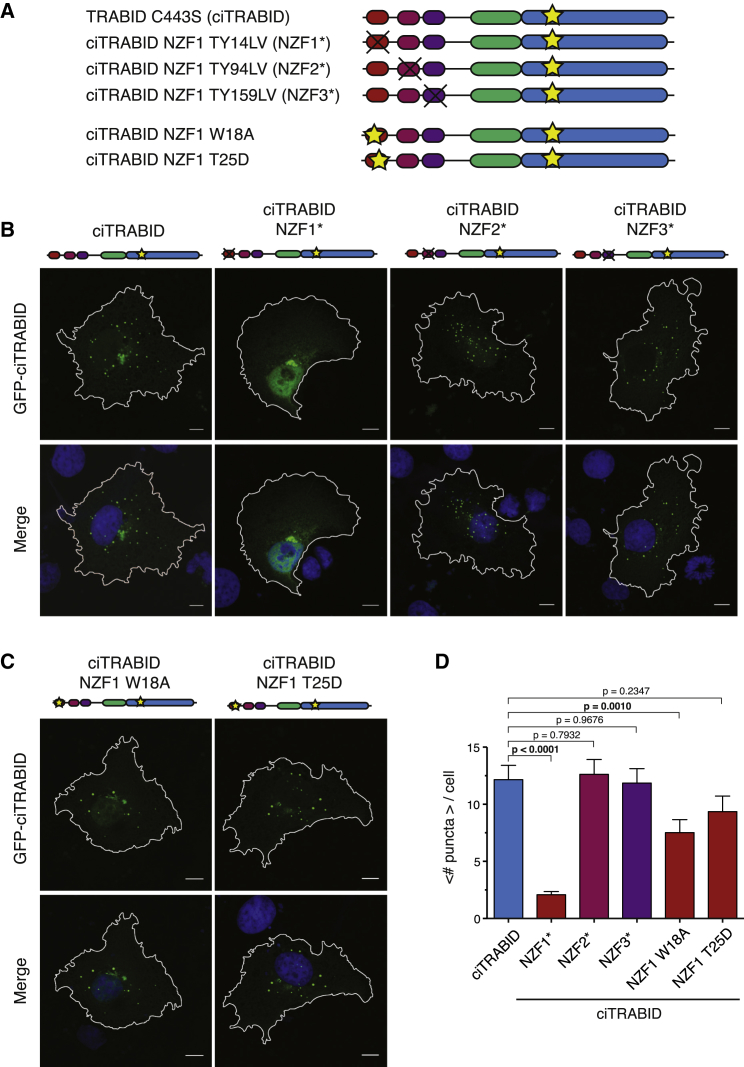
Localization of Catalytically Inactive TRABID Mutants in Cells (A) Constructs used in localization experiments for GFP-TRABID fusions. Yellow stars indicate single amino acid substitutions, whereas black crosses denote two amino acid substitutions that abrogate Ub binding in the respective domain. (B) Localization of catalytically inactive full-length GFP-TRABID (ciTRABID) constructs. GFP-ciTRABID localizes to distinct puncta in COS-7 cells. Mutations in this background that abrogate Ub binding of NZF1 (NZF1^∗^) lead to a significant decrease in the number of dots, whereas the equivalent mutations in NZF2 (NZF2^∗^) or NZF3 (NZF3^∗^) do not lead to a change in the number of puncta. Cartoon representations of the constructs are shown as in (A). Scale bars, 10 μm. (C) The same experiment with single amino acid substitutions in the proximal Ub binding site of NZF1. (D) Statistical analysis of experiments in (B) and (C) with an average number of puncta per cell for the different mutants and corresponding SEs. p Values are given in reference to the ciTRABID mutant, and significant values (α < 0.05) are shown in boldface. Error bars represent SEs.

Catalytically inactive TRABID C443S (ciTRABID) localizes to Ub-rich punctate structures in cells, and this depends on its Ub-binding capability (Licchesi et al., 2012; Tran et al., 2008). TRABID contains at least six independent Ub binding interfaces: at least four in the 3xNZF module, one in the AnkUBD, and at least one in the catalytic domain. Because our biochemical analysis indicated that NZF1 provides TRABID with high-affinity binding for K29/K33 chains, we assessed how important individual NZF domains are for ciTRABID localization to puncta (Figure 7).

Mutation of the canonical Ub binding site in NZF1 (NZF1^∗^; Figure 7A) led to a diffuse (mostly nuclear) ciTRABID localization without puncta (Figure 7B). In contrast, the same mutation in NZF2 or NZF3 (NZF2^∗^ and NZF3^∗^, respectively) showed identical punctate pattern as ciTRABID, with a similar number of dots (Figures 7B and 7C; Figure S7A). This shows that Ub binding by NZF1 is crucial for forming punctate structures in the ciTRABID background. We also tested whether the identified mutants in the proximal Ub binding site of NZF1 are defective in punctum formation. ciTRABID W18A and ciTRABID T25D showed a reduction in the number of puncta per cell (Figures 7B and 7C; Figure S7A). This is consistent with the distal Ub binding site still being intact and maintaining residual low-affinity Ub binding capability. However, the significant reduction of dots with ciTRABID W18A suggests that the NZF1:K29/K33 interface promotes punctum formation (Figure 7D).

## Discussion

Chain linkage profiling by AQUA mass spectrometry is a powerful strategy to discover the missing ligases for atypical chains and to provide mechanistic insights into Ub chain assembly. Because of their mechanism of E2-independent linkage determination, HECT E3 ligases are good candidates to assemble atypical chains, and, although several have been suggested to assemble atypical linkages, only a subset have been characterized biochemically (Tran et al., 2013; You and Pickart, 2001). We show here that AREL1 predominantly assembles K33 linkages in free chains and on substrate proteins. Together with UBE3C, which has been reported to generate K48 and K29 linkages (You and Pickart, 2001), we provide a protocol to generate pure K29- and K33-linked polyUb enzymatically for in vitro analysis.

Our protocol to generate WT K29- or K33-linked polyUb relies on the recently discovered linkage specificity in DUBs, and we used these enzymes preparatively to remove unwanted linkage types in chains. Our redesigned forms of K48-specific OTUB1 and K63-specific AMSH are highly active and have proven to be useful for this purpose. Together with Ub chain restriction (UbiCRest) analysis (Mevissen et al., 2013), this highlights the utility of linkage-specific DUBs in studying the Ub system.

K29- and K33-linked chains are flexible and able to adopt multiple conformations, much like the remaining chain types (Ye et al., 2012). Although the diUb crystal structure has captured a compact conformation of K33-linked diUb, solution studies suggest open conformations for both chain types without formation of defined interfaces, as reported for chemically assembled K33-linked chains (Dixon et al., 2013).

Ub binding proteins can stabilize chain conformations (Ye et al., 2012), and it is therefore important to understand how polyUb is recognized by UBDs in a linkage-specific fashion. Our discovery of K29/K33 specificity in the N-terminal TRABID NZF1 domain enabled further insights into linkage-specific UBDs. NZF domains are small zinc-binding folds with remarkable linkage specificity that is achieved by bidentate interactions, whereby the ∼30-aa NZF fold intercalates between and interacts with two Ub molecules. The TRABID NZF1 contacts the canonical Ile44 hydrophobic patch on the distal Ub and an unusual surface on the proximal Ub surrounding Glu24 of the Ub helix. The observed binding mode is validated by mutational analysis, which indicates that it may be shared between K29- and K33-linked chains, explaining TRABID NZF1 cross-specificity. The structure also explains why TAB2 or TRABID NZF2 and NZF3 are unable to bind K29/K33-linked chains in a similar manner.

A remarkable feature of the structure is the assembly of the K33 diUb with NZF1 into filaments that make up the entire crystal. In these filaments, each Ub-Ub contact is identical, each NZF domain binds two Ub molecules, and each Ub binds two NZF domains. The helical Ub filament bound to NZF1 domains provides an immediate model for interactions with the TRABID 3xNZF module, which, despite the lack of K29/K33 specificity in NZF2 and NZF3, could assemble on a Ub filament (Figure S7B). This is enabled by flexible linkers that vary in sequence and length but have a minimal length of 35 aa (NZF1-NZF2) and 28 aa (NZF2-NZF3) throughout evolution (Figure S7C). Such linkers would easily be able to bridge the space between adjacent NZF domains when binding a K29/K33 filament (Figure S7B).

Further studies by complementary techniques will be required to see whether K29- and K33-linked chains indeed form filaments in the presence of NZF1 in vivo. We have previously described the accumulation of catalytically inactive TRABID into characteristic Ub-containing puncta in cells, which depends on functional NZF domains (Licchesi et al., 2012). Here we extend these studies to show that, indeed, NZF1 and its K29/K33-specific binding mode are important for punctum formation. The cellular structures covered with K29/K33 chains that lead to punctum formation are intriguing and require further investigation.

Nonetheless, some new roles of K33-linked chains are emerging. AREL1 has been reported to ubiquitinate cytosolic inhibitor of apoptosis (IAP) antagonists, including SMAC, HtrA2, and ARTS, which leads to their proteasomal degradation (Kim et al., 2013). We show here that AREL1 polyubiquitinates SMAC and HtrA2 with >80% of K33 linkages in vitro. It will be interesting to see whether K33-linked chains can be linked to antiapoptotic signaling.

In cells, K33 chains have been found on AMPK kinases (together with K29-linked chains; Al-Hakim et al., 2008), on T cell receptor (TCR) ζ (Huang et al., 2010), and on Coronin-7 (Yuan et al., 2014). The latter study used an elegant Ub replacement strategy where cells express Ub K33R instead of WT Ub and revealed roles of this chain type in post-Golgi transport (Yuan et al., 2014). Interestingly, these reports and our previous work on TRABID (Licchesi et al., 2012) agreed that K33-linked chains are likely non-degradative, which is consistent with this chain type not being significantly enriched upon proteasomal inhibition (Kim et al., 2011). The ability of K33 chains to act as a proteasomal degradation signal requires further study.

Such in vivo studies of atypical chain types can now be supplemented with powerful biochemical tools reported here. The TRABID NZF1 domain could serve as an excellent tool in future studies, e.g., when used as a Ub chain sensor (Sims et al., 2012; van Wijk et al., 2012) or as a K29/K33-specific affinity reagent (Hjerpe et al., 2009). The availability of K29- and K33-linked polymers and new affinity reagents will enable a better understanding of these uncharacterized Ub signals.

## Experimental Procedures

Please see the Supplemental Experimental Procedures for more detailed information.

### Protein Production

The His6-SUMO-AREL1 (436–823) and His6-SUMO-UBE3C (693–1083) constructs (both from the pOPIN-S vector) and the His6-GST-TRABID NZF construct (from the pOPIN-K vector) were expressed in Rosetta2 (DE3) pLacI cells and purified by affinity chromatography. Tags were removed by incubation with SENP1 or 3C protease. Further purification was performed by anion exchange and/or size exclusion chromatography.

### Ub Chain Composition Mass Spectrometry Analysis

Ub chains were separated on a NuPAGE 4%–12% gradient gel (Invitrogen) before in-gel digestion with trypsin and the addition of Ub AQUA peptide internal standards according to Kirkpatrick et al. (2006). 10 μl of each sample was directly injected onto an EASY-Spray reverse-phase column (C18, 3 μm, 100 Å, 75 μm × 15 cm) using a Dionex UltiMate 3000 high-pressure liquid chromatography system (Thermo Fisher Scientific) and analyzed on a Q-Exactive mass spectrometer (Thermo Fisher Scientific) using parallel reaction monitoring (PRM), similar to Tsuchiya et al. (2013). Data were analyzed further according to Kirkpatrick et al. (2006).

### K29 Chain Generation

K29-linked polyUb was assembled from 3 mM Ub, 1 μM E1, 10 μM UBE2L3, and 32 μM His6-SUMO UBE3C (aa 693–1083) in buffer containing 10 mM ATP, 10 mM MgCl_2_, 40 mM Tris (pH 8.5), 100 mM NaCl, 0.6 mM DTT, and 10% (v/v) glycerol overnight at 37°C. After precipitation of enzymes by perchloric acid (0.25% [v/v]), unanchored chains were buffer-exchanged into 50 mM Tris (pH 7.4), 150 mM NaCl, and 4 mM DTT and treated with OTUB1^∗^ (1 μM), AMSH^∗^ (1 μM), and Cezanne (400 nM) for 60 min at 37°C. A second round of acid precipitation and cation exchange chromatography was used for purification.

### K33 Chain Generation

K33-linked polyUb was assembled like K29-linked chains from a reaction that contained 36 μM AREL1 (aa 436–823) instead of UBE3C. The addition of 10% (v/v) glycerol in the reaction buffer prevented AREL1 precipitation during the reaction.

### Pull-Down Assays

Pull-down assays were performed as described previously (Kulathu et al., 2009). Proteins were visualized by silver staining using the Silver Stain Plus kit (BioRad) according to manufacturer’s protocols or by western blotting using a rabbit anti-Ub antibody (Millipore).

### Nuclear Magnetic Resonance Studies

NMR experiments were performed in NMR PBS (18 mM Na_2_HPO_4_, 7 mM NaH_2_PO_4_ (pH 7.2), and 150 mM NaCl) with 5% D_2_O added as a lock solvent. NMR acquisition was carried out at 298 K on a Bruker Avance III 600 MHz spectrometer equipped with a cryogenic triple resonance TCI probe. Topspin (Bruker) and Sparky (Goddard & Kneller, University of California San Francisco; http://www.cgl.ucsf.edu/home/sparky/) software packages were used for data processing and analysis, respectively. ^1^H,^15^N 2D BEST-TROSY experiments (Favier and Brutscher, 2011) allowed the calculation of weighted chemical shift perturbation using the equation √(Δ^1^H)^2^+((Δ^15^N)^2^/5). K_d_ values for NZF-Ub interactions were determined according to Williamson (2013).

### Crystallization, Data Collection, and Refinement

Crystals of K33-linked diUb, triUb, and of the TRABID NZF1-K33 diUb complex were grown by sitting drop vapor diffusion. Diffraction data were collected at Diamond Light Source beamlines I03 and I24, and the structures were solved by molecular replacement and refined to the final statistics in Table 1.

## Author Contributions

M.A.M. performed all experiments relating to K33 binding to TRABID, and P.R.E. performed experiments relating to HECT E3 ligases and structural characterization of free chains. K.N.S. performed all mass spectrometry analyses. M.S. performed localization studies, and J.N.P. contributed improved DUBs. J.L.W. and S.M.V.F. performed NMR analyses. D.K. directed the research and wrote the manuscript with input from all authors.

## Figures and Tables

**Table 1:** Data Collection Statistics

	K33 diUb	K33 triUb	TRABID NZF1-K33 diUb
**Data Collection**			

Beamline	Diamond I03	Diamond I24	Diamond I24
Space group	*I* 4	*P* 2_1_ 2_1_ 2_1_	*C* 2
*a*, *b*, *c* (Å)	113.08, 113.08, 103.90	28.42, 42.48, 50.52	98.38, 126.51, 78.09
α, β, γ (°)	90.00, 90.00, 90.00	90.00, 90.00, 90.00	90.00, 103.38, 90.00
Wavelength	0.9763	0.9686	0.9686
Resolution (Å)	45.47–1.85 (1.89–1.85)^a^	23.62–1.68 (1.72–1.68)	38.59–3.40 (3.67–3.40)
*R*_merge_	4.5 (44.3)	5.1 (77.6)	10.9 (56.2)
*I*/σ*I*	11.9 (2.5)	19.9 (2.7)	6.6 (2.0)
Completeness (%)	99.8 (100)	99.8 (99.5)	99.9 (100)
Redundancy	3.5 (3.5)	7.3 (7.7)	3.4 (3.4)

**Refinement**			

Resolution (Å)	45.47–1.85	23.62–1.68	38.59–3.40
No. of reflections	55,562	7,363	12,783
*R*_work_/*R*_free_	22.9/27.1	19.4/22.5	18.0/22.2

**No. of Atoms**			

Protein	4,788	596	4,076
Ligand/ion	84		5
Water	128	49	

**B Factors**			

Wilson *B*	33.77	26.49	83.17
Protein	70.20	35.00	106.68
Ligand/ion	67.50		83.84
Water	45.90	43.17	

**RMSDs**			

Bond lengths (Å)	0.005	0.002	0.002
Bond angles (°)	0.930	0.748	0.603
Ramachandran statistics (favored /allowed/outliers)	99.0/1.0/0.0	100.0/0.0/0.0	98.8/1.2/0.0

a Numbers in brackets are for the highest-resolution bin.

## Accession Numbers

Coordinates and structure factors for K33-linked di- and triUb and for TRABID NZF1-K33 diUb have been deposited with the protein data bank under accession codes 5AF4, 5AF5, and 5AF6.

## Supplemental Information

Document S1. Supplemental Experimental Procedures and Figures S1–S7

Document S2. Article plus Supplemental Information

## References

[bib1] Al-Hakim A.K., Zagorska A., Chapman L., Deak M., Peggie M., Alessi D.R. (2008). Control of AMPK-related kinases by USP9X and atypical Lys(29)/Lys(33)-linked polyubiquitin chains. Biochem. J..

[bib2] Alam S.L., Sun J., Payne M., Welch B.D., Blake B.K., Davis D.R., Meyer H.H., Emr S.D., Sundquist W.I. (2004). Ubiquitin interactions of NZF zinc fingers. EMBO J..

[bib3] Berndsen C.E., Wolberger C. (2014). New insights into ubiquitin E3 ligase mechanism. Nat. Struct. Mol. Biol..

[bib4] Bremm A., Komander D. (2011). Emerging roles for Lys11-linked polyubiquitin in cellular regulation. Trends Biochem. Sci..

[bib5] Bremm A., Freund S.M.V., Komander D. (2010). Lys11-linked ubiquitin chains adopt compact conformations and are preferentially hydrolyzed by the deubiquitinase Cezanne. Nat. Struct. Mol. Biol..

[bib6] Christianson J.C., Ye Y. (2014). Cleaning up in the endoplasmic reticulum: ubiquitin in charge. Nat. Struct. Mol. Biol..

[bib7] Clague M.J., Barsukov I., Coulson J.M., Liu H., Rigden D.J., Urbé S. (2013). Deubiquitylases from genes to organism. Physiol. Rev..

[bib8] Deshaies R.J., Joazeiro C.A.P. (2009). RING domain E3 ubiquitin ligases. Annu. Rev. Biochem..

[bib9] Dixon E.K., Castañeda C.A., Kashyap T.R., Wang Y., Fushman D. (2013). Nonenzymatic assembly of branched polyubiquitin chains for structural and biochemical studies. Bioorg. Med. Chem..

[bib10] Favier A., Brutscher B. (2011). Recovering lost magnetization: polarization enhancement in biomolecular NMR. J. Biomol. NMR.

[bib11] Hershko A., Ciechanover A. (1998). The ubiquitin system. Annu. Rev. Biochem..

[bib12] Hjerpe R., Aillet F., Lopitz-Otsoa F., Lang V., England P., Rodriguez M.S. (2009). Efficient protection and isolation of ubiquitylated proteins using tandem ubiquitin-binding entities. EMBO Rep..

[bib13] Hospenthal M.K., Freund S.M.V., Komander D. (2013). Assembly, analysis and architecture of atypical ubiquitin chains. Nat. Struct. Mol. Biol..

[bib14] Huang H., Jeon M.-S., Liao L., Yang C., Elly C., Yates J.R., Liu Y.-C. (2010). K33-linked polyubiquitination of T cell receptor-zeta regulates proteolysis-independent T cell signaling. Immunity.

[bib15] Husnjak K., Dikic I. (2012). Ubiquitin-binding proteins: decoders of ubiquitin-mediated cellular functions. Annu. Rev. Biochem..

[bib16] Iwai K., Fujita H., Sasaki Y. (2014). Linear ubiquitin chains: NF-κB signalling, cell death and beyond. Nat. Rev. Mol. Cell Biol..

[bib17] Kamadurai H.B., Souphron J., Scott D.C., Duda D.M., Miller D.J., Stringer D., Piper R.C., Schulman B.A. (2009). Insights into ubiquitin transfer cascades from a structure of a UbcH5B approximately ubiquitin-HECT(NEDD4L) complex. Mol. Cell.

[bib18] Kamadurai H.B., Qiu Y., Deng A., Harrison J.S., Macdonald C., Actis M., Rodrigues P., Miller D.J., Souphron J., Lewis S.M. (2013). Mechanism of ubiquitin ligation and lysine prioritization by a HECT E3. eLife.

[bib19] Keusekotten K., Elliott P.R., Glockner L., Fiil B.K., Damgaard R.B., Kulathu Y., Wauer T., Hospenthal M.K., Gyrd-Hansen M., Krappmann D. (2013). OTULIN antagonizes LUBAC signaling by specifically hydrolyzing Met1-linked polyubiquitin. Cell.

[bib20] Kim H.C., Huibregtse J.M. (2009). Polyubiquitination by HECT E3s and the determinants of chain type specificity. Mol. Cell. Biol..

[bib21] Kim W., Bennett E.J., Huttlin E.L., Guo A., Li J., Possemato A., Sowa M.E., Rad R., Rush J., Comb M.J. (2011). Systematic and quantitative assessment of the ubiquitin-modified proteome. Mol. Cell.

[bib22] Kim J.B., Kim S.Y., Kim B.M., Lee H., Kim I., Yun J., Jo Y., Oh T., Jo Y., Chae H.D., Shin D.Y. (2013). Identification of a novel anti-apoptotic E3 ubiquitin ligase that ubiquitinates antagonists of inhibitor of apoptosis proteins SMAC, HtrA2, and ARTS. J. Biol. Chem..

[bib23] Kirkpatrick D.S., Hathaway N.A., Hanna J., Elsasser S., Rush J., Finley D., King R.W., Gygi S.P. (2006). Quantitative analysis of in vitro ubiquitinated cyclin B1 reveals complex chain topology. Nat. Cell Biol..

[bib24] Komander D., Rape M. (2012). The ubiquitin code. Annu. Rev. Biochem..

[bib25] Komander D., Clague M.J., Urbé S. (2009). Breaking the chains: structure and function of the deubiquitinases. Nat. Rev. Mol. Cell Biol..

[bib26] Kulathu Y., Komander D. (2012). Atypical ubiquitylation - the unexplored world of polyubiquitin beyond Lys48 and Lys63 linkages. Nat. Rev. Mol. Cell Biol..

[bib27] Kulathu Y., Akutsu M., Bremm A., Hofmann K., Komander D. (2009). Two-sided ubiquitin binding explains specificity of the TAB2 NZF domain. Nat. Struct. Mol. Biol..

[bib28] Licchesi J.D.F., Mieszczanek J., Mevissen T.E.T., Rutherford T.J., Akutsu M., Virdee S., El Oualid F., Chin J.W., Ovaa H., Bienz M., Komander D. (2012). An ankyrin-repeat ubiquitin-binding domain determines TRABID’s specificity for atypical ubiquitin chains. Nat. Struct. Mol. Biol..

[bib29] Maspero E., Valentini E., Mari S., Cecatiello V., Soffientini P., Pasqualato S., Polo S. (2013). Structure of a ubiquitin-loaded HECT ligase reveals the molecular basis for catalytic priming. Nat. Struct. Mol. Biol..

[bib30] Mattiroli F., Sixma T.K. (2014). Lysine-targeting specificity in ubiquitin and ubiquitin-like modification pathways. Nat. Struct. Mol. Biol..

[bib31] Mevissen T.E.T., Hospenthal M.K., Geurink P.P., Elliott P.R., Akutsu M., Arnaudo N., Ekkebus R., Kulathu Y., Wauer T., El Oualid F. (2013). OTU deubiquitinases reveal mechanisms of linkage specificity and enable ubiquitin chain restriction analysis. Cell.

[bib32] Ritorto M.S., Ewan R., Perez-Oliva A.B., Knebel A., Buhrlage S.J., Wightman M., Kelly S.M., Wood N.T., Virdee S., Gray N.S. (2014). Screening of DUB activity and specificity by MALDI-TOF mass spectrometry. Nat. Commun..

[bib33] Rivkin E., Almeida S.M., Ceccarelli D.F., Juang Y.-C., MacLean T.A., Srikumar T., Huang H., Dunham W.H., Fukumura R., Xie G. (2013). The linear ubiquitin-specific deubiquitinase gumby regulates angiogenesis. Nature.

[bib34] Rotin D., Kumar S. (2009). Physiological functions of the HECT family of ubiquitin ligases. Nat. Rev. Mol. Cell Biol..

[bib35] Sato Y., Yoshikawa A., Yamashita M., Yamagata A., Fukai S. (2009). Structural basis for specific recognition of Lys 63-linked polyubiquitin chains by NZF domains of TAB2 and TAB3. EMBO J..

[bib36] Sato Y., Fujita H., Yoshikawa A., Yamashita M., Yamagata A., Kaiser S.E., Iwai K., Fukai S. (2011). Specific recognition of linear ubiquitin chains by the Npl4 zinc finger (NZF) domain of the HOIL-1L subunit of the linear ubiquitin chain assembly complex. Proc. Natl. Acad. Sci. USA.

[bib37] Scheffner M., Huibregtse J.M., Vierstra R.D., Howley P.M. (1993). The HPV-16 E6 and E6-AP complex functions as a ubiquitin-protein ligase in the ubiquitination of p53. Cell.

[bib38] Schulman B.A., Harper J.W. (2009). Ubiquitin-like protein activation by E1 enzymes: the apex for downstream signalling pathways. Nat. Rev. Mol. Cell Biol..

[bib39] Shaid S., Brandts C.H., Serve H., Dikic I. (2013). Ubiquitination and selective autophagy. Cell Death Differ..

[bib40] Sims J.J., Scavone F., Cooper E.M., Kane L.A., Youle R.J., Boeke J.D., Cohen R.E. (2012). Polyubiquitin-sensor proteins reveal localization and linkage-type dependence of cellular ubiquitin signaling. Nat. Methods.

[bib41] Tran H., Hamada F., Schwarz-Romond T., Bienz M. (2008). Trabid, a new positive regulator of Wnt-induced transcription with preference for binding and cleaving K63-linked ubiquitin chains. Genes Dev..

[bib42] Tran H., Bustos D., Yeh R., Rubinfeld B., Lam C., Shriver S., Zilberleyb I., Lee M.W., Phu L., Sarkar A.A. (2013). HectD1 E3 ligase modifies adenomatous polyposis coli (APC) with polyubiquitin to promote the APC-axin interaction. J. Biol. Chem..

[bib43] Tsuchiya H., Tanaka K., Saeki Y. (2013). The parallel reaction monitoring method contributes to a highly sensitive polyubiquitin chain quantification. Biochem. Biophys. Res. Commun..

[bib44] van Wijk S.J.L., Fiskin E., Putyrski M., Pampaloni F., Hou J., Wild P., Kensche T., Grecco H.E., Bastiaens P., Dikic I. (2012). Fluorescence-based sensors to monitor localization and functions of linear and K63-linked ubiquitin chains in cells. Mol. Cell.

[bib45] Varadan R., Walker O., Pickart C., Fushman D. (2002). Structural properties of polyubiquitin chains in solution. J. Mol. Biol..

[bib46] Varadan R., Assfalg M., Haririnia A., Raasi S., Pickart C., Fushman D. (2004). Solution conformation of Lys63-linked di-ubiquitin chain provides clues to functional diversity of polyubiquitin signaling. J. Biol. Chem..

[bib47] Vijay-Kumar S., Bugg C.E., Cook W.J. (1987). Structure of ubiquitin refined at 1.8 A resolution. J. Mol. Biol..

[bib48] Virdee S., Ye Y., Nguyen D.P., Komander D., Chin J.W. (2010). Engineered diubiquitin synthesis reveals Lys29-isopeptide specificity of an OTU deubiquitinase. Nat. Chem. Biol..

[bib49] Wagner S.A., Beli P., Weinert B.T., Nielsen M.L., Cox J., Mann M., Choudhary C. (2011). A proteome-wide, quantitative survey of in vivo ubiquitylation sites reveals widespread regulatory roles. Mol Cell Proteomics.

[bib50] Wickliffe K.E., Williamson A., Meyer H.-J., Kelly A., Rape M. (2011). K11-linked ubiquitin chains as novel regulators of cell division. Trends Cell Biol..

[bib51] Williamson M.P. (2013). Using chemical shift perturbation to characterise ligand binding. Prog. Nucl. Magn. Reson. Spectrosc..

[bib52] Xu P., Duong D.M., Seyfried N.T., Cheng D., Xie Y., Robert J., Rush J., Hochstrasser M., Finley D., Peng J. (2009). Quantitative proteomics reveals the function of unconventional ubiquitin chains in proteasomal degradation. Cell.

[bib53] Ye Y., Rape M. (2009). Building ubiquitin chains: E2 enzymes at work. Nat. Rev. Mol. Cell Biol..

[bib54] Ye Y., Blaser G., Horrocks M.H., Ruedas-Rama M.J., Ibrahim S., Zhukov A.A., Orte A., Klenerman D., Jackson S.E., Komander D. (2012). Ubiquitin chain conformation regulates recognition and activity of interacting proteins. Nature.

[bib55] You J., Pickart C.M. (2001). A HECT domain E3 enzyme assembles novel polyubiquitin chains. J. Biol. Chem..

[bib56] Yuan W.-C., Lee Y.-R., Lin S.-Y., Chang L.-Y., Tan Y.P., Hung C.-C., Kuo J.-C., Liu C.-H., Lin M.-Y., Xu M. (2014). K33-Linked Polyubiquitination of Coronin 7 by Cul3-KLHL20 Ubiquitin E3 Ligase Regulates Protein Trafficking. Mol. Cell.

