# Supplementary material for: Assembly and Specific Recognition of K29- and K33-Linked Polyubiquitin
Source: Mol Cell. 2015 Apr 2;58(1):95–109. doi: 10.1016/j.molcel.2015.01.042 (PMC4386031; doi:10.1016/j.molcel.2015.01.042)
Supplement: Document S1. Supplemental Experimental Procedures and Figures S1–S7 [file mmc1.pdf]

Molecular Cell, Volume *58*

Supplemental Information

Assembly and Specific Recognition of K29- and K33-Linked Polyubiquitin

Martin A. Michel, Paul R. Elliott, Kirby N. Swatek, Michal Simicek, Jonathan N. Pruneda, Jane L. Wagstaff, Stefan M.V. Freund, and David Komander

Figure S1

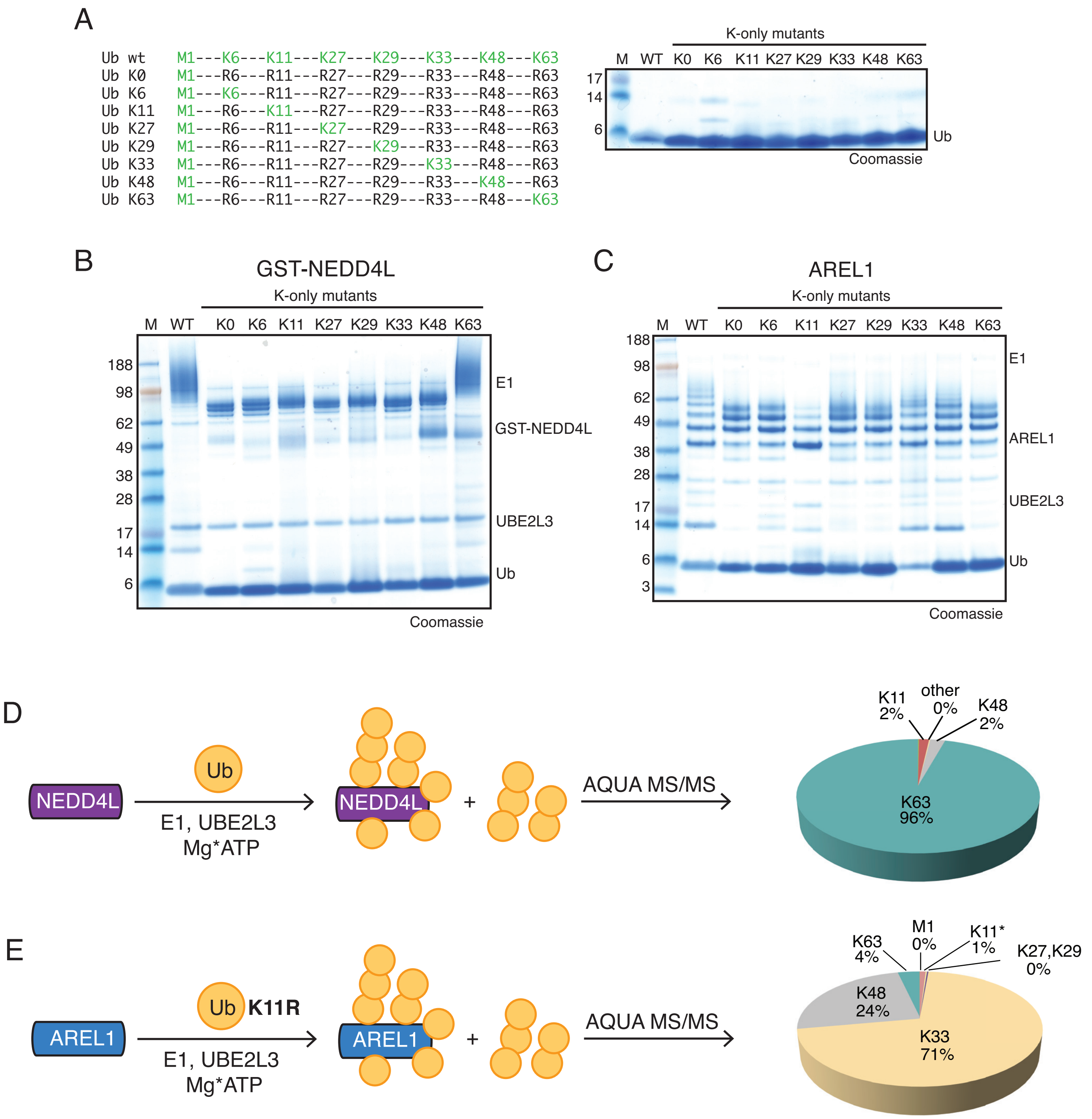
A
Ub wt M1---K6---K11---K27---K29---K33---K48---K63
Ub K0 M1---R6---R11---R27---R29---R33---R48---R63
Ub K6 M1---K6---R11---R27---R29---R33---R48---R63
Ub K11 M1---R6---K11---R27---R29---R33---R48---R63
Ub K27 M1---R6---R11---K27---R29---R33---R48---R63
Ub K29 M1---R6---R11---R27---K29---R33---R48---R63
Ub K33 M1---R6---R11---R27---R29---K33---R48---R63
Ub K48 M1---R6---R11---R27---R29---R33---K48---R63
Ub K63 M1---R6---R11---R27---R29---R33---R48---K63
K-only mutants
M WT K0 K6 K11 K27 K29 K33 K48 K63
17
14
6
Ub
Coomassie
B
GST-NEDD4L
K-only mutants
M WT K0 K6 K11 K27 K29 K33 K48 K63
188
98
62
49
38
28
17
14
6
E1
GST-NEDD4L
UBE2L3
Ub
Coomassie
C
AREL1
K-only mutants
M WT K0 K6 K11 K27 K29 K33 K48 K63
188
98
62
49
38
28
17
14
6
3
E1
AREL1
UBE2L3
Ub
Coomassie
D
NEDD4L
Ub
E1, UBE2L3
Mg*ATP
NEDD4L
+
AQUA MS/MS
K11 2%
other 0%
K48 2%
K63 96%
E
AREL1
Ub K11R
E1, UBE2L3
Mg*ATP
AREL1
+
AQUA MS/MS
K63 4%
M1 0%
K11* 1%
K27,K29 0%
K48 24%
K33 71%

Figure S1 (related to Figure 1). HECT specificity analysis with K-only Ub mutants.

A) Schematic of K-only Ub mutants, and Coomassie-stained SDS-PAGE gel showing input monoUb. **B)** Specificity of GST-tagged NEDD4L (aa 576-955) which is K63 specific (Kamadurai et al., 2009) using a panel of K-only Ub mutants as indicated. Samples were resolved by SDS-PAGE and proteins visualized by Coomassie staining. **C)** Analysis as in **B** for AREL1. **D)** Linkage composition analyzed by AQUA mass-spectrometry for NEDD4L, demonstrating K63-specificity with wt Ub. **E)** Linkage composition analyzed with AQUA mass-spectrometry for AREL1, as in **Figure 1C**, but using Ub K11R (K6-linkage was excluded from the quantitative analysis due to the K11R substitution).

Figure S2

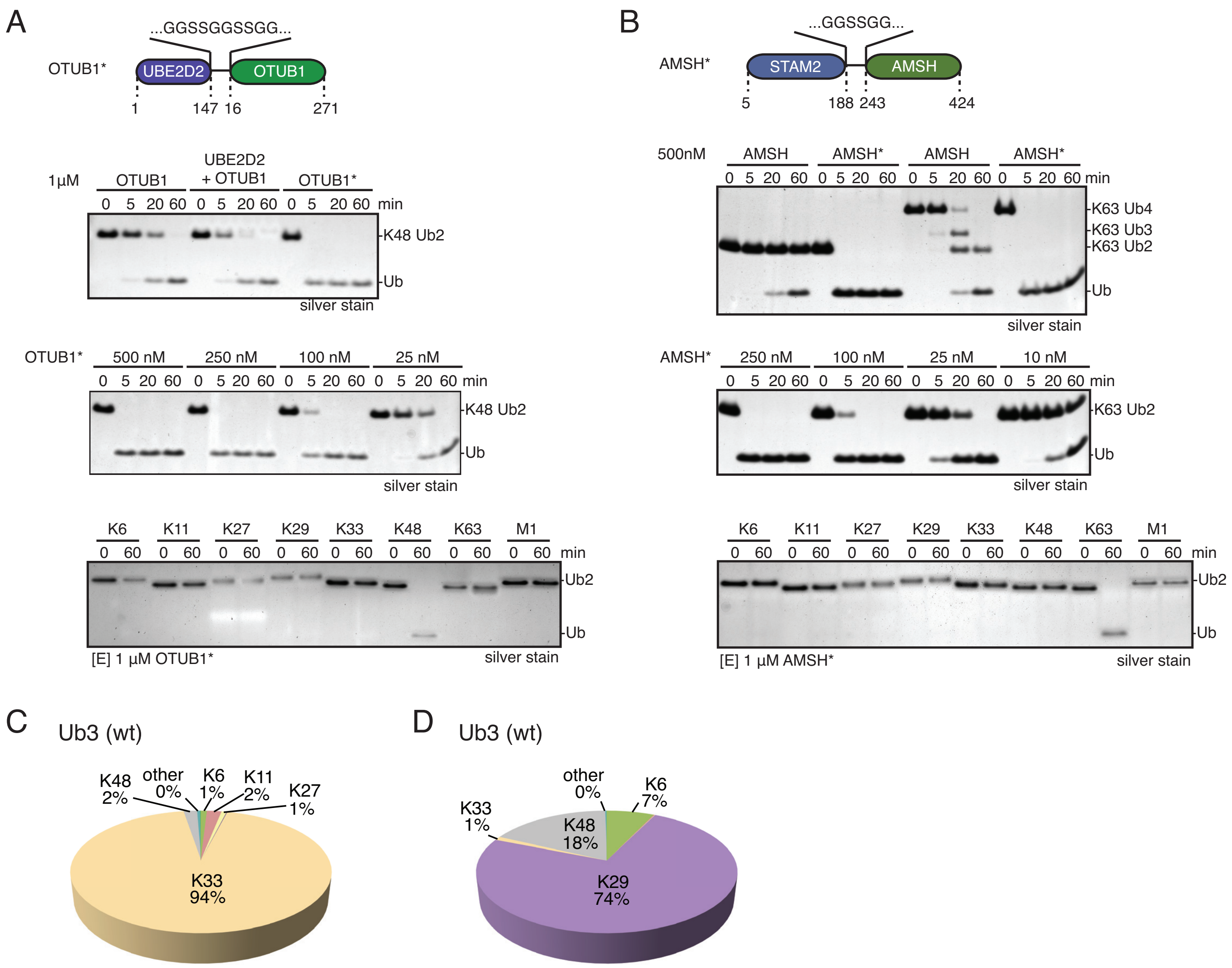
A
...GGSSGGSSGG...
OTUB1*
UBE2D2
OTUB1
1
147 16
271
1µM
OTUB1
UBE2D2 + OTUB1
OTUB1*
0 5 20 60 0 5 20 60 0 5 20 60 min
K48 Ub2
Ub
silver stain
OTUB1*
500 nM
250 nM
100 nM
25 nM
0 5 20 60 0 5 20 60 0 5 20 60 0 5 20 60 min
K48 Ub2
Ub
silver stain
K6 K11 K27 K29 K33 K48 K63 M1
0 60 0 60 0 60 0 60 0 60 0 60 0 60 0 60 min
Ub2
Ub
[E] 1 µM OTUB1*
silver stain
B
...GGSSGG...
AMSH*
STAM2
AMSH
5
188 243
424
500nM
AMSH
AMSH*
AMSH
AMSH*
0 5 20 60 0 5 20 60 0 5 20 60 0 5 20 60 min
K63 Ub4
K63 Ub3
K63 Ub2
Ub
silver stain
AMSH*
250 nM
100 nM
25 nM
10 nM
0 5 20 60 0 5 20 60 0 5 20 60 0 5 20 60 min
K63 Ub2
Ub
silver stain
K6 K11 K27 K29 K33 K48 K63 M1
0 60 0 60 0 60 0 60 0 60 0 60 0 60 0 60 min
Ub2
Ub
[E] 1 µM AMSH*
silver stain
C
Ub3 (wt)
K48 2%
other 0%
K6 1%
K11 2%
K27 1%
K33 94%
D
Ub3 (wt)
other 0%
K6 7%
K33 1%
K48 18%
K29 74%

Figure S2 (related to Figure 2). Purification of unanchored K29/K33 polyUb chains.

A) The UBE2D2-OTUB1 fusion (OTUB1*; see cartoon) is K48-specific and displays significantly higher activity against K48-linked diUb. A deubiquitinase assay is shown of wt OTUB1, OTUB1 mixed with UBE2D2 and the UBE2D2-OTUB1 fusion (OTUB1*) (*top*). (*Middle*) OTUB1* is able to hydrolyze 3 μM diUb at 25 nM enzyme concentration within 60 min. (*Bottom*) OTUB1* is still specific against K48-linked diUb at 1 μM DUB concentration when compared to all other linkage types. **B)** Comparison of AMSH activity versus STAM2-AMSH fusion (AMSH*; see cartoon) against diUb and tetraUb (*top*). AMSH* is able to hydrolyze 3 μM diUb at 25 nM DUB concentration within 60 min (*middle*). (*Bottom*) AMSH* retains its specificity against K63 diUb at 1 μM DUB concentration. **C)** AQUA mass-spectrometry profile for purified wt K33 triUb. **D)** AQUA mass-spectrometry profile for purified wt K29 triUb.

Figure S3

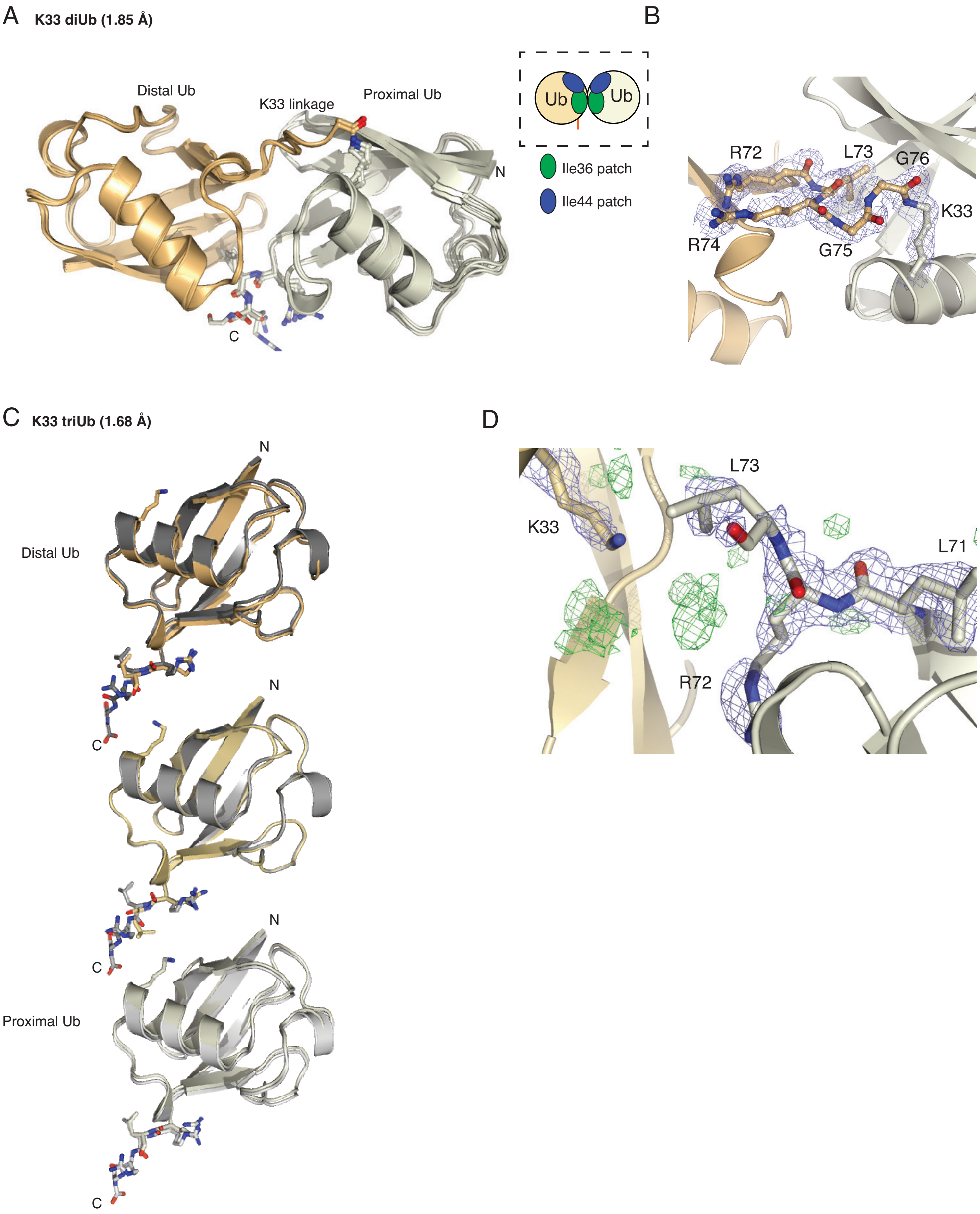

A
K33 diUb (1.85 Å)
Distal Ub
K33 linkage
Proximal Ub
N
C
Ub
Ub
Ile36 patch
Ile44 patch
B
R72
L73
G76
K33
R74
G75
C
K33 triUb (1.68 Å)
N
Distal Ub
C
N
C
N
Proximal Ub
C
D
L73
K33
L71
R72

Figure S3 (related to Figure 3). Structures of K33-linked diUb and triUb. **A)** Crystal structure of K33-linked diUb. The four individual diUb molecules from the asymmetric unit (ASU) are shown superimposed. The isopeptide bond is shown in ball and stick representation, as is the C-terminus of the proximal moiety. (*Insert cartoon*) the Ile36 patch (green) is at the center of the two-fold pseudo-symmetry of the K33 diUb structure and makes the Ile44 patch (blue) appear as a continuous surface across the two Ub moieties. **B)** 2|Fo|-|Fc| electron density, contoured at 1 σ, for the isopeptide linkage in K33-linked diUb. Linkage residues are shown in ball-and-stick representation with red oxygen and blue nitrogen atoms. **C)** Structure of K33-linked triUb. The asymmetric unit contains one Ub and two neighboring ASUs are shown. A K33-linked isopeptide bond can only extend between these symmetry-related molecules. As a comparison, equivalent Ub moieties are shown for monoUb, crystallized in the same space group (1ubq {VijayKumar:1987wy}) and are shown superimposed in shades of grey. Residues corresponding to the Ub C-terminus are shown as sticks for both monoUb and K33 triUb. **D)** Close-up view of the proximal K33 and the C-terminus of the distal Ub moiety. A weighted 2|Fo|-|Fc| electron density map is shown, contoured at 0.8 σ (blue) and a weighted |Fo|-|Fc| difference map showing positive density (green) is shown, contoured at 2.9 σ. Owing to the flexibility of the C-terminus lack of contiguous electron density in this region, no residues were modeled into the density.

Figure S4

A

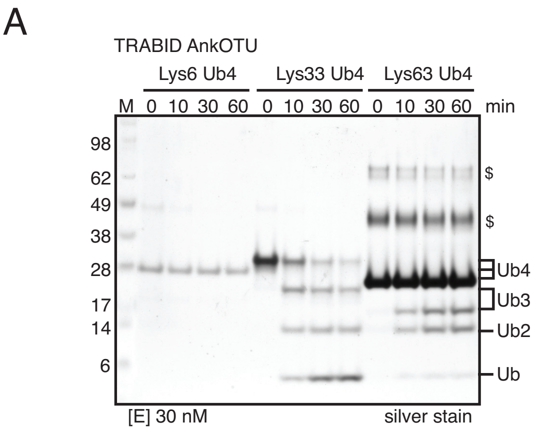

B

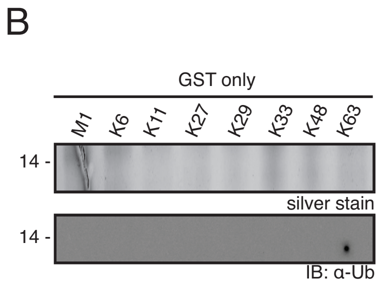

C

	NZF1			
	K_d^{low} (µM)	SEM^{low}	K_d^{high} (µM)	SEM^{high}
monoUb	370	22.9	N.D.	N.D.
M1 diUb	186	5.3	N.D.	N.D.
K6 diUb	295	10.2	N.D.	N.D.
K11 diUb	224	5.6	N.D.	N.D.
K27 diUb	222	7.4	N.D.	N.D.
K29 diUb	201	19.5	3.6	0.3
K33 diUb	179	12.3	4.9	0.2
K48 diUb	339	11.9	N.D.	N.D.
K63 diUb	272	15.9	N.D.	N.D.

D

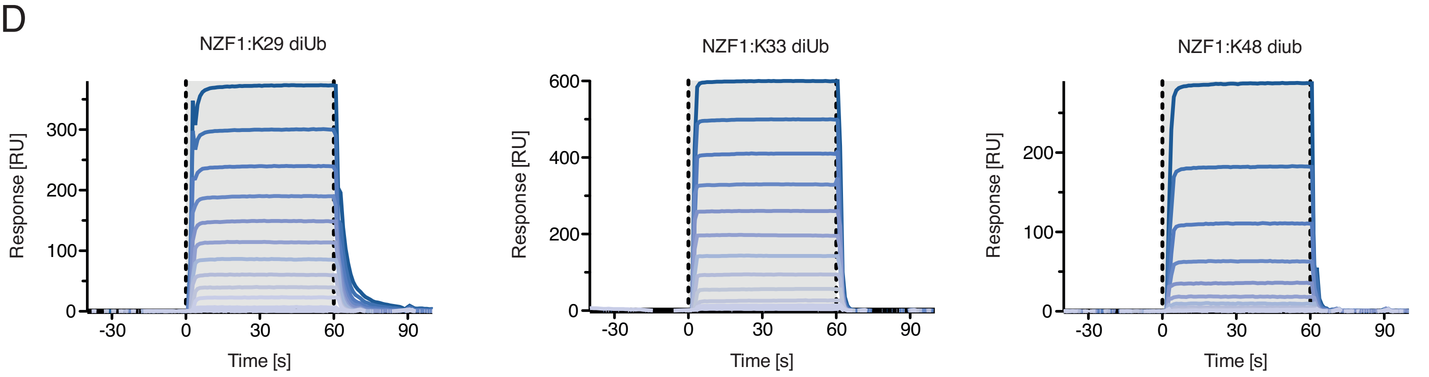

E

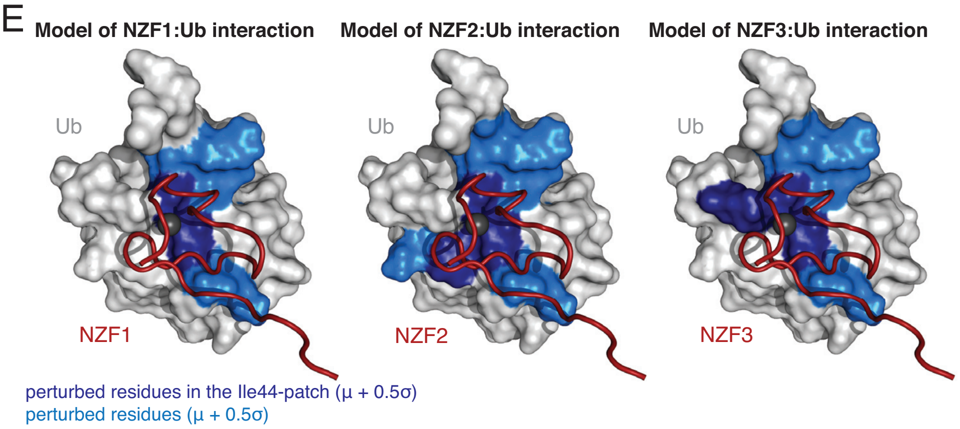

F

	K_d (µM)		
Residue	NZF1	NZF2	NZF3
V5	480	800	440
K6	370	780	370
L8	600	800	270
Q41	420	1000	510
L50	400	1480	1020
V70	430	1410	630
L73	370	830	560
$\langle K_d \rangle$	440	1010	540
STD	30	110	90

G

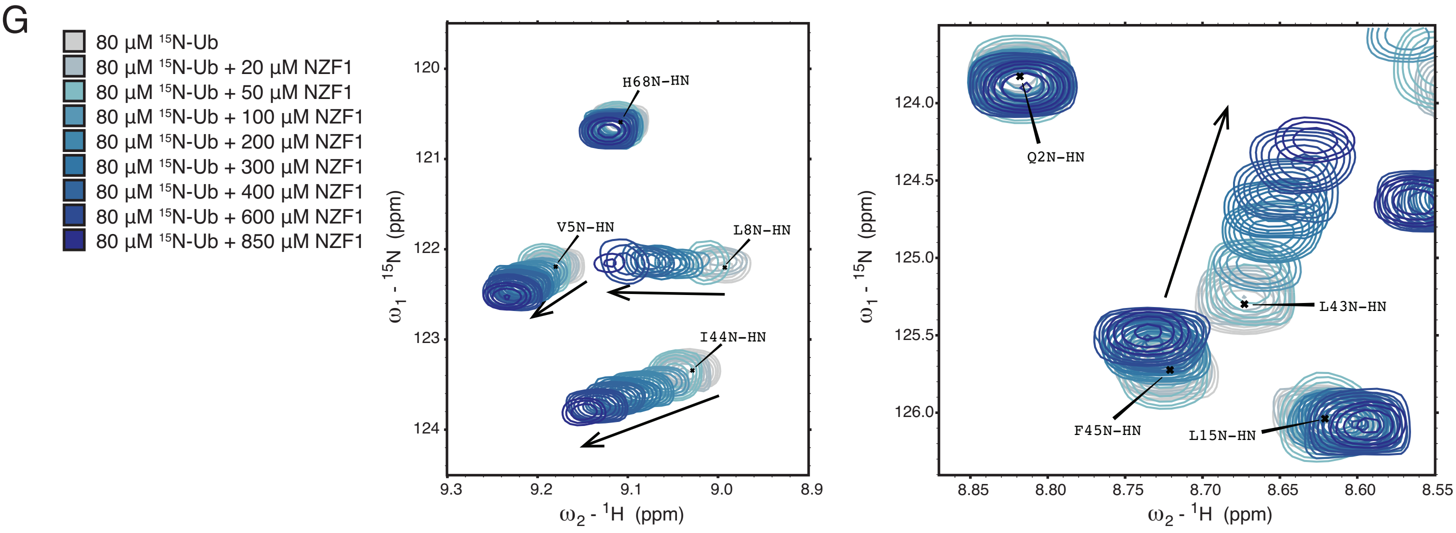

Figure S4 (related to Figure 4). Biochemistry of TRABID Ub interactions. A) DUB assay of TRABID AnkOTU against K6-, K33- and K63-linked tetraUb at 30 nM concentration. **B)** Pull-down of a panel of diUb molecules as in **Figure 4C** with GST as a control. **C)** Standard errors of the mean and best-fit parameters for K_ds of the SPR binding experiments for NZF1 to monoUb and the eight differently linked diUbs are shown. The values are derived from two independent experiments. N.D. indicates that binding was not detectable. K29 and K33 diUb were fitted to a two-site binding model with two K_d values to account for the low-affinity binding to the Ile44 patch of the proximal Ub as well as the higher affinity binding to both the Ile44 patch of the distal Ub and the Glu24 site of the proximal Ub at the same time. **D)** Representative SPR traces for experiments in **Figure 4D** for K29 diUb, K33 diUb and K48 diUb. A gradient from dark to light indicates traces for decreasing concentrations of NZF1. Dotted lines show the start and end of injection. **E)** Structure of Npl4 NZF domain bound to monoUb (pdb-id 1q5w, (Alam et al., 2004)), showing Ub under a surface and the model NZF domain as ribbon to indicate the binding site. Residues with significant chemical shift perturbations (μ+0.5σ) on monoUb upon binding to NZF1, NZF2 or NZF3 of TRABID (*left to right*) were mapped on the surface of Ub, in blue (Ile44, Val70, Leu8) and light blue (others). **F)** Binding constants of individual TRABID NZF domains for monoUb as derived by NMR analysis for individual perturbed resonances as indicated. The average K_d is quoted in the main text. **G)** Titration analysis of NZF domain binding to ^{15}N-labeled Ub by NMR. Data for selected resonances is shown for NZF1.

Figure S5

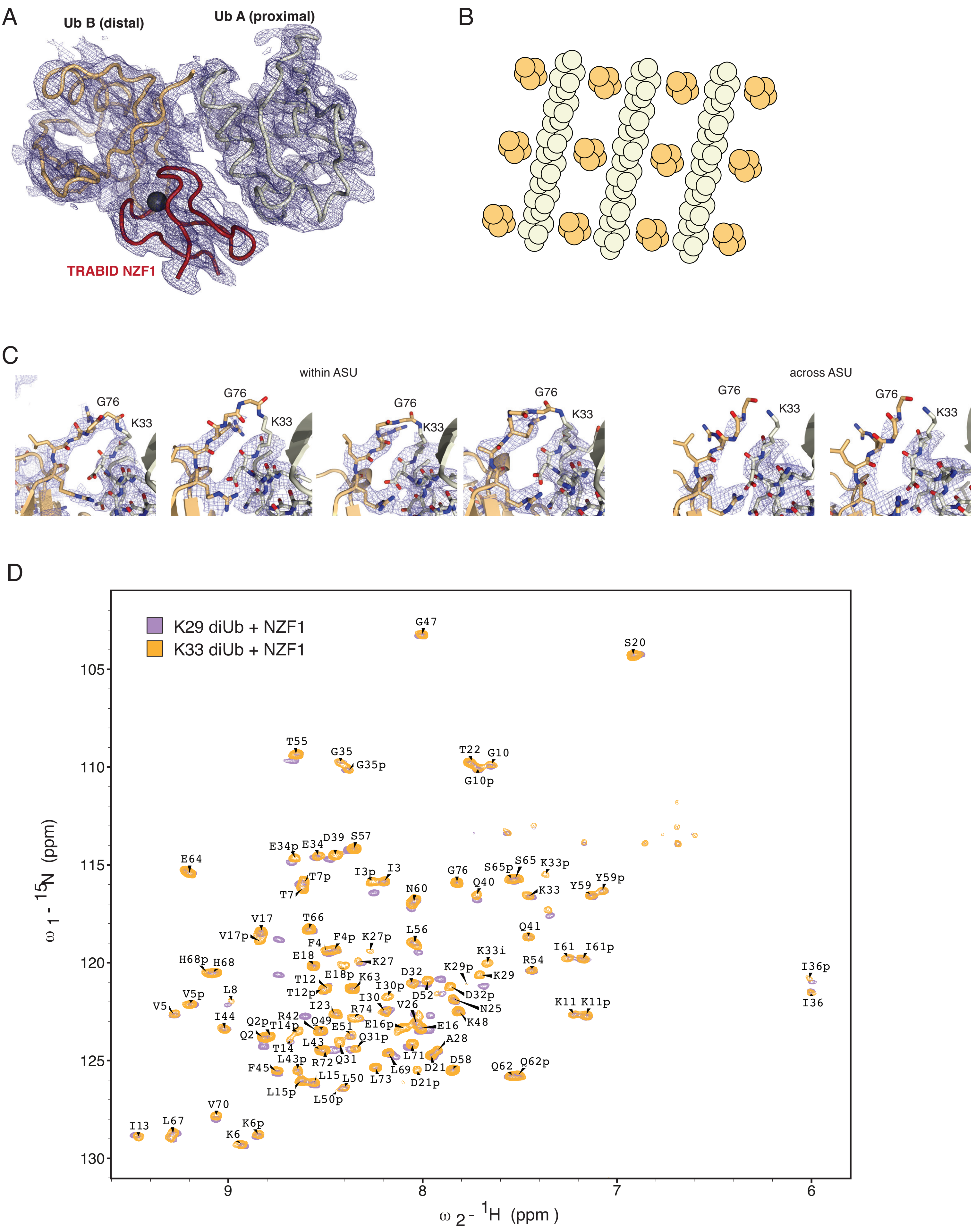
A
Ub B (distal)
Ub A (proximal)
TRABID NZF1
B
C
within ASU
across ASU
G76
K33
D
K29 diUb + NZF1
K33 diUb + NZF1
G47
S20
T55
G35
G35p
T22
G10
G10p
E64
E34p
E34
D39
S57
T7p
T7
I3p
I3
N60
G76
S65p
S65
K33p
Q40
K33
Y59
Y59p
V17
V17p
T66
F4
F4p
K27p
K27
L56
Q41
K33i
I61
I61p
H68p
H68
E18
E18p
T12
T12p
K63
D32
K29p
K29
R54
I36p
I36
V5
V5p
L8
I30p
I30
D52
D32p
N25
I23
R74
V26
K11
K11p
I44
R42
Q2p
T14p
Q49
E51
E16p
E16
K48
Q2
Q31p
T14
L43
A28
L43p
R72
Q31
L71
D58
F45
L69
D21
Q62
Q62p
L15
L50
L73
D21p
L15p
L50p
V70
I13
L67
K6p
K6
105
110
115
120
125
130
9
8
7
6
ω 1 - 15N (ppm)
ω 2 - 1H (ppm)

Figure S5 (related to Figure 5). Structure of TRABID NZF1:K33-linked diUb.

A) Overall view of electron density maps for a K33 diUb:NZF1 complex in the ASU. A 2|Fo|-|Fc| map is shown contoured at 1σ. **B)** Schematic depiction of K33 filaments in the crystal. **C)** 2|Fo|-|Fc| electron density, contoured at 1σ, for residues of the isopeptide bonds between Ub molecules in the ASU (left four panels) and across ASU (right panels). Electron density at the linkage points suffers from low occupancy (0.5) since diUb was crystallized. **D)** BEST-TROSY spectra of ^{15}N-labeled K33 diUb and ^{15}N-labeled K29 diUb each bound to NZF1 at equimolar concentration. Assigned peaks for K33 diUb are labeled. Many peaks overlap suggesting a similar binding mode of NZF1 to K29 and K33 chains.

Figure S6

A

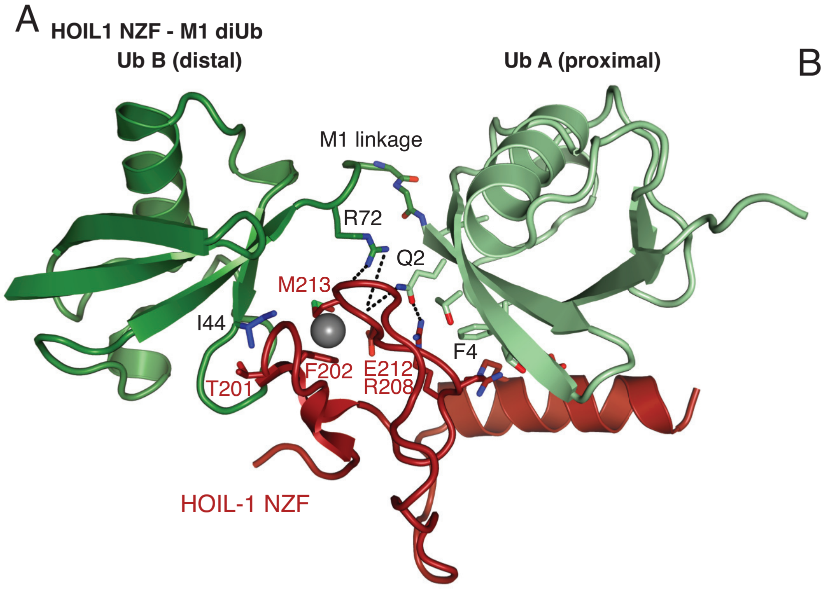

B

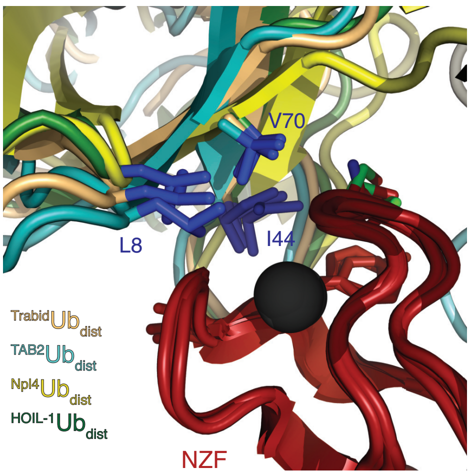

C

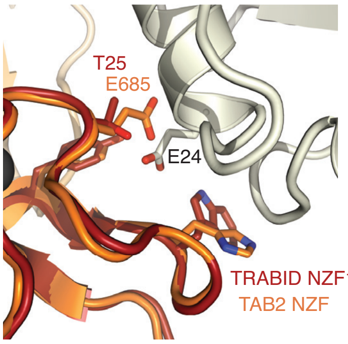

D

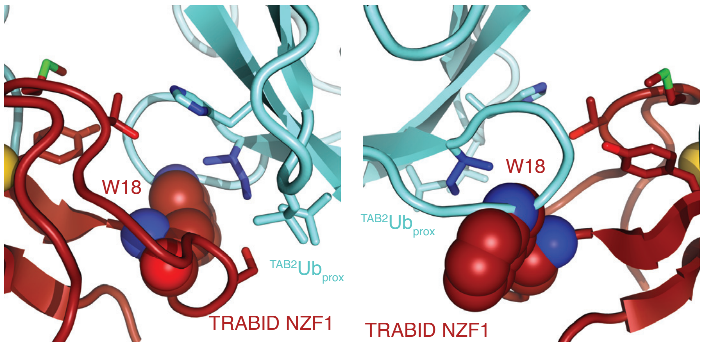

E

```
                                                          10         20         30
NZF1 | H. sapiens / TRABID 5-32               GIKWACEYCTYENWPSAIKCTMCRAQRP

     | H. sapiens / TRABID 85-112             ANKWSCHMCTYLNWPRAIRCTQCLSQRR
NZF2 | X. laevis / Zranb1-A 80-107            STKWSCHMCTYLNWPRAIRCTQCLSQRR
     | D. melanogaster / Trbd 90-117          SEKWPCKVCTYLNWPRSLRCVQCCTKRG

     | H. sapiens / TRABID150-177             TQHWTCSVCTYENWAKAKRCVVCDHPRP
NZF3 | X. laevis / Zranb1-A 145-172           GQHWTCSACTYENCAKAKKCVVCDHPTP
     | D. melanogaster / Trbd 233-260         VSKWACNSCTYENWPRSIKCSMCGKTRE
```

F

		NZF1				
		wt	TY14LV	Y15F	W18A	T25D
K29 diUb	K_d^{low} (μM)	201	N.D.	241	419	229
	SEM^{low}	9.4	N.D.	36.2	20.3	6.1
	K_d^{high} (μM)	3.6	N.D.	10.7	N.D.	N.D.
	SEM^{high}	0.1	N.D.	0.5	N.D.	N.D.
K33 diUb	K_d^{low} (μM)	179	N.D.	251	543	476
	SEM^{low}	5.3	N.D.	28.3	16.2	5.4
	K_d^{high} (μM)	4.9	N.D.	11.4	N.D.	N.D.
	SEM^{high}	0.1	N.D.	0.4	N.D.	N.D.

Figure S6 (related to Figure 6). Specificity of NZF1 for K33-linked chains

A) Structure of HOIL1 NZF domain (red) bound to M1-linked diUb (green). Key residues are depicted in stick representation and hydrogen bonds are indicated as black dashed lines. The proximal Ub interacts *via* its Phe4 patch with a helical extension of the NZF fold. **B)** Structures of NZF domain-Ub complexes (colored as indicated) superimposed on the NZF, which interacts with the Ile44 patch on the distal Ub. **C)** Superposition of TAB2 onto the TRABID NZF1:K33 diUb complex focusing on the proximal Ub binding site. TAB2 Glu685 clashes with Ub Glu24 in the K33 filament. **D)** Superposition of TRABID NZF1 onto the TAB2:K63 diUb complex focusing on the proximal Ub binding site. TRABID Trp18 clashes with the β3-β4 loop of Ub, preventing this Ub orientation. **E)** Alignment of human TRABID NZF1, with TRABID NZF2 and NZF3 from different species. Ser20 in NZF1 is replaced with Arg or Lys NZF2/3, explaining why these NZF domains cannot bind the proximal Ub in an analogous fashion to NZF1. See **Figure 6E** where the NZF1 S20R mutant is tested in pull-down analysis. **F)** A table with best-fit binding parameters from the SPR data for the different NZF1 constructs to K29- and K33-linked diUb are shown. N.D. denotes that no binding could be detected. All data was fitted to a one-site binding model, except for the wt and Y15F constructs that were better described by a two-site binding model (see **Supplementary Experimental Procedures**) and have thus two indicated K_ds.

A

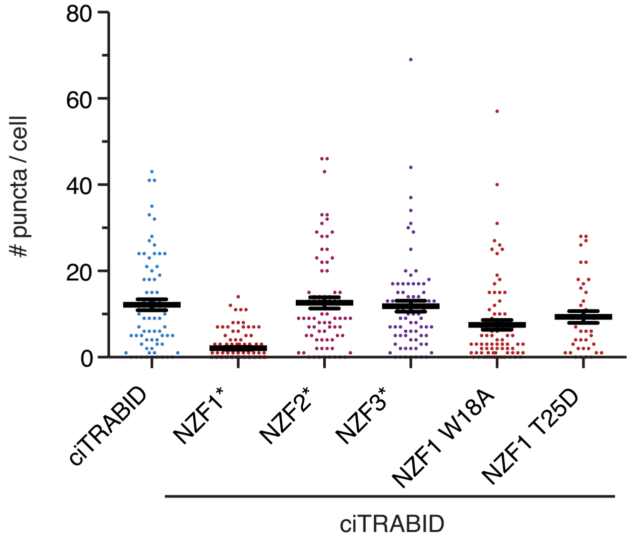

	n	<puncta>	SEM
ciTRABID	76	12.2	1.3
NZF1*	118	2.1	0.3
NZF2*	76	12.6	1.3
NZF3*	78	11.9	1.3
NZF1 W18A	79	7.5	1.1
NZF1 T25D	41	9.8	1.4

B

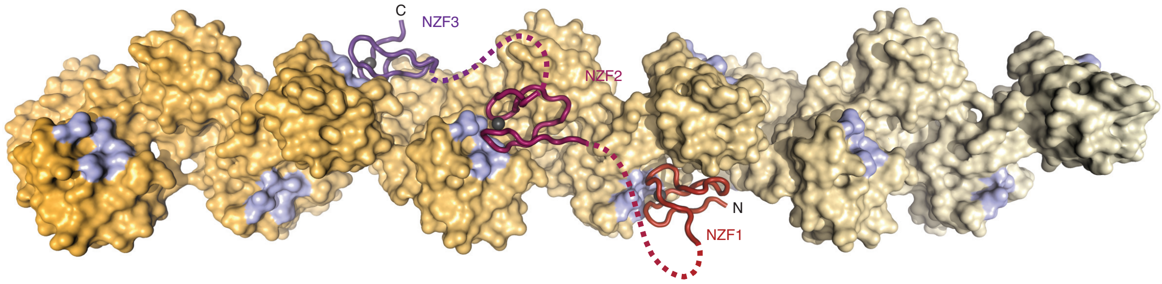

C

```
H. sapiens / TRABID 33-84     NZF1  SGTIITEDPFKSGSSDVGRDWDPSSTEGGSSPLICPDSSARPRVKSSYSM-EN  NZF2
X. laevis / Zranb1-A 33-79          SGAIITEEPFKNSTPDVGSM-----ERDIGSPLICPDSSARPRVKSSYSM-EP
D. rerio / Zranb1-B 33-82           NAPIITEEPFKSSSSLDP---SLCTTQGGSTLLICPDSSARPRVRIADELPET
H. vulgaris / Zranb1 36-70          QN---------------NRWNLVLDETNNDEIINEEIILLS--EKNSANNDK

H. sapiens / TRABID 113-147   NZF2  TRSPTESPQS-----SGSGSRPVAFSVDPCEEYNDRNKLN  NZF3
X. laevis / Zranb1-A 108-142        IRSPTESPQS-----SGSGLRSIPSPIDPCEEYNDRNKLN
D. rerio / Zranb1-B 111-150         QGSQQHSPLSPSETPQTSGSRPSPVTSDPCEEYNDRNRLN
H. vulgaris / Zranb1 109-136        DEFKLVESLKN-----------IENNAECRSDLKERKTI
```

shortest linkers
NZF1-NZF2: 35 aa
NZF2-NZF3: 28 aa

Figure S7 (related to Figure 7 and Discussion). Analysis of TRABID localization in cells and potential TRABID 3xNZF:K33 filament interaction.

A) *(Left)* Graph summarizing localization data for the different mutants. Each point represents the number of puncta of a single cell. Averages and standard errors are indicated in black. *(Right)* A table summarizing the number of analyzed cells as well as the averages and standard deviations for the different mutants. **B)** Model for TRABID 3xNZF interactions with K33 filaments. Three NZF1 domains are depicted, and their respective N- and C-termini are connected by linkers indicated as dashed lines. A direct connection between NZF domains spans 31 Å. **C)** TRABID NZF linkers are not conserved in sequence or length, but have a minimum length of 35 aa (NZF1-NZF2) or 28 aa (NZF2-NZF3) in TRABID from distinct species. Such linkers would span the required distance easily.

Supplementary Experimental Procedures

Molecular Biology

The coding sequences of AREL1 (aa 436-823), UBE3C (693-1083) and TRABID NZF constructs (NZF1: 1-33; NZF2: 84-114; NZF3: 148-178; NZF3+: 147-263; NZF1-3: 1-178; NZF1-3+: 1-263) were amplified using KOD HotStart DNA polymerase (Novagen). AREL1 and UBE3C PCR products were cloned into pOPIN-S, which encodes an N-terminal His6-SUMO tag (Berrow et al., 2007) whilst TRABID NZF constructs were cloned into pOPIN-K, which encodes a 3C cleavable N-terminal His6-GST tag (Berrow et al., 2007) using Infusion HD cloning (Clontech). SMAC (56-239) and HtrA2 (134-458 and 359-458) were amplified with primers that encoded a C-terminal His6-tag and were ligated into an NcoI and HindIII digested pOPIN-K vector. To create the UBE2D2-OTUB1 fusion construct (OTUB1*), overlap extension PCR was used to amplify the full-length (1-147) human UBE2D2 sequence onto the 5' end of human OTUB1 (residues 16-271) with a 10-residue repeating 'Gly-Gly-Ser-Ser' linker sequence. This gene fusion was then inserted into the pOPIN-B vector to create a construct encoding an N-terminal 3C protease-cleavable His6-tag. A similar approach was used to create the STAM2-AMSH fusion (AMSH*), which encodes the VHS and UIM domains of mouse STAM2 (residues 5-188), followed by a 6-residue 'Gly-Gly-Ser-Ser-Gly-Gly' linker and the catalytic domain of human AMSH (residues 243-424). For TRABID localization experiments, TRABID (2-708) was cloned into pEGFP-C1 using XhoI and EcoRI restriction sites to create a TRABID construct with a C-terminal GFP fusion. Mutations were generated by site-directed mutagenesis using the QuickChange method with KOD HotStart DNA polymerase (Novagen). All constructs have been verified by DNA sequencing.

Protein expression and purification

His6-SUMO-AREL1, His6-SUMO-UBE3C, SMAC-His6, HtrA2-His6 and His6-GST-TRABID NZF constructs were expressed in Rosetta2 (DE3) pLacI cells. Cells were grown in 2xTY medium supplemented with 30 μg/ml kanamycin and 35 μg/ml chloramphenicol. The cultures were cooled to 18 °C prior to overnight induction with 400 μM IPTG. For TRABID NZF1, 200 μM of $ZnCl_2$

was added to the culture prior to induction. All proteins were purified by immobilized metal affinity chromatography using either a HisTrap column (GE Life Sciences) or TALON resin (Clontech). AREL1, UBE3C, SMAC-His6 and HtrA2-His6 cell pellets were resuspended in binding buffer (20 mM Tris pH 8.5, 300 mM NaCl, 50 mM imidazole, 2 mM β-mercaptoethanol) and TRABID NZF1 cell pellets were resuspended in binding buffer (50 mM Tris pH 7.4, 150 mM NaCl, 2 mM β-mercaptoethanol). Both cell suspensions were supplemented with lysozyme, DNaseI (Sigma) and protease inhibitor cocktail (Roche) and lysed by sonication. TRABID NZF1 was eluted from Talon resin and further purified using Glutathione Sepharose 4B resin (GE Life Sciences) before the His6-GST tag was cleaved by overnight incubation with 3C protease. Eluted TRABID NZF1 was further purified by size exclusion chromatography (HiLoad 16/60 Superdex 75, GE Life Sciences) in buffer containing: 20 mM Tris pH 7.4, 150 mM NaCl, 2 mM DTT. The resultant fractions were judged to be of sufficient purity following SDS-PAGE analysis and flash frozen. The His6-SUMO tag was cleaved from AREL1 by overnight incubation with SUMO protease at 4 °C. AREL1 was further purified by anion exchange chromatography (ResourceQ, GE Life Sciences) and size exclusion chromatography (HiLoad 16/60 Superdex 75, GE Life Sciences) in buffer containing: 20 mM Tris pH 8.5, 150 mM NaCl, 4 mM DTT. Fractions containing AREL1 were pooled and concentrated to 6 mg/ml and flash frozen. His6-SUMO-UBE3C, SMAC-His6 and HtrA2-His6 variants were eluted from the HisTrap column (GE Life Sciences) and subjected to size exclusion chromatography (HiLoad 16/60 Superdex 75, GE Life Sciences) in the same buffer used for AREL1. Peak fractions were pooled and concentrated prior to being flash frozen. TRABID AnkOTU (245-697) was expressed in ArcticExpress cells (Novagen) and purified according to (Licchesi et al., 2012). OTUB1* and AMSH* were expressed and purified as above, using Talon resin for initial affinity purification, followed by 3C cleavage and final purification using size exclusion chromatography

Ub chain assembly reactions using K-only Ub mutants

Small-scale analysis of HECT E3 ligases with Ub K-only mutants were carried out in 20 µl reactions containing 0.1 µM E1, 2 µM UBE2L3, 11 µM AREL1 or 4

μM GST-NEDD4L (576-955), 50 μM of indicated Ub mutants and 10 mM ATP in ligation buffer (10 mM $MgCl_2$, 40 mM Tris pH 8.5, 100 mM NaCl, 0.6 mM DTT and 10 % (v/v) glycerol). Reactions were incubated at 37 ºC for 60 min prior to being stopped with 4x LDS sample buffer (Invitrogen) and analyzed by SDS-PAGE, using 4-12% NuPAGE gradient gels (Invitrogen). Gels were stained using InstantBlue (Expedeon).

Substrate assembly reactions with AREL1

To investigate whether AREL1 is able to ubiquitinate inhibitors of apoptosis proteins, SMAC and HtrA2 , 150 μl assembly reactions were prepared containing: 0.1 μM E1, 2 μM UBE2L3, 11 μM AREL1, 400 μM Ub in ligation buffer (10 mM $MgCl_2$, 40 mM Tris pH 8.5, 100 mM NaCl, 0.6 mM DTT and 10 % (v/v) glycerol). Reactions contained 50 μM of substrate (SMAC-His6 (52-239), HtrA2-His6 (134-458 and 359-458) and were incubated for 60 min at 37 ºC with/without 10 mM ATP. Reactions were diluted with 500 μl with HisTrap binding buffer (20 mM Tris pH 8.5, 300 mM NaCl, 50 mM imidazole, 2 mM β-mercaptoethanol) and bound to 15 μl Ni-NTA resin (Qiagen). Unbound E2, E3 and Ub were removed through subsequent washes with HisTrap binding buffer before the bound protein was eluted in 2 x 30 μl washes with elution buffer (20 mM Tris pH 8.5, 300 mM NaCl, 500 mM imidazole, 2 mM β-mercaptoethanol). Reactions were analyzed by SDS-PAGE, using 4-12% NuPAGE gradient gels (Invitrogen) and stained using InstantBlue (Expedeon).

K29 chain generation

Large-scale assembly reactions of K29-linked polyUb chains were performed overnight at 37 °C in a reaction volume of 1 ml consisting of 3 mM Ub, 1 μM E1, 10 μM UBE2L3, 32 μM His6-SUMO UBE3C (693-1083), 10 mM ATP, 10 mM $MgCl_2$, 40 mM Tris pH 8.5, 100 mM NaCl, 0.6 mM DTT and 10 % (v/v) glycerol. Following acid precipitation of the reaction enzymes through the addition of perchloric acid (0.25 % final amount), the unanchored Ub chains were buffer exchanged into 50 mM Tris pH 7.4, 150 mM NaCl, 4 mM DTT using a NAP10 desalting column (GE Life Sciences). Linkage-specific deubiquitinating enzymes, OTUB1* (K48-specific) and AMSH* (K63-specific) were added to a final concentration of 1 μM and Cezanne (K11-specific) was

added at 400 nM and incubated for 60 min at 37 °C. Further acid precipitation (0.25 % perchloric acid) removed the DUBs before the sample was buffer exchanged into cation exchange buffer (50 mM NaOAc pH 4.5, 5 % (v/v) glycerol) using a PD10 desalting column (GE Life Sciences). K29-linked Ub chains were resolved by cation exchange chromatography using a MonoS column (GE Life Sciences) and eluted with elution buffer (50 mM NaOAc pH 4.5, 1M NaCl, 5 % (v/v) glycerol). The inclusion of glycerol was important to prevent monoUb inclusion within peak fractions. Peak fractions were dialyzed overnight against 50 mM Tris pH 7.4 prior to concentration (Amicon spin concentrators, 3 kDa molecular weight cut-off).

K33 chain generation

Large-scale assemblies of K33-linked polyUb chains were performed in an analogous way to K29-linked chains. 36 μM AREL1 was used in an assembly reaction in buffer described for K29-chain assembly. The addition of 5 % (v/v) glycerol in the reaction buffer was important to prevent AREL1 precipitation during the reaction. Acid precipitation followed by linkage-specific DUB addition, removed other Ub linkages. Subsequent acid precipitation of the DUBs and cation exchange chromatography allowed the purification of K33-linked chains. Peak fractions were dialyzed overnight against 50 mM Tris pH 7.4 prior to concentration (Amicon spin concentrators, 3 kDa molecular weight cut-off).

Ub chain composition mass spectrometry analysis

Ub chains were separated on a NuPAGE 4-12% gradient gel (Invitrogen) before in-gel digestion with trypsin and the addition of Ub AQUA peptide internal standards according to (Kirkpatrick et al., 2006). Peptides were extracted from gel slices, lyophilized and stored at -80 °C. Tryptic peptides were resuspended in 30 μl of reconstitution buffer (7.5% ACN, 0.5% TFA, 0.01% H_2O_2). Oxidation of peptides containing methionine residues was performed according to (Phu et al., 2010). Two additional 2-fold dilutions for each sample were prepared in order to assess the linearity of detection. 10 μl of each sample was directly injected onto an EASY-Spray reverse-phase column (C18, 3 μm, 100 Å, 75 μm x 15 cm) using a Dionex UltiMate 3000

HPLC system (Thermo Fisher Scientific). Peptides were eluted using a 25 min ACN gradient (2.5-35%) at a flow rate of 1.4 µl min^{-1}. Peptides were analyzed on a Q-Exactive mass spectrometer (Thermo Fisher Scientific) using parallel reaction monitoring (PRM), similar to (Tsuchiya et al., 2013). For PRM assays, monoisotopic precursor masses were isolated (2 *m/z* window) and fragmented at predetermined chromatographic retention times. Precursor masses were fragmented using the following settings: resolution, 17,500; AGC target, 1E5; maximum injection time, 120 ms; normalized collision energy, 28. Raw files were searched and fragment ions quantified using Skyline version 2.5.0.6157© (MacLean et al., 2010). Data generated from Skyline was exported into a Microsoft Excel spread sheet for further analysis according to (Kirkpatrick et al., 2006).

Pull-down assays

Pull-down assays were generally performed as previously (Kulathu et al., 2009). Briefly, 30 µg of GST-tagged NZF domain constructs were incubated with 25 µl of Glutathione Sepharose 4B (GE Life Sciences) for 1 h at 4 °C in 450 µl of pull-down buffer (PDB; 50 mM Tris pH 7.4, 150 mM NaCl, 2 mM β-mercaptoethanol, 0.1 % NP-40). The beads were washed 3 x with PDB and then incubated with 1.5 µg of the indicated diUb in 450 µl PDB plus 0.2 mg/ml BSA overnight at 4 °C. The beads were then washed 5x with PDB prior to SDS-PAGE. Proteins were visualized by silver staining using the Silver Stain Plus Kit (BioRad) according to manufacturers protocols or by Western blotting using a rabbit anti-Ub antibody (Millipore).

Surface plasmon resonance

SPR experiments were performed using a Biacore 2000 with CM5 chips (GE Healthcare), which were functionalized with a 1:1 mix of EDC/NHS. The differently linked diUbs were diluted in 20 mM sodium acetate buffer pH 5.0 to a concentration of 100 ng/µl and centrifuged at 16,000*g* for 10 min to remove aggregates. The diluted protein sample was injected until a response of ~2000 RU was reached. The chip was then blocked using 1 M ethanolamine pH 8.0. One flow channel per chip was functionalized and blocked without the addition of protein to serve as a reference. For affinity measurements, the

samples were buffer exchanged into SPR buffer (20 mM Tris pH 7.4, 150 mM NaCl, 2 mM DTT), and injected for 60 s followed by 150 s dissociation in SPR buffer at 20 °C. The K_d was determined from the data of two experiments using the reference-corrected equilibrium response with the data fitted in Prism 6 (GraphPad Software). Data was fit to a one-site binding model with reasonable absolute sum of squares (ranging from 24-501), except for K29 diUb:NZF1, K29 diUb:NZF1, K33 diUb:NZF1 and K33 diUb:NZF1 Y15F where a two-site binding model was more appropriate. The absolute sum of squares for the four fits dropped from 11938 to 176.5, from 3431 to 338.9, from 22095 to 151.5 and from 8638 to 506.1, respectively, when switching from a one-site to a two-site binding model.

Protein production for NMR analysis

For NMR experiments, cells were grown in 2M9 medium supplemented with ^{15}N-NH_4Cl and/or ^{13}C-glucose. Labeled Ub was purified and assembled into chains as described above. Prior to NMR analysis, all proteins were buffer exchanged against NMR phosphate buffered saline (NMR PBS, 18 mM Na_2HPO_4, 7 mM NaH_2PO_4 pH 7.2, 150 mM NaCl) and 5% D_2O was added as a lock solvent.

NMR analysis

NMR acquisition was carried out at 298 K on a Bruker Avance III 600 MHz spectrometer equipped with a cryogenic triple resonance TCI probe. Topspin (Bruker) and Sparky (Goddard & Kneller, UCSF; http://www.cgl.ucsf.edu/home/sparky/) software packages were used for data processing and analysis, respectively. $^1H,^{15}N$ 2D BEST-TROSY experiments (Favier and Brutscher, 2011) were acquired with in-house optimized Bruker pulse sequences incorporating a recycling delay of 400 ms and 512*64 complex points in the $^1H,^{15}N$ dimension, respectively. High quality 2D data sets were acquired in ~10 min. Weighted chemical shift perturbation calculations were completed using the equation $\sqrt{(\Delta^1H)^2+((\Delta^{15}N)^2/5)}$.
To determine the K_d for each NZF, increasing concentrations of the ligand (20, 50, 100, 200, 300, 400, 600, 850 μM) were titrated into a constant concentration of 80 μM wt ^{15}N-Ub. Changes in either the ^1H or ^{15}N chemical

shift of residues were plotted against the NZF ligand concentration and data fitted using the equation $\Delta\delta_{obs} = \Delta\delta_{max} \{([P]_t+[L]_t+K_d)-[([P]_t+[L]_t+K_d)^2-4[P]_t[L]_t]^{½}\}/2[P]_t$ where $\Delta\delta_{obs}$ is the change in chemical shift from the unbound state, $\Delta\delta_{max}$ is the maximum chemical shift change that occurs at saturation of the Ub and $[P]_t$ and $[L]_t$ are the total protein concentrations of Ub and NZF in each sample (Williamson, 2013).

The backbone assignment of the wt K33 diUb BEST-TROSY was confirmed using an HNCA triple resonance experiment, collected with 1024*32*64 complex points in the ^{1}H, ^{15}N and ^{13}C dimensions respectively. 16 of the 17 additional cross peaks seen in the diUb in comparison to wt Ub were assigned to the proximal molecule with one additional cross peak identified as the amide formed by the isopeptide linkage between the distal Gly76 and the proximal K33 side chain.

NZF1 was added to both the wt K33 diUb sample and the wt K29 diUb sample at a 1:1 concentration ratio. The assignment of the BEST-TROSY spectrum of the K33 diUb with ZNF1 was again confirmed with a triple resonance HNCA experiment collected as above, supplemented with a CBCACONH spectrum collected with 50% Non Uniform Sampling (NUS) and 1024*32*48 complex points in the ^{1}H, ^{15}N and ^{13}C dimensions respectively. The NUS spectrum was processed using Compressed sensing with the MddNMR software package {Kazimierczuk:2011jy}.

Crystallization, data collection and refinement

Crystals of K33-linked diUb were grown by sitting-drop vapor diffusion by mixing an equal volume of K33 diUb (K11R) at 5.5 mg/ml with reservoir (100 mM Tris pH 8.0, 3.0 M $(NH_4)_2SO_4$). Crystals were transferred to a solution containing 3.2 M $(NH_4)_2SO_4$ prior to cryo-cooling. Crystals of K33 triUb were obtained by mixing an equal volume of K33 triUb (K11R) at 3.2 mg/ml with reservoir (10% PEG 20K, 20% PEG 550 MME, 0.02 M D-glucose, 0.02 M D-mannose, 0.02 M D-galactose, 0.02 M L-fructose, 0.02 M D-xylose, 0.02 M N-acetyl-D-glucosamine, 100 mM Tris/Bicine pH 8.5). Crystals grew after 14 days and did not require cryo-protecting prior to vitrification. For crystallization of the TRABID NZF1:K33-linked diUb complex, K33 diUb (assembled from K11R) was mixed with a 1.2 molar excess of TRABID NZF1 (1-33) to a final

complex concentration of 6 mg/ml. Crystals grew at 4 °C from a 1:2 (v/v) ratio of protein to reservoir solution containing 20% PEG 550 MME, 10% PEG 20K, 0.1 M MES/imidazole (pH 6.5), 0.12 M Na oxamate, 0.12 M NaF, 0.12 M Na citrate and 0.12 M Na/K-tartrate (racemic). Crystals appeared after two days and did not require cryo-protecting prior to vitrification.

Diffraction data were collected at Diamond Light Source beam lines I03 for K33-linked diUb and I24 for K33-linked triUb and K33-linked diUb:TRABID NZF1. Diffraction images were integrated using XDS (Kabsch, 2010) and scaled using AIMLESS (Evans and Murshudov, 2013). The structures of K33 diUb and triUb were solved by molecular replacement using Ub (pdb-id 1ubq, {VijayKumar:1987wy}) as an initial search model in PHASER (McCoy et al., 2007). Eight Ub monomers could be placed for the K33 diUb model. However, owing to the size of the asymmetric unit only one Ub monomer could be placed for the K33 triUb model. Iterative rounds of model building and refinement were performed using PHENIX (Adams et al., 2011) and COOT (Emsley et al., 2010), respectively, with TLS restraints used in the latter rounds of refinement. For the K33 diUb model, electron density was of sufficient quality to unambiguously build the isopeptide linkage for three out of the four K33-linked diUbs. However, for the K33 triUb model owing to the isopeptide bond extending across the asymmetric unit (resulting in 2/3 occupancy) and also flexibility of the C-terminal arginyl-glycyl-glycine sequence, then the electron density was not of sufficient quality to confidently build one/two conformations of the Ub C-terminus. The weighted |Fo|-|Fc| had difference density around the ε-amino group of K33, indicating low occupancy isopeptide linkage and no linkage was modeled. TRABID NZF1 K33-linked diUb structure was also solved by molecular replacement using Ub (pdb-id 1ubq) and the TAB2 NZF domain (pdb-id 2wwz, {Kulathu:2009dd}) as search models and refined similarly as the K33 diUb structure. Crystal averaging coupled with flexibility of the C-terminus resulted in weak electron density for the isopeptide bond.

Final statistics can be found in **Table 1**. All structural figures were generated using Pymol (www.pymol.org).

Analysis of TRABID C443S localization

COS-7 cells were grown on coverslips to a confluence of approximately 70%. 24 h after transfection of GFP-ciTRABID constructs (250ng), cells were fixed with 4% paraformaldehyde and permeabilized with 0.1% (v/v) Triton X-100 in PBS and mounted using Vectashield mounting medium with DAPI (Vector Laboratories). Confocal images were taken using the Zeiss LSM 780 microscope. Images of at least 40 cells were analyzed using the Nikon NIS Elements software (Nikon Instruments). The data was plotted in Prism 6 (GraphPad Software) and because the data was not normally distributed, a Mann-Whitney test was performed to determine statistical significance.
