# Supplementary material for: Assembly and Specific Recognition of K29- and K33-Linked Polyubiquitin
Source: Mol Cell. 2015 Apr 2;58(1):95–109. doi: 10.1016/j.molcel.2015.01.042 (PMC4386031; doi:10.1016/j.molcel.2015.01.042)
Supplement: Document S2. Article plus Supplemental Information [file mmc2.pdf]

Molecular Cell

Article

Assembly and Specific Recognition of K29- and K33-Linked Polyubiquitin

Graphical Abstract

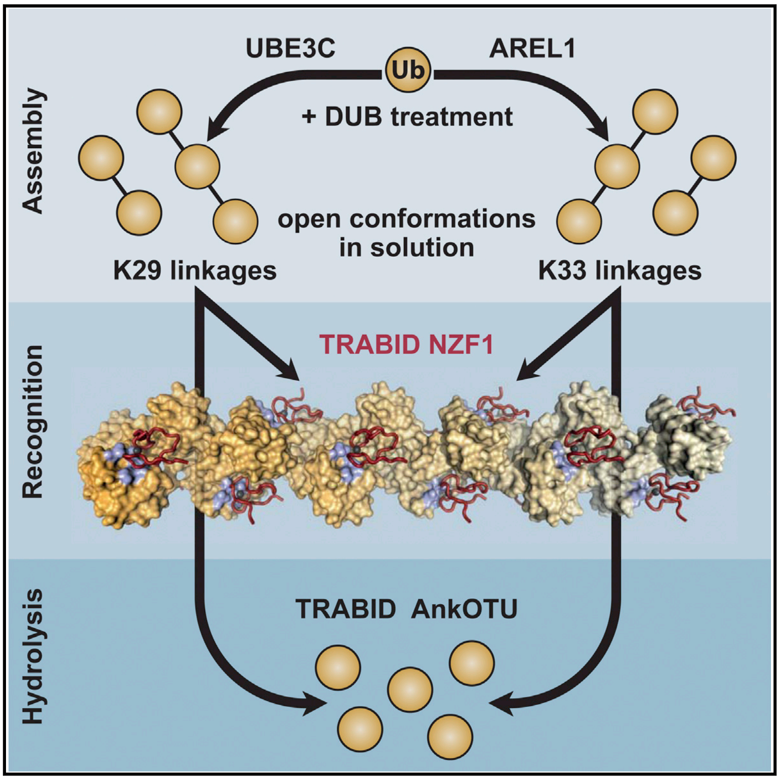

Highlights

- The HECT E3 ligases UBE3C and AREL1 assemble K29- and K33-linked polyubiquitin, respectively
- K29- and K33-linked chains adopt open conformations in solution
- The N-terminal NZF1 domain of TRABID specifically recognizes K29/K33-diubiquitin
- A structure of a K33 filament bound to NZF1 domains explains TRABID specificity

Authors

Martin A. Michel, Paul R. Elliott, ..., Stefan M.V. Freund, David Komander

Correspondence

dk@mrc-lmb.cam.ac.uk

In Brief

Michel et al. reveal that UBE3C and AREL1 assemble K29- and K33-linked polyubiquitin, respectively, on substrates and as unanchored chains. They further identify a K29/K33-specific ubiquitin binding domain in TRABID and structurally characterize how TRABID recognizes K29/K33 filaments.

Accession Numbers

5AF4
5AF5
5AF6

CrossMark
Michel et al., 2015, Molecular Cell *58*, 95–109
April 2, 2015 ©2015 The Authors
http://dx.doi.org/10.1016/j.molcel.2015.01.042

CellPress

Assembly and Specific Recognition of K29- and K33-Linked Polyubiquitin

Martin A. Michel,[1,2] Paul R. Elliott,[1,2] Kirby N. Swatek,[1] Michal Simicek,[1] Jonathan N. Pruneda,[1] Jane L. Wagstaff,[1] Stefan M.V. Freund,[1] and David Komander[1,*]
[1]Medical Research Council Laboratory of Molecular Biology, Francis Crick Avenue, Cambridge CB2 0QH, UK
[2]Co-first author
*Correspondence: dk@mrc-lmb.cam.ac.uk
http://dx.doi.org/10.1016/j.molcel.2015.01.042
This is an open access article under the CC BY license (http://creativecommons.org/licenses/by/4.0/).

SUMMARY

Protein ubiquitination regulates many cellular processes via attachment of structurally and functionally distinct ubiquitin (Ub) chains. Several atypical chain types have remained poorly characterized because the enzymes mediating their assembly and receptors with specific binding properties have been elusive. We found that the human HECT E3 ligases UBE3C and AREL1 assemble K48/K29- and K11/K33-linked Ub chains, respectively, and can be used in combination with DUBs to generate K29- and K33-linked chains for biochemical and structural analyses. Solution studies indicate that both chains adopt open and dynamic conformations. We further show that the N-terminal Npl4-like zinc finger (NZF1) domain of the K29/K33-specific deubiquitinase TRABID specifically binds K29/K33-linked diUb, and a crystal structure of this complex explains TRABID specificity and suggests a model for chain binding by TRABID. Our work uncovers linkage-specific components in the Ub system for atypical K29- and K33-linked Ub chains, providing tools to further understand these unstudied posttranslational modifications.

INTRODUCTION

Protein ubiquitination is an important posttranslational modification that affects virtually every cellular process. Its best-studied function is the degradation of proteins by the proteasome (Hershko and Ciechanover, 1998). However, ubiquitination also regulates alternative degradation pathways, such as ER-associated degradation, autophagy, and mitophagy (Christianson and Ye, 2014; Shaid et al., 2013). In addition, ubiquitination has many non-degradative roles in protein kinase signaling, DNA damage response, intracellular trafficking, transcription, and translation (Komander and Rape, 2012).

During ubiquitination, the 76-amino acid protein ubiquitin (Ub) is attached via its C terminus to, most commonly, Lys residues on substrate proteins. The versatility of Ub in regulating cellular processes arises from its ability to form a wide variety of polyUb chains (Komander and Rape, 2012). Ub has seven internal Lys residues and an N-terminal amino group, all of which can be ubiquitinated, leading to the formation of polyUb chains. Proteomic analyses have revealed that all Ub chain linkages exist simultaneously in cells (Kim et al., 2011; Wagner et al., 2011; Xu et al., 2009). Chains can be homotypic, in which only one linkage type is present, but also heterotypic, in which multiple linkages form mixed and branched structures (Komander and Rape, 2012). Importantly, different linkage types have distinct cellular roles. K48-linked Ub chains are proteasomal degradation signals, whereas K63-linked Ub chains are mainly non-degradative. For the remaining six "atypical" linkage types, cellular roles are less clear. K11-linked chains are important in cell-cycle regulation, where they seem to constitute an alternative proteasomal degradation signal (Wickliffe et al., 2011) but also have other roles (Bremm and Komander, 2011), whereas M1-linked chains have independent non-degradative roles, in particular during NFκB activation and apoptosis (Iwai et al., 2014). For the remaining four chain types, linked via K6, K27, K29, and K33, very little is known, and proteins generating and recognizing these chains in eukaryotic cells are still elusive (Kulathu and Komander, 2012).

The process of ubiquitination is facilitated by an enzymatic cascade in which an E1 Ub-activating enzyme transfers Ub onto the active-site Cys of an E2 Ub-conjugating enzyme (Schulman and Harper, 2009; Ye and Rape, 2009). The E2 enzyme can directly discharge its Ub onto substrates, usually with the help of a RING or U-box E3 ligase (Deshaies and Joazeiro, 2009). Alternatively, a subset of E2 enzymes can perform a transthiolation reaction by transferring Ub onto the active-site Cys of a HECT or RBR E3 ligase. When charged with Ub, HECT and RBR E3 ligases modify substrates in an E2-independent manner (Berndsen and Wolberger, 2014). Importantly, a number of E2 enzymes as well as several HECT and RBR E3 ligases are known to assemble polyUb in a linkage-specific fashion (Kulathu and Komander, 2012; Mattiroli and Sixma, 2014). Based on this knowledge, we have previously described enzymatic assembly systems for K11- and K6-linked chains using a modified E2- and a HECT-like E3 ligase, respectively (Bremm et al., 2010; Hospenthal et al., 2013).

When polyUb chains are generated, they are recognized by Ub binding domains (UBDs), some of which bind polyUb chains in a linkage-specific manner (Husnjak and Dikic, 2012). Linkage-specific UBDs for M1-linked chains have been described (e.g., Sato

CrossMark

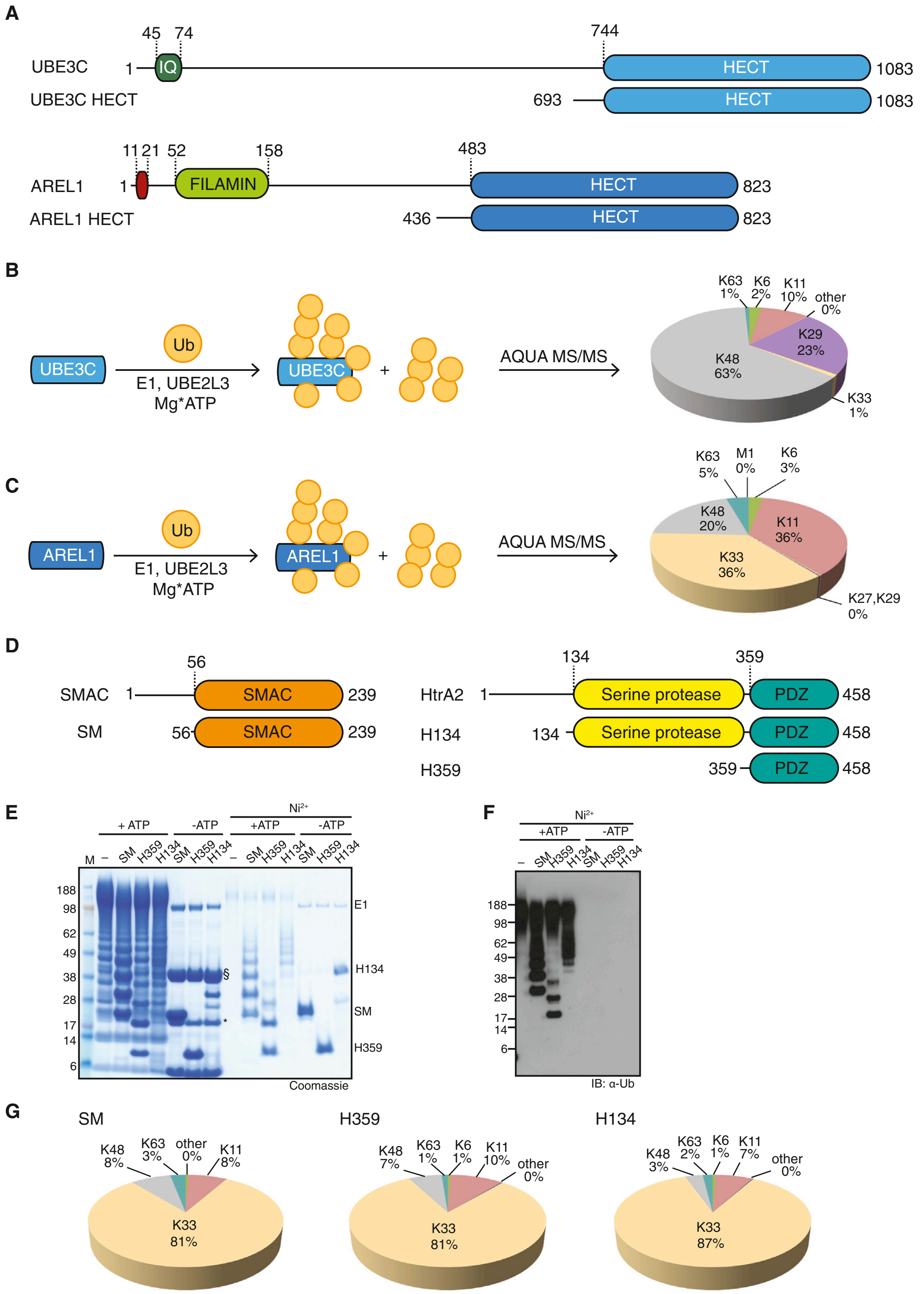

Figure 1. Role of HECT E3 Ligases in Assembling Atypical Ub Chains

(A) Domain structures of UBE3C and AREL1 (KIAA0317) (top) and constructs used in this study (bottom).

(B) Schematic of an assembly reaction with UBE3C, UBE2L3 (UbcH7), E1, and WT Ub (left). The linkage composition in the reaction mixture was analyzed by AQUA-based MS/MS (right).

(legend continued on next page)

et al., 2011) but are unknown for the remaining atypical chain types. Deubiquitinases (DUBs) hydrolyze Ub chains, in some cases with linkage preference (Clague et al., 2013; Komander et al., 2009). Characterization of the ovarian tumor (OTU) DUB family has revealed enzymes to hydrolyze atypical chain types specifically (Keusekotten et al., 2013; Licchesi et al., 2012; Mevissen et al., 2013; Ritorto et al., 2014; Rivkin et al., 2013).

In our search for assembly systems of atypical Ub chain types, we confirmed an earlier report showing that UBE3C primarily assembles K29- and K48-linked chains (You and Pickart, 2001) and further discovered that the HECT E3 ligase apoptosis-resistant E3 ubiquitin protein ligase 1 (AREL1), also known as KIAA0317 (Kim et al., 2013), assembles atypical K11- and K33-linked chains in autoubiquitination reactions and predominantly K33-linkages in free chains and on reported substrates. Treatment of assembly reactions with linkage-specific DUBs enabled purification of K29- and K33-linked polyUb in quantities suitable for biophysical and structural studies. This enabled the structural characterization of the polymers and of the K29/K33 linkage-specific OTU family DUB TRABID (Licchesi et al., 2012). DiUb of both linkage types adopt open conformations in solution, similar to K63-linked polyUb. We found that the TRABID N terminus, which contains three Npl4-type zinc finger (NZF) UBDs, specifically binds K29- and K33-linked diUb, and specificity can be attributed to the first NZF domain (NZF1). A crystal structure of NZF1 bound to K33-linked diUb reveals an intriguing filamentous structure for K33 polymers in which NZF1 binds each Ub-Ub interface. A similar binding mode is observed for K29-linkages in solution studies, together suggesting a model for TRABID interaction with atypical chains. Inactive TRABID localizes to Ub-rich puncta in cells, and this is attenuated when the K29/K33-specific binding mode is disrupted by point mutations. Our work unlocks K29- and K33-linked Ub chains for biochemical studies and provides a launching point for future discoveries related to these atypical Ub signals.

RESULTS

HECT E3 Ligases Assemble Atypical Ub Chains

The HECT family of E3 ligases contains 28 members, many of which have important cellular functions (Rotin and Kumar, 2009). Much research has focused on the first discovered member, E6AP (Scheffner et al., 1993), and on the NEDD4 family, which comprises Rsp5 in yeast and nine enzymes in humans (Rotin and Kumar, 2009). Interestingly, although E6AP assembles K48-linked chains, the NEDD4 family specifically assembles K63 linkages in vitro. Elegant biochemical and structural work has identified many features important for linkage specificity (Kamadurai et al., 2009, 2013; Kim and Huibregtse, 2009; Maspero et al., 2013). Because of their ability to dictate linkage specificity and many hints in the literature (Tran et al., 2013; You and Pickart, 2001), we characterized human HECT E3 ligases to investigate their ability to assemble atypical chains.

One way to assess which linkage types are assembled is to utilize a panel of Ub mutants in which each Lys is mutated to Arg either inclusively (K0) or with the exception of one position (Kx-only) (Figure S1A). This analysis reproduced K63 specificity of NEDD4L (amino acids [aa] 576–955) (Figure S1B) and indicated a broader specificity of the unstudied HECT E3 ligase AREL1 (aa 436–823, Figure 1A; Figure S1C), which seemed to assemble K33 linkages efficiently.

Using Ub mutants for chain assembly has multiple caveats. To understand which linkage types are assembled in E3 ligase reactions with wild-type (WT) Ub, we used absolute quantification (AQUA)-based mass spectrometry (Kirkpatrick et al., 2006). For this, tryptic digests of chain assembly reactions are spiked with isotope-labeled GlyGly-modified standard peptides derived from each potential linkage site, allowing absolute quantification of all chain types (Kirkpatrick et al., 2006). NEDD4L assembled K63 chains almost exclusively (96%) (Figure S1D), whereas UBE3C assembled K48 (63%), K29 (23%), and K11 linkages (10%) (Figure 1B), as reported previously (Maspero et al., 2013; You and Pickart, 2001). Interestingly, AREL1 assembled 36% of K33, 36% of K11, 20% of K48, and small amounts of K63 and K6 linkages (Figure 1C). The high abundance of K11 linkages in AREL1 reactions contrasts with the finding from K11-only Ub that was incorporated poorly into chains (Figure S1C), suggesting that mutated Lys residues are crucial for assembly of this linkage type by AREL1. Abrogating K11 linkage production by AREL1 using Ub K11R led to 71% of K33 and 24% of K48 linkages (Figure S1E). The fact that K48 linkages stayed relatively constant indicated that this chain type is assembled as a constant byproduct of AREL1.

A recent characterization of AREL1 function (Kim et al., 2013) has suggested that the pro-apoptotic proteins SMAC, HtrA2, and ARTS are among its substrates and further indicated that they interact via the AREL1 HECT domain (rather than an auxiliary N-terminal domain). We expressed fragments of SMAC and HtrA2 (Figure 1D) and used these proteins as in vitro AREL1 substrates. AREL1 ubiquitinated all proteins efficiently (Figures 1E and 1F). Strikingly, AQUA analysis of modified substrates showed that AREL1 had assembled >80% of K33 linkages in the polyUb chains on all three substrates (Figure 1G).

Generating K29- and K33-Linked PolyUb

AREL1 and UBE3C also assembled free Ub chains. Precipitation of enzymes by perchloric acid in an assembly reaction enriched free polyUb chains of varying lengths (see gel in Figure 2A). AREL1 assembled WT Ub into di- and triUb with (for triUb) 75% of K33-linkages and only 13% of K11-linked and 7% of K63-linked chains (Figure 2A). Using K11R Ub, we generated up to 86% of K33 linkages in triUb (Figure 2A). To generate

(C) Reaction as in (B) with AREL1, UBE2L3, E1, and WT Ub.

(D) Domain structures of the pro-apoptotic proteins SMAC and HtrA2 (top) and the expressed constructs used in this work (bottom).

(E) AREL1 is able to assemble chains onto SMAC and HtrA2 in an in vitro ubiquitination reaction that depends on ATP. Ubiquitinated, His6-tagged substrates are enriched following Ni^{2+} affinity binding. SM, SMAC (56–239); H359, HtrA2 (359–458); H134, HtrA2 (134–458); §, AREL1; *, UBE2L3.

(F) Western blot against Ub of the Ni^{2+}-enriched reaction from (E).

(G) AQUA MS/MS profiles of the ubiquitinated substrates purified from (E).

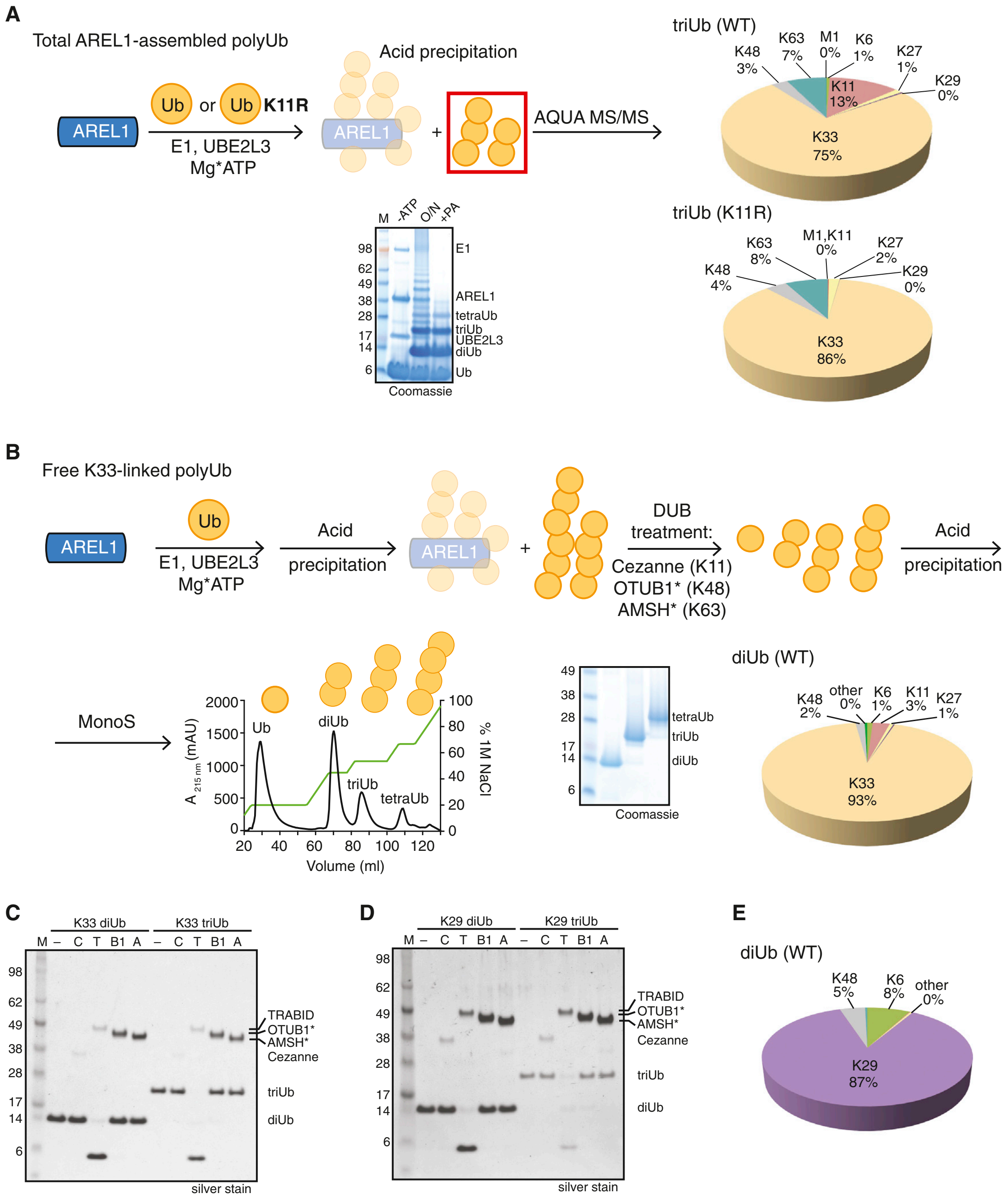

Figure 2. Purification of Unanchored K29/K33 PolyUb Chains

(A) Schematic of the assembly of K33-linked Ub chains using either WT or K11R Ub (top). AQUA profiles of triUb using either WT (top right) or K11R Ub (bottom right; K6 linkage was excluded from the quantitative analysis because of the K11R substitution). Bottom: corresponding SDS-PAGE gel for assembly of free chains. −ATP, initial reaction without ATP addition; O/N, overnight incubation of the assembly reaction; +PA, perchloric acid treatment of the assembly reaction.

(B) Schematic representation of the purification of K33-linked polyUb chains. Following the assembly reaction, perchloric acid treatment removes the ubiquitinated and unmodified forms of E1, E2, and E3. Linkage-selective DUBs are then used to remove undesired Ub linkages. An additional perchloric acid step is required to inactivate the DUBs prior to cation exchange chromatography (bottom), which resolves the homotypic chains based on linkage length. Bottom center: SDS-PAGE of purified K33-linked di-, tri-, and tetraUb. Bottom right: AQUA MS/MS of purified K33-linked diUb.

(C) Deubiquitinase assay of purified K33-linked di- and triUb. –, no DUB; C, 200 nM Cezanne (K11-specific); T, 350 nM TRABID (K29/K33-specific); B1, 1 μM OTUB1* (K48-specific); A, 1 μM AMSH* (K63-specific).

(D) K29-linked polyUb chains can be purified analogous to the schematic shown in (B). Purified K29-linked di- and triUb were treated with DUBs as in (C).

(E) AQUA mass spectrometry profile of purified K29 diUb.

Table 1. Data Collection Statistics

	K33 diUb	K33 triUb	TRABID NZF1-K33 diUb
Data Collection			
Beamline	Diamond I03	Diamond I24	Diamond I24
Space group	*I* 4	*P* 2_1 2_1 2_1	*C* 2
a, *b*, *c* (Å)	113.08, 113.08, 103.90	28.42, 42.48, 50.52	98.38, 126.51, 78.09
α, β, γ (°)	90.00, 90.00, 90.00	90.00, 90.00, 90.00	90.00, 103.38, 90.00
Wavelength	0.9763	0.9686	0.9686
Resolution (Å)	45.47–1.85 (1.89–1.85)[a]	23.62–1.68 (1.72–1.68)	38.59–3.40 (3.67–3.40)
R_{merge}	4.5 (44.3)	5.1 (77.6)	10.9 (56.2)
$I/\sigma I$	11.9 (2.5)	19.9 (2.7)	6.6 (2.0)
Completeness (%)	99.8 (100)	99.8 (99.5)	99.9 (100)
Redundancy	3.5 (3.5)	7.3 (7.7)	3.4 (3.4)
Refinement			
Resolution (Å)	45.47–1.85	23.62–1.68	38.59–3.40
No. of reflections	55,562	7,363	12,783
R_{work}/R_{free}	22.9/27.1	19.4/22.5	18.0/22.2
No. of Atoms			
Protein	4,788	596	4,076
Ligand/ion	84		5
Water	128	49	
B Factors			
Wilson *B*	33.77	26.49	83.17
Protein	70.20	35.00	106.68
Ligand/ion	67.50		83.84
Water	45.90	43.17	
RMSDs			
Bond lengths (Å)	0.005	0.002	0.002
Bond angles (°)	0.930	0.748	0.603
Ramachandran statistics (favored /allowed/outliers)	99.0/1.0/0.0	100.0/0.0/0.0	98.8/1.2/0.0

[a]Numbers in brackets are for the highest-resolution bin.

pure K29 and K33 chains from WT Ub, we acid-precipitated the reaction and treated the free chains with a panel of linkage-specific DUBs consisting of K11-specific Cezanne (Mevissen et al., 2013) as well as enhanced versions of K48-specific OTUB1* (an UBE2D2-OTUB1 fusion) and K63-specific AMSH* (a STAM2-AMSH fusion) (Figure 2B; Figures S2A and S2B; Supplemental Experimental Procedures). The resulting K29- and K33-linked polyUb chains were purified by cation exchange and were 87% and 93% pure, respectively; uncleavable by Cezanne, OTUB1*, or AMSH*; but hydrolyzed efficiently by the K29/K33-specific DUB TRABID (Licchesi et al., 2012) (Figures 2C–2E; Figures S2C and S2D; also see below).

K29- and K33-Linked diUbs Adopt Open Conformations in Solution

With new linkage types at hand, we set out to understand their structural features. We crystallized K33-linked di- and triUbs (Figure S3). The K33-linked diUb crystallized in space group *I*4, not observed previously for Ub, and a structure to 1.85 Å resolution revealed eight molecules forming four identical Ub dimers with clear electron density for the K33 linkages (Figures S3A and S3B; Table 1). In this structure, K33-linked diUb is compact, and distal and proximal Ub moieties interact symmetrically via their Ile36 hydrophobic patches (Figure S3A). The symmetric interface did not provide a model for the conformation of a longer K33 chain.

A second crystal structure for K33-linked triUb was obtained at 1.68 Å in space group $P2_12_12_1$ with similar unit cell dimensions as the Ub reference structure (1ubq; Vijay-Kumar et al., 1987), and also contains only one Ub molecule per asymmetric unit (Table 1). Examination of adjacent asymmetric units only allowed one possibility for K33 chain formation (Figure S3C), although the C termini and isopeptide linkages were poorly ordered and not built in the model (Figure S3D). The Ub moieties in K33 chains were related by translational symmetry and, in contrast to the compact diUb structure, adopted an open conformation in which Ub moieties do not interact with each other except by two polar side chain contacts (Figure S3C). The distinct conformations of di- and triUb could be due to differences in crystallization conditions but highlight the underlying problem that crystallization

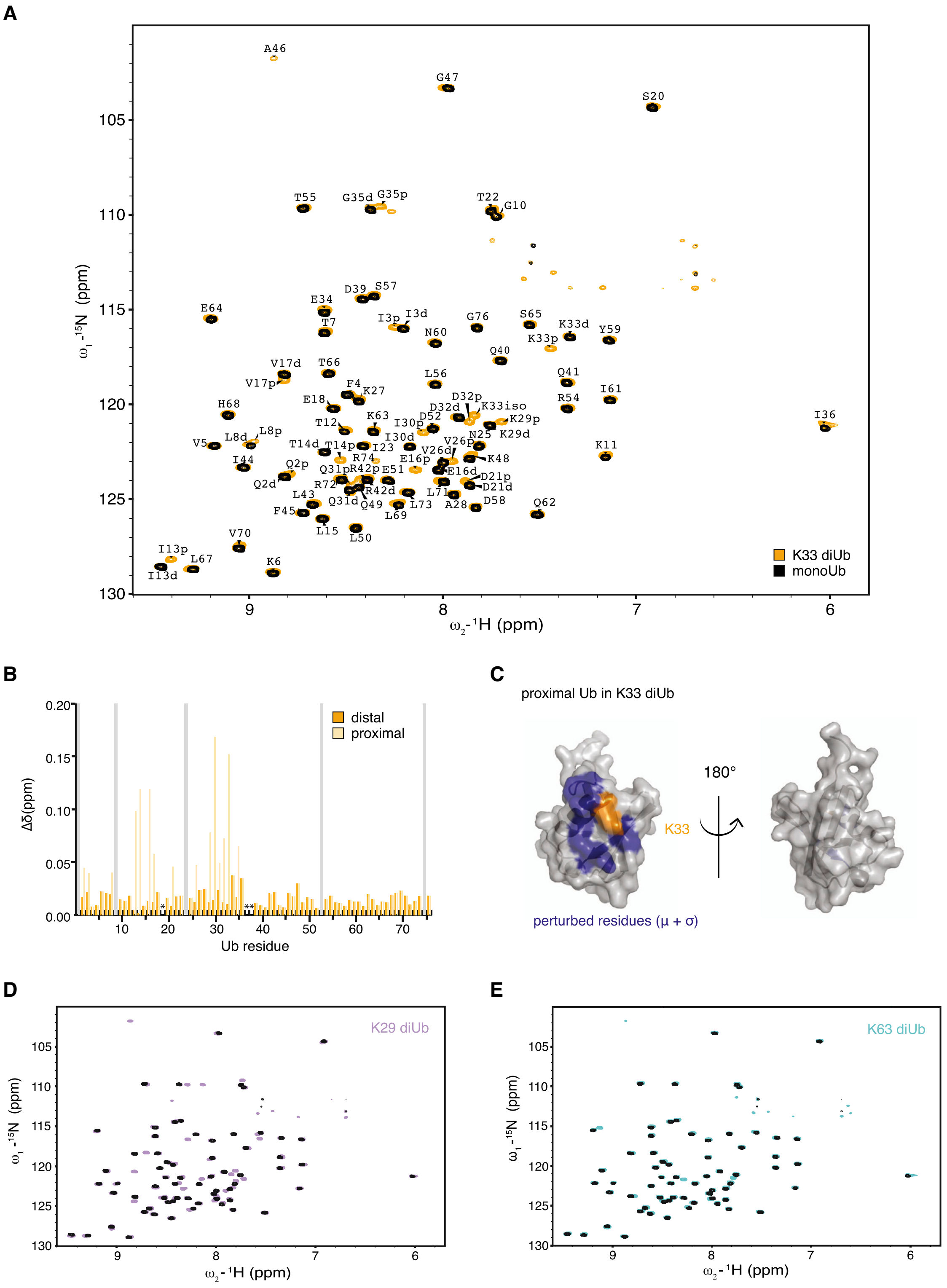

Figure 3. NMR Analysis of K29/K33 Chains

(A) BEST-TROSY spectra for ^{15}N-K33 diUb (orange) and ^{15}N-monoUb (black). Complete assignment of resonances from the proximal (p) or distal (d) moieties from a ^{13}C, ^{15}N-K33 diUb sample are shown.

(B) Chemical shift perturbation of distal (orange) and proximal (beige) resonances with respect to monoUb. Grey bars, exchange-broadened resonances; asterisks, proline residues.

(legend continued on next page)

may present an incomplete picture of the dynamic states of free polyUb in solution.

We therefore turned to nuclear magnetic resonance (NMR), which is better suited to analyze dynamic Ub chains. When Ub polymers adopt open conformations in solution, the spectra resemble free monoUb, showing a small number of perturbations surrounding the isopeptide linkage. The best example for this is K63-linked Ub (Varadan et al., 2004) which does not form a defined interface in solution (Ye et al., 2012). Contrasting this are compact conformations in which defined interfaces are formed. In K48-linked diUb (Varadan et al., 2002), resonances of proximal and distal Ub moieties adopt distinctly different chemical shift positions (splitting) because of their different chemical environment.

We assembled K33-linked diUb from ^{13}C, ^{15}N-labeled monoUb and measured 2D band-selective excitation short transient transverse relaxation-optimized spectroscopy (BEST-TROSY) spectra, revealing well dispersed peaks similar to monoUb (Figure 3A). Assignment of the spectra revealed splitting of 16 resonances. The small chemical shift perturbations (CSPs) in all split resonances could be attributed to the proximal Ub, whereas the distal Ub was unperturbed (Figure 3B). Mapping of the perturbed residues on Ub revealed a small region surrounding the isopeptide bond at K33 (Figure 3C). Almost identical spectra were obtained for ^{15}N-labeled, K29-linked diUb, in which 19 resonances were split and mildly perturbed (Figure 3D). Both diUb spectra resembled the K63 diUb spectrum (Varadan et al., 2004; Figure 3E). Together, this indicates that both K29- and K33-linked diUb do not form defined interfaces in solution but, rather, exist in open conformations.

TRABID K29/K33 DUB Specificity Is Retained with Longer Chains

TRABID, a DUB from the OTU family, is the only known protein to date that acts specifically on K29- and K33-linked Ub chains (Licchesi et al., 2012; Mevissen et al., 2013; Figure 4A). In TRABID, a C-terminal OTU domain of the A20 subfamily is preceded by an ankyrin repeat Ub binding domain (AnkUBD) that enables a non-specific OTU domain to preferentially cleave K29- and K33-linked diUb (Licchesi et al., 2012). This construct hydrolyzes K63-linked chains with 40-fold lower activity compared to K29 chains (Virdee et al., 2010). Because of the necessity for chemical synthesis of K27, K29, and K33 linkages, these experiments were so far confined to diUb cleavage. We now confirm that the specificity of TRABID AnkOTU also holds true for longer tetraUb chains. TRABID cleaved K33-linked tetraUb with a significantly higher activity compared with K63-linked chains (Figures 2C, 2D, and 4B; Figure S4A).

TRABID NZF1 Specifically Binds K29- and K33-Linked Chains

In addition to the C-terminal AnkOTU catalytic domain, TRABID contains three N-terminal NZF domains (Figure 4A) that bind polyUb and are important, together with the AnkUBD, for TRABID localization to characteristic punctate structures in cells (Licchesi et al., 2012). Surprisingly, analyzing the preference of the N-terminal NZF domains in pull-down experiments revealed the specificity of the 3xNZF module (aa 1–263 or 1–178) for K29- and K33-linked diUb, whereas K63-diUb binding was barely detectable (Figure 4C; Figure S4B). This resembled the cleavage specificity of the AnkOTU catalytic domain (Figure 4B; Licchesi et al., 2012). Pull-down experiments with individual NZF domains showed that K29/K33 specificity could be attributed entirely to the N-terminal NZF1 domain (aa 1–33), which bound these chains as well as the 3xNZF modules but did not interact with K63-linked diUb.

To measure affinities, we established a surface plasmon resonance (SPR)-based binding assay in which monoUb and all types of diUb were immobilized, and NZF1 binding was detected by SPR (Figure 4D). Of the nine datasets, seven were fitted to a one-site binding model with NZF1 affinities between 190–370 μM (Figures S4C and S4D). For K29- and K33-linked diUb, fitting to a one-site model resulted in high residuals, and data were fitted to a two-site binding model, revealing significantly higher affinities (K_d^{high} 3.6 and 4.9 μM, respectively, and 180/200 μM affinities for K_d^{low}) (Figure S4C). The high binding affinities of K29- and K33-linked diUb were consistent with the pull-down experiments (Figure 4C).

Curiously, NZF2 and NZF3 did not bind diUb in pull-down assays. To understand whether these domains can bind Ub, binding studies were performed by NMR using ^{15}N-labeled Ub. NZF1 interacts with monoUb, leading to chemical shift perturbation maps that show the characteristic profile for interactions via the Ub Ile44 patch (Figure 4E; Figure S4E). This is consistent with the known binding mode of NZF domains first derived for Npl4 (Alam et al., 2004), and mapping of perturbed residues on Ub suggests similar interactions (Figure S4E). Titrations of NZF2 and NZF3 resulted in similar CSP profiles (Figure 4F; Figure S4E). Using NMR titration experiments, we derived binding constants for the monoUb-NZF interactions, with K_d values of $\sim$440 μM for NZF1, $\sim$1 mM for NZF2, and $\sim$540 μM for NZF3 (Figures S4F and S4G), which is in a typical range for monoUb binding to small UBDs. Although this showed that all NZF domains are functional in Ub binding, it did not explain why only NZF1 showed K29/K33 specificity.

Structure of a K33-Linked Ub Polymer Bound to TRABID NZF1

To understand the underlying molecular basis for the K29/K33 specificity of TRABID NZF1, we crystallized the complex with K33-diUb and determined a structure to 3.4 Å resolution. High solvent content (67%, Matthews coefficient $\sim$ 3.8) led to high-contrast maps (Figure S5A) and allowed building of a complete model with good statistics (Table 1). It was immediately apparent that the arrangement of Ub molecules in the crystal lattice generated seemingly infinite helical polymers (Figures 5A and 5B; Figure S5B). The K33-filament forms a helix with 5-fold

(C) Resonances that display a perturbation of more than 1 σ are mapped onto the surface of monoUb (purple) and cluster around the K33 residue (orange). No significant perturbations were observed on the distal Ub moiety, consistent with an open conformation of K33 diUb.
(D) BEST-TROSY spectra for ^{15}N K29 diUb (purple) and monoUb (black).
(E) BEST-TROSY spectra for ^{15}N K63 diUb (cyan) and monoUb (black).

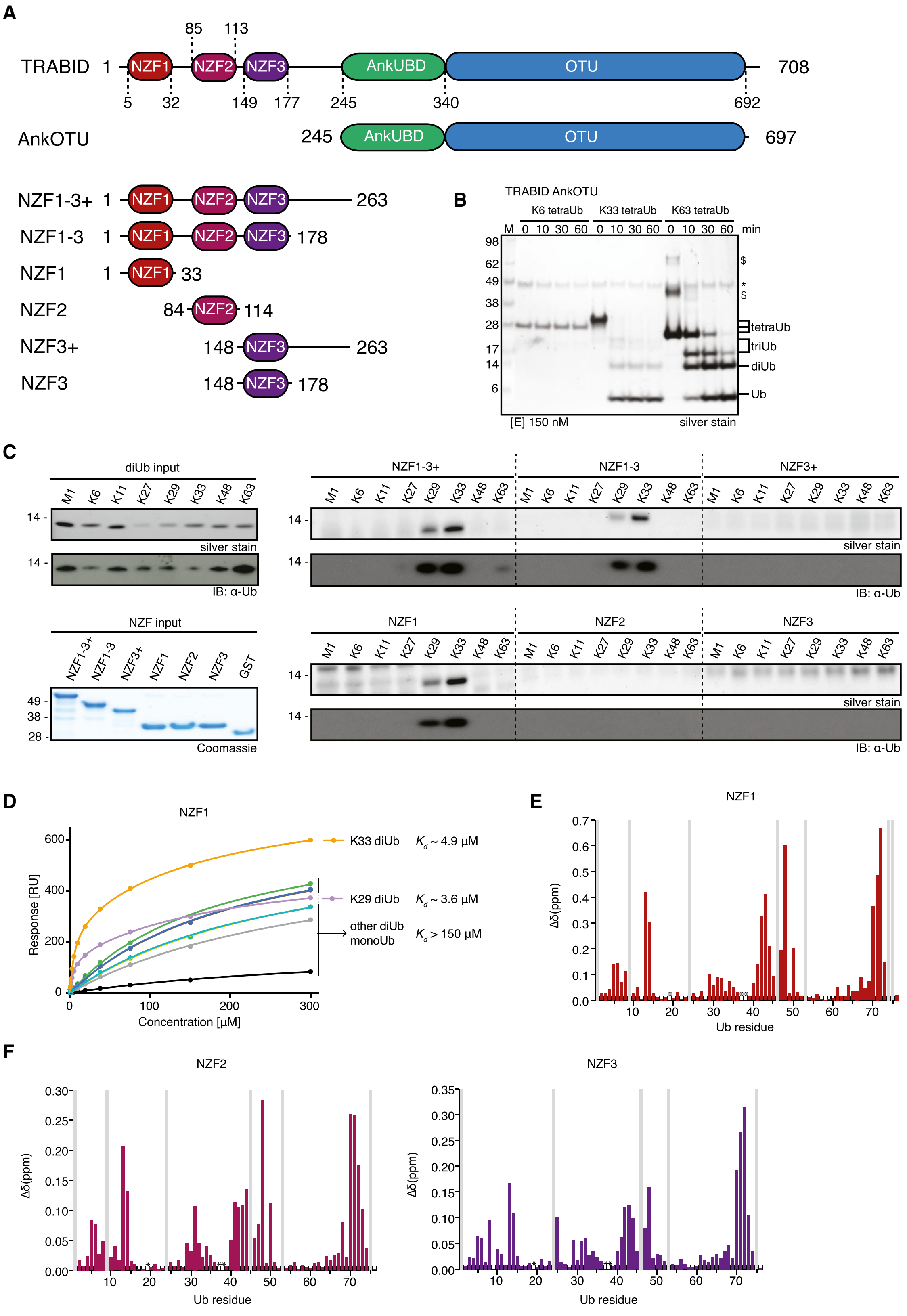

(legend on next page)

CellPress

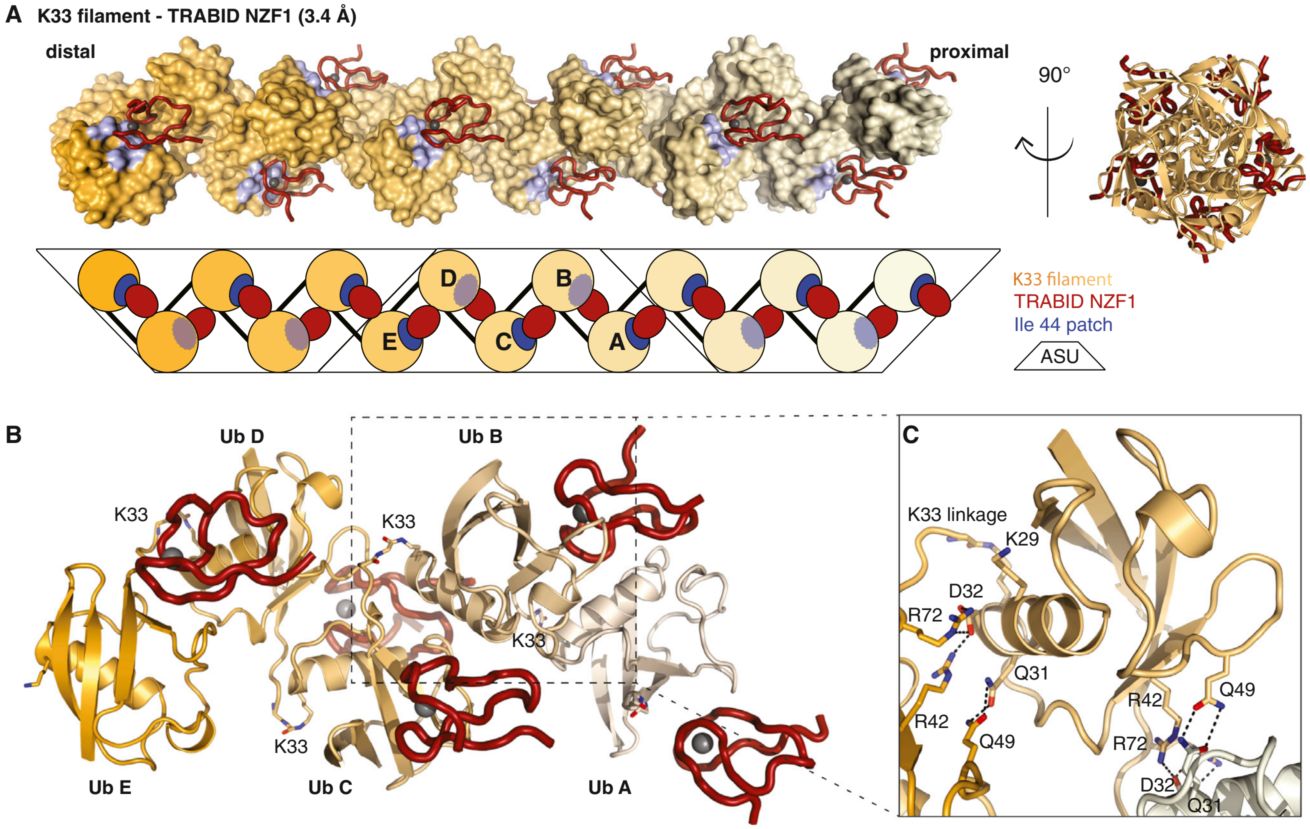

Figure 5. Structure of K33 Filaments Bound to NZF1

(A) Structure of the K33-linked Ub filament as observed in the crystal, showing three adjacent asymmetric units (black outline). Ub molecules are shown as a surface representation with a gradient from orange (distal) to beige (proximal), and Ile44 patches are indicated in blue. NZF1 is shown as a red ribbon with gray Zn^{2+} atoms. A schematic is shown below. Right: view of the filament down the 5-fold symmetry axis. ASU, asymmetric unit.

(B) Content of the asymmetric unit, colored as in (A). K33 isopeptide linkages are shown as stick representations; see Figure S5C for electron density.

(C) Close-up view of one Ub in the filament, showing interacting residues as a stick representation. Hydrogen bonds are shown as black dashed lines. The K33 and K29 side chains are also shown.

symmetry. The helix turns twice between the first and sixth molecule (Figure 5A). The asymmetric unit contains five Ub molecules and five NZF domains (Figures 5A and 5B). The electron density for K33 linkages can be discerned for one isopeptide bond (Figure S5C). The electron density for the isopeptide bonds is weak because of flexibility but also because diUb was crystallized, and each linkage in the asymmetric unit is only at half occupancy. K29 is in close proximity to the tail of the distal Ub, and it is conceivable that K29-linked polymers interact with TRABID NZF1 in a similar manner and can form similar filaments. This was supported by NMR experiments where NZF1 was added to either ^{15}N-labeled K33- or K29-linked diUb. NZF1 binding leads to chemical shift perturbations along the same face of the proximal Ub moieties, indicating that the overall orientation of the proximal Ub is similar (Figure S5D).

Interactions between Ub molecules are identical along the filament and involve exclusively polar contacts (Figure 5C). A distal Ub interacts with the Ub helix of a proximal Ub, forming hydrogen

Figure 4. Characterization of TRABID Specificity

(A) Domain structure of human TRABID. The AnkOTU fragment has been characterized in detail in Licchesi et al. (2012). Boundaries of the NZF domain fragments analyzed here are shown.

(B) Deubiquitination assay of TRABID AnkOTU against K6-, K33-, and K63-linked tetraUb. See Figure S4A for a reaction at a lower DUB concentration.

(C) Pull-down analysis of NZF fragments with a panel of diUb covering all linkage types. Left: the input chains and GST-NZF constructs used. Right: pull-down analysis shown by silver stain and anti-Ub western blot. See Figure S4B for additional controls.

(D) SPR binding experiment of NZF1 to monoUb and the eight different diUb species with error bars representing SEs. K_d values derived from two experiments are shown. See Figure S4C for best-fit parameters and values of SEs.

(E) NMR analysis of isolated NZF1 binding to ^{15}N-labeled monoUb. The chemical shift perturbation for Ub from binding to 600 μM of NZF1 is shown. Grey bars, exchange-broadened residues; asterisks, proline residues. See Figures S4E–S4G for titration data.

(F) NMR analyses as in (E) but for NZF2 and NZF3.

bonds between Gln49 (distal Ub) and Gln31 (proximal Ub) and charged interactions between Arg42 and Arg72 (distal Ub) and Asp32 (proximal Ub) (Figure 5C). This exposes the hydrophobic Ile44 and Ile36 patches of each Ub molecule and enables binding of one NZF domain to each Ile44 patch along the filament (Figure 5A).

Explaining the Specificity of TRABID NZF1 for K33 Linkages

Consistent with other linkage-specific NZF domains, NZF1 of TRABID forms a bidentate interaction across the distal and proximal Ub moieties (Figures 5B and 6A). This has been seen previously for the TAB2 and HOIL-1L NZF domains, which interact specifically with K63- and M1-linked diUb, respectively (Figure 6B; Figure S6A; Kulathu et al., 2009; Sato et al., 2009, 2011), and can be superimposed with TRABID NZF1 with low root-mean-square deviations (RMSDs) (0.5–0.6 Å). TRABID NZF1 binds the distal Ub at the Ile44 patch via the canonical NZF interaction involving Thr14, Tyr15, and Met26 (Figure 6A). This binding mode through the T-Y/F-Φ motif is conserved in all NZF interactions described to date (Figure S6B; Alam et al., 2004) and is consistent with the NMR interaction data in Figures 4E and 4F (see above). The proximal Ub is bound by TRABID NZF1 in an unusual way, at a binding site involving the start of the Ub α helix and two nearby loop regions (Figure 6A). In this interaction, Ub Glu24 makes key interactions with a complementary pocket on NZF1 formed by Tyr15, Asn17, Trp18, and Thr25. The Ub Glu24 side chain can form hydrogen bonds with side chains of these four residues (Figure 6A). In addition, the solvent-exposed TRABID NZF1 Trp18 side chain forms apolar contacts with the Asp52-Gly53 loop of the proximal Ub, and NZF1 Ser20 forms a hydrogen bond with the Gly53 carbonyl group (Figure 6A). All interacting residues in NZF1 are conserved in evolution (Figure 6C).

A comparison of the TAB2 and HOIL-1L diUb complexes reveals how NZF domains have achieved their specificity. Although the canonical interaction with a distal Ub is conserved, the proximal Ub is rotated in each complex to form distinct interactions with a second patch on the NZF domain. In the case of TAB2, the second interaction with a proximal Ub is also via the Ile44 patch (Kulathu et al., 2009; Sato et al., 2009; Figure 6B). In HOIL-1L, a short helical NZF extension contributes the secondary contacts, which are mediated by the Phe4 patch of the proximal Ub (Sato et al., 2011; Figure S6A). Superposition of structures reveals why TAB2 is unable to bind the K33 filament: Glu685 would clash directly with Glu24 of the proximal Ub (Figure S6C). Similarly, TRABID NZF1 Trp18 clashes with the proximal Ub when the TAB2-K63 diUb complex is superimposed (Figure S6D) (although mutation of this residue did not enable high-affinity K63 diUb binding; see below). Finally, the structure also reveals why TRABID NZF2 and NZF3 are unable to bind K33 polymers: Ser20 in NZF1 is replaced by Lys or Arg residues in NZF2/3 (Figure S6E), which affects binding (Figure 6E; see below). However, mutation of Lys165 in NZF3 to Ser did not enhance binding to K29/K33 chains (data not shown), suggesting that the remaining differences play a role as well. It is fascinating that, given their small size, NZF domains have evolved so many distinct binding modes to recognize different linkage types.

Validation of the TRABID-K33 Chain Interaction

To validate the interaction between TRABID NZF1 and K33-diUb biochemically, we mutated residues in the interfaces. We assembled K33-linked diUb from a Ub K11R/E24R mutant that would abrogate its interactions with the proximal interface of TRABID NZF1. Indeed, TRABID NZF1 is unable to pull down K11R/E24R diUb (Figure 6D), confirming that this Ub residue, which has not been implicated in any other Ub interaction known to us, is important for TRABID NZF1 binding.

Next, NZF1 was mutated (Figures 6E–6G; Figure S6F). NZF1 W18A and T25D were unable to interact with K33-linked diUb in pull-down experiments, and Y15F and S20R significantly weakened binding compared with wild-type NZF1 (Figure 6E). SPR measurements for these mutants interacting with K29- or K33-linked diUb revealed that, although Y15F had to be fitted to a two-site binding model with a lower K_d^{high} (11 μM), W18A and T25D fit well with a one-site binding model, indicating that they interacted only via the Ub Ile44 patch (Figures 6F and 6G; Figure S6F; Supplemental Experimental Procedures). No binding could be detected when mutating the canonical Thr14/Tyr15 (to Leu/Val, termed TY14LV), consistent with disruption of the Ile44 patch interaction.

This shows the importance of these residues for NZF1 Ub interactions, validates the observed binding mode in the structure for K33-diUb, and further confirms a similar binding mode for K29-linked chains (Figures 5 and 6). Moreover, this emphasizes that conserved residues on previously unknown proximal binding sites in NZF domains (and perhaps other small UBDs) can furnish UBDs with chain preference.

Localization of Inactive TRABID to Ub-Rich Puncta Relies on NZF1 Binding to Atypical Ub Chains

Catalytically inactive TRABID C443S (ciTRABID) localizes to Ub-rich punctate structures in cells, and this depends on its Ub-binding capability (Licchesi et al., 2012; Tran et al., 2008). TRABID contains at least six independent Ub binding interfaces: at least four in the 3xNZF module, one in the AnkUBD, and at least one in the catalytic domain. Because our biochemical analysis indicated that NZF1 provides TRABID with high-affinity binding for K29/K33 chains, we assessed how important individual NZF domains are for ciTRABID localization to puncta (Figure 7).

Mutation of the canonical Ub binding site in NZF1 (NZF1*; Figure 7A) led to a diffuse (mostly nuclear) ciTRABID localization without puncta (Figure 7B). In contrast, the same mutation in NZF2 or NZF3 (NZF2* and NZF3*, respectively) showed identical punctate pattern as ciTRABID, with a similar number of dots (Figures 7B and 7C; Figure S7A). This shows that Ub binding by NZF1 is crucial for forming punctate structures in the ciTRABID background. We also tested whether the identified mutants in the proximal Ub binding site of NZF1 are defective in punctum formation. ciTRABID W18A and ciTRABID T25D showed a reduction in the number of puncta per cell (Figures 7B and 7C; Figure S7A). This is consistent with the distal Ub binding site still being intact and maintaining residual low-affinity Ub binding capability. However, the significant reduction of dots with ciTRABID W18A suggests that the NZF1:K29/K33 interface promotes punctum formation (Figure 7D).

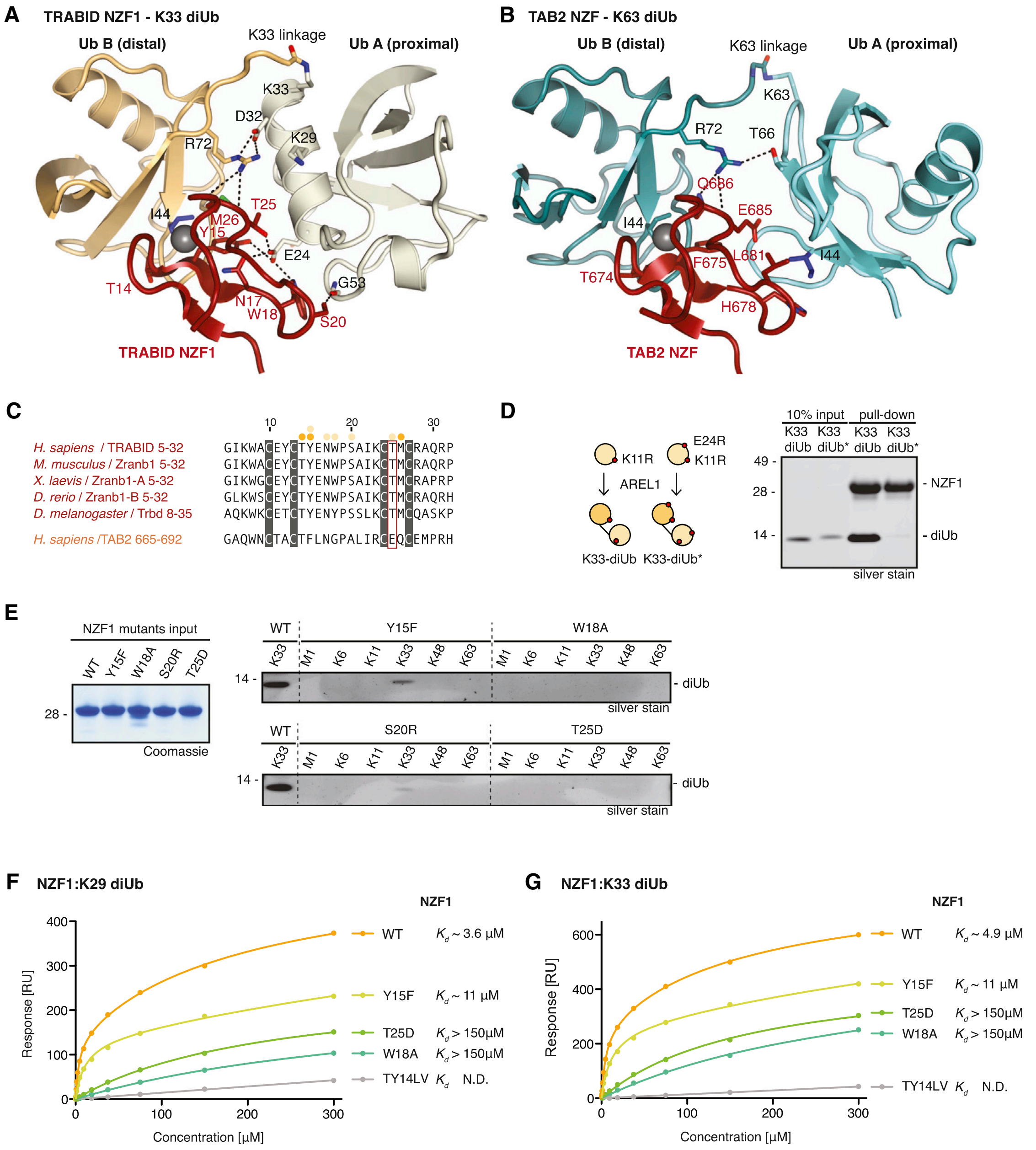

Figure 6. Explaining the K29/K33 Specificity of TRABID NZF1

(A) Detailed view of the interactions between TRABID NZF1 (red) and K33-linked diUb (orange/beige). Interacting residues are labeled, and hydrogen bonds are shown as black dashed lines.

(B) As in (A) for the TAB2 NZF interaction with K63-linked diUb (cyan).

(C) Sequence alignment of TRABID NZF1 from a diverse range of species and human TAB2 NZF domains. Interacting residues are indicated with orange (distal Ub) and beige (proximal Ub) dots. Thr25 in TRABID NZF1 is replaced with Glu685 in TAB2 NZF, which would prevent K29/K33 binding in TAB2 NZF.

(D) Left: Ub chains were assembled into K33 diUb with AREL1 using K11R or K11R/E24R Ub. Right: pull-down assays with TRABID NZF1 and diUb variants.

(E) Pull-down assays as in Figure 4C for TRABID NZF1 mutants.

(F and G) SPR binding experiment of NZF1 and its mutants against K29 diUb (F) and K33 diUb (G) with the respective K_d values indicated. SEs from two experiments are shown as error bars. See Figure S6F for values of SEs and best-fit parameters.

DISCUSSION

Chain linkage profiling by AQUA mass spectrometry is a powerful strategy to discover the missing ligases for atypical chains and to provide mechanistic insights into Ub chain assembly. Because of their mechanism of E2-independent linkage determination, HECT E3 ligases are good candidates to assemble atypical chains, and, although several have been suggested to

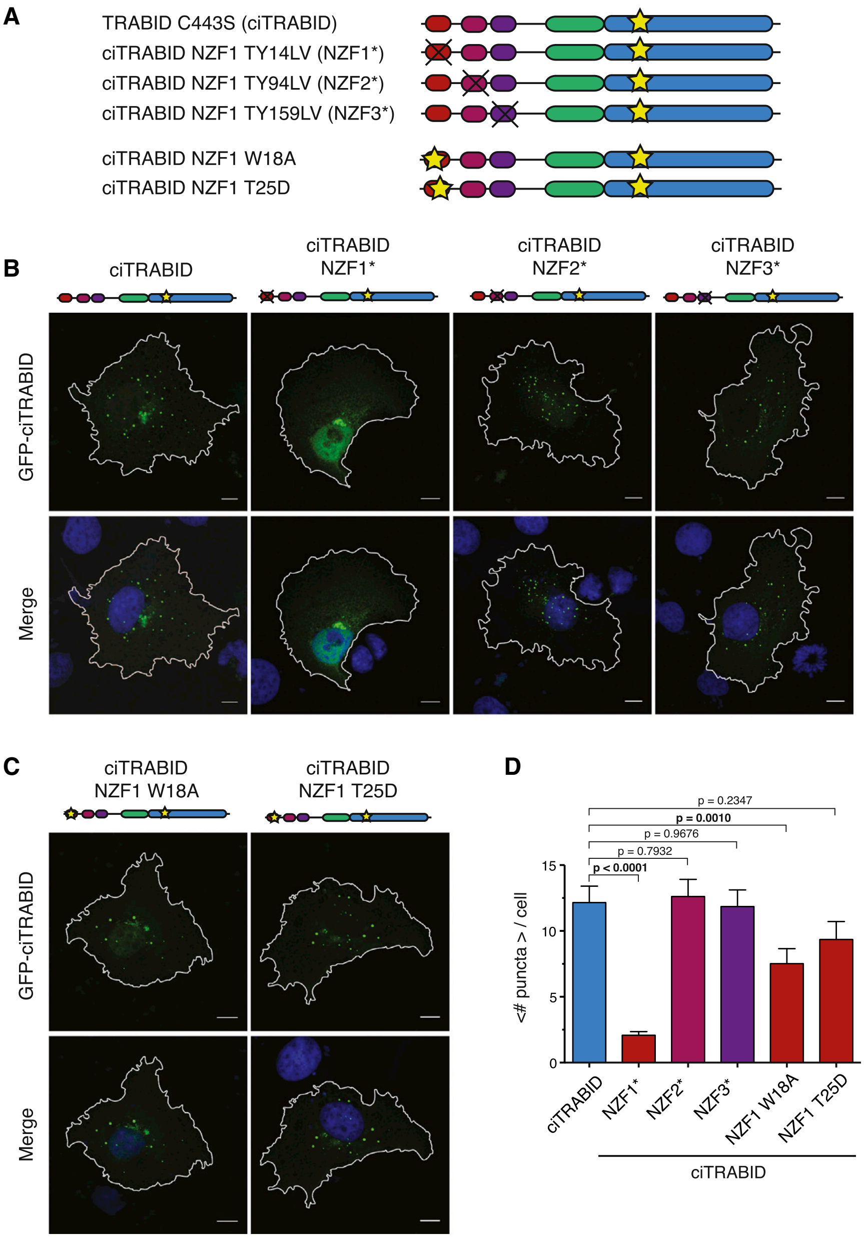

Figure 7. Localization of Catalytically Inactive TRABID Mutants in Cells

(A) Constructs used in localization experiments for GFP-TRABID fusions. Yellow stars indicate single amino acid substitutions, whereas black crosses denote two amino acid substitutions that abrogate Ub binding in the respective domain.

(B) Localization of catalytically inactive full-length GFP-TRABID (ciTRABID) constructs. GFP-ciTRABID localizes to distinct puncta in COS-7 cells. Mutations in this background that abrogate Ub binding of NZF1 (NZF1*) lead to a significant decrease in the number of dots, whereas the equivalent mutations in NZF2 (NZF2*) or NZF3 (NZF3*) do not lead to a change in the number of puncta. Cartoon representations of the constructs are shown as in (A). Scale bars, 10 μm.

(C) The same experiment with single amino acid substitutions in the proximal Ub binding site of NZF1.

(D) Statistical analysis of experiments in (B) and (C) with an average number of puncta per cell for the different mutants and corresponding SEs. p Values are given in reference to the ciTRABID mutant, and significant values ($\alpha < 0.05$) are shown in boldface. Error bars represent SEs.

assemble atypical linkages, only a subset have been characterized biochemically (Tran et al., 2013; You and Pickart, 2001). We show here that AREL1 predominantly assembles K33 linkages in free chains and on substrate proteins. Together with UBE3C, which has been reported to generate K48 and K29 linkages (You and Pickart, 2001), we provide a protocol to generate pure K29- and K33-linked polyUb enzymatically for in vitro analysis.

Our protocol to generate WT K29- or K33-linked polyUb relies on the recently discovered linkage specificity in DUBs, and we used these enzymes preparatively to remove unwanted linkage types in chains. Our redesigned forms of K48-specific OTUB1 and K63-specific AMSH are highly active and have proven to be useful for this purpose. Together with Ub chain restriction (UbiCRest) analysis (Mevissen et al., 2013), this highlights the utility of linkage-specific DUBs in studying the Ub system.

K29- and K33-linked chains are flexible and able to adopt multiple conformations, much like the remaining chain types (Ye et al., 2012). Although the diUb crystal structure has captured a compact conformation of K33-linked diUb, solution studies suggest open conformations for both chain types without formation of defined interfaces, as reported for chemically assembled K33-linked chains (Dixon et al., 2013).

Ub binding proteins can stabilize chain conformations (Ye et al., 2012), and it is therefore important to understand how polyUb is recognized by UBDs in a linkage-specific fashion. Our discovery of K29/K33 specificity in the N-terminal TRABID NZF1 domain enabled further insights into linkage-specific UBDs. NZF domains are small zinc-binding folds with remarkable linkage specificity that is achieved by bidentate interactions, whereby the ~30-aa NZF fold intercalates between and interacts with two Ub molecules. The TRABID NZF1 contacts the canonical Ile44 hydrophobic patch on the distal Ub and an unusual surface on the proximal Ub surrounding Glu24 of the Ub helix. The observed binding mode is validated by mutational analysis, which indicates that it may be shared between K29- and K33-linked chains, explaining TRABID NZF1 cross-specificity. The structure also explains why TAB2 or TRABID NZF2 and NZF3 are unable to bind K29/K33-linked chains in a similar manner.

A remarkable feature of the structure is the assembly of the K33 diUb with NZF1 into filaments that make up the entire crystal. In these filaments, each Ub-Ub contact is identical, each NZF domain binds two Ub molecules, and each Ub binds two NZF domains. The helical Ub filament bound to NZF1 domains provides an immediate model for interactions with the TRABID 3xNZF module, which, despite the lack of K29/K33 specificity in NZF2 and NZF3, could assemble on a Ub filament (Figure S7B). This is enabled by flexible linkers that vary in sequence and length but have a minimal length of 35 aa (NZF1-NZF2) and 28 aa (NZF2-NZF3) throughout evolution (Figure S7C). Such linkers would easily be able to bridge the space between adjacent NZF domains when binding a K29/K33 filament (Figure S7B).

Further studies by complementary techniques will be required to see whether K29- and K33-linked chains indeed form filaments in the presence of NZF1 in vivo. We have previously described the accumulation of catalytically inactive TRABID into characteristic Ub-containing puncta in cells, which depends on functional NZF domains (Licchesi et al., 2012). Here we extend these studies to show that, indeed, NZF1 and its K29/K33-specific binding mode are important for punctum formation. The cellular structures covered with K29/K33 chains that lead to punctum formation are intriguing and require further investigation.

Nonetheless, some new roles of K33-linked chains are emerging. AREL1 has been reported to ubiquitinate cytosolic inhibitor of apoptosis (IAP) antagonists, including SMAC, HtrA2, and ARTS, which leads to their proteasomal degradation (Kim et al., 2013). We show here that AREL1 polyubiquitinates SMAC and HtrA2 with >80% of K33 linkages in vitro. It will be interesting to see whether K33-linked chains can be linked to antiapoptotic signaling.

In cells, K33 chains have been found on AMPK kinases (together with K29-linked chains; Al-Hakim et al., 2008), on T cell receptor (TCR) ζ (Huang et al., 2010), and on Coronin-7 (Yuan et al., 2014). The latter study used an elegant Ub replacement strategy where cells express Ub K33R instead of WT Ub and revealed roles of this chain type in post-Golgi transport (Yuan et al., 2014). Interestingly, these reports and our previous work on TRABID (Licchesi et al., 2012) agreed that K33-linked chains are likely non-degradative, which is consistent with this chain type not being significantly enriched upon proteasomal inhibition (Kim et al., 2011). The ability of K33 chains to act as a proteasomal degradation signal requires further study.

Such in vivo studies of atypical chain types can now be supplemented with powerful biochemical tools reported here. The TRABID NZF1 domain could serve as an excellent tool in future studies, e.g., when used as a Ub chain sensor (Sims et al., 2012; van Wijk et al., 2012) or as a K29/K33-specific affinity reagent (Hjerpe et al., 2009). The availability of K29- and K33-linked polymers and new affinity reagents will enable a better understanding of these uncharacterized Ub signals.

EXPERIMENTAL PROCEDURES

Please see the Supplemental Experimental Procedures for more detailed information.

Protein Production

The His6-SUMO-AREL1 (436–823) and His6-SUMO-UBE3C (693–1083) constructs (both from the pOPIN-S vector) and the His6-GST-TRABID NZF construct (from the pOPIN-K vector) were expressed in Rosetta2 (DE3) pLacI cells and purified by affinity chromatography. Tags were removed by incubation with SENP1 or 3C protease. Further purification was performed by anion exchange and/or size exclusion chromatography.

Ub Chain Composition Mass Spectrometry Analysis

Ub chains were separated on a NuPAGE 4%–12% gradient gel (Invitrogen) before in-gel digestion with trypsin and the addition of Ub AQUA peptide internal standards according to Kirkpatrick et al. (2006). 10 μl of each sample was directly injected onto an EASY-Spray reverse-phase column (C18, 3 μm, 100 Å, 75 μm × 15 cm) using a Dionex UltiMate 3000 high-pressure liquid chromatography system (Thermo Fisher Scientific) and analyzed on a Q-Exactive mass spectrometer (Thermo Fisher Scientific) using parallel reaction monitoring (PRM), similar to Tsuchiya et al. (2013). Data were analyzed further according to Kirkpatrick et al. (2006).

K29 Chain Generation

K29-linked polyUb was assembled from 3 mM Ub, 1 μM E1, 10 μM UBE2L3, and 32 μM His6-SUMO UBE3C (aa 693–1083) in buffer containing 10 mM ATP, 10 mM $MgCl_2$, 40 mM Tris (pH 8.5), 100 mM NaCl, 0.6 mM DTT, and 10% (v/v) glycerol overnight at 37°C. After precipitation of enzymes by perchloric acid (0.25% [v/v]), unanchored chains were buffer-exchanged into 50 mM Tris (pH 7.4), 150 mM NaCl, and 4 mM DTT and treated with OTUB1* (1 μM), AMSH* (1 μM), and Cezanne (400 nM) for 60 min at 37°C. A second round of acid precipitation and cation exchange chromatography was used for purification.

K33 Chain Generation

K33-linked polyUb was assembled like K29-linked chains from a reaction that contained 36 μM AREL1 (aa 436–823) instead of UBE3C. The addition of 10% (v/v) glycerol in the reaction buffer prevented AREL1 precipitation during the reaction.

Pull-Down Assays

Pull-down assays were performed as described previously (Kulathu et al., 2009). Proteins were visualized by silver staining using the Silver Stain Plus kit (BioRad) according to manufacturer's protocols or by western blotting using a rabbit anti-Ub antibody (Millipore).

Nuclear Magnetic Resonance Studies

NMR experiments were performed in NMR PBS (18 mM Na_2HPO_4, 7 mM NaH_2PO_4 (pH 7.2), and 150 mM NaCl) with 5% D_2O added as a lock solvent. NMR acquisition was carried out at 298 K on a Bruker Avance III 600 MHz spectrometer equipped with a cryogenic triple resonance TCI probe. Topspin (Bruker) and Sparky (Goddard & Kneller, University of California San Francisco; http://www.cgl.ucsf.edu/home/sparky/) software packages were used for data processing and analysis, respectively. ^{1}H,^{15}N 2D BEST-TROSY experiments (Favier and Brutscher, 2011) allowed the calculation of weighted chemical shift perturbation using the equation $\sqrt{(\Delta^{1}H)^2+((\Delta^{15}N)^2/5)}$. K_d values for NZF-Ub interactions were determined according to Williamson (2013).

Crystallization, Data Collection, and Refinement

Crystals of K33-linked diUb, triUb, and of the TRABID NZF1-K33 diUb complex were grown by sitting drop vapor diffusion. Diffraction data were collected at Diamond Light Source beamlines I03 and I24, and the structures were solved by molecular replacement and refined to the final statistics in Table 1.

ACCESSION NUMBERS

Coordinates and structure factors for K33-linked di- and triUb and for TRABID NZF1-K33 diUb have been deposited with the protein data bank under accession codes 5AF4, 5AF5, and 5AF6.

SUPPLEMENTAL INFORMATION

Supplemental Information includes Supplemental Experimental Procedures and seven figures and can be found with this article online at http://dx.doi.org/10.1016/j.molcel.2015.01.042.

AUTHOR CONTRIBUTIONS

M.A.M. performed all experiments relating to K33 binding to TRABID, and P.R.E. performed experiments relating to HECT E3 ligases and structural characterization of free chains. K.N.S. performed all mass spectrometry analyses. M.S. performed localization studies, and J.N.P. contributed improved DUBs. J.L.W. and S.M.V.F. performed NMR analyses. D.K. directed the research and wrote the manuscript with input from all authors.

ACKNOWLEDGMENTS

We would like to thank Thomas Mund (LMB), Julien Licchesi (University of Bath), Sylvie Urbé (University of Liverpool), Robert Cohen (University of Colorado), Farid El Oualid and Huib Ovaa (UbiQ), and Brad Brasher (Boston Biochem) for reagents and advice and the beamline staff at Diamond Light Source beamlines I03 and I24. We also thank Stephen McLaughlin, Juliusz Mieszczanek, Deepti Gupta, Mariann Bienz, and members of the D.K. lab for reagents, discussions, and comments on the manuscript. This work was supported by the Medical Research Council (U105192732), the European Research Council (309756), the Lister Institute for Preventive Medicine, the EMBO Young Investigator Program (to D.K.), EMBO long-term fellowships (to J.N.P. and M.S.), and a Boehringer Ingelheim Fonds Ph.D. fellowship (to M.A.M.). D.K. is part of the DUB Alliance, which includes Cancer Research Technology and FORMA Therapeutics, and is a consultant for FORMA Therapeutics.

Received: August 28, 2014
Revised: December 17, 2014
Accepted: January 27, 2015
Published: March 5, 2015

REFERENCES

Al-Hakim, A.K., Zagorska, A., Chapman, L., Deak, M., Peggie, M., and Alessi, D.R. (2008). Control of AMPK-related kinases by USP9X and atypical Lys(29)/Lys(33)-linked polyubiquitin chains. Biochem. J. *411*, 249–260.

Alam, S.L., Sun, J., Payne, M., Welch, B.D., Blake, B.K., Davis, D.R., Meyer, H.H., Emr, S.D., and Sundquist, W.I. (2004). Ubiquitin interactions of NZF zinc fingers. EMBO J. *23*, 1411–1421.

Berndsen, C.E., and Wolberger, C. (2014). New insights into ubiquitin E3 ligase mechanism. Nat. Struct. Mol. Biol. *21*, 301–307.

Bremm, A., and Komander, D. (2011). Emerging roles for Lys11-linked polyubiquitin in cellular regulation. Trends Biochem. Sci. *36*, 355–363.

Bremm, A., Freund, S.M.V., and Komander, D. (2010). Lys11-linked ubiquitin chains adopt compact conformations and are preferentially hydrolyzed by the deubiquitinase Cezanne. Nat. Struct. Mol. Biol. *17*, 939–947.

Christianson, J.C., and Ye, Y. (2014). Cleaning up in the endoplasmic reticulum: ubiquitin in charge. Nat. Struct. Mol. Biol. *21*, 325–335.

Clague, M.J., Barsukov, I., Coulson, J.M., Liu, H., Rigden, D.J., and Urbé, S. (2013). Deubiquitylases from genes to organism. Physiol. Rev. *93*, 1289–1315.

Deshaies, R.J., and Joazeiro, C.A.P. (2009). RING domain E3 ubiquitin ligases. Annu. Rev. Biochem. *78*, 399–434.

Dixon, E.K., Castañeda, C.A., Kashyap, T.R., Wang, Y., and Fushman, D. (2013). Nonenzymatic assembly of branched polyubiquitin chains for structural and biochemical studies. Bioorg. Med. Chem. *21*, 3421–3429.

Favier, A., and Brutscher, B. (2011). Recovering lost magnetization: polarization enhancement in biomolecular NMR. J. Biomol. NMR *49*, 9–15.

Hershko, A., and Ciechanover, A. (1998). The ubiquitin system. Annu. Rev. Biochem. *67*, 425–479.

Hjerpe, R., Aillet, F., Lopitz-Otsoa, F., Lang, V., England, P., and Rodriguez, M.S. (2009). Efficient protection and isolation of ubiquitylated proteins using tandem ubiquitin-binding entities. EMBO Rep. *10*, 1250–1258.

Hospenthal, M.K., Freund, S.M.V., and Komander, D. (2013). Assembly, analysis and architecture of atypical ubiquitin chains. Nat. Struct. Mol. Biol. *20*, 555–565.

Huang, H., Jeon, M.-S., Liao, L., Yang, C., Elly, C., Yates, J.R., 3rd, and Liu, Y.-C. (2010). K33-linked polyubiquitination of T cell receptor-zeta regulates proteolysis-independent T cell signaling. Immunity *33*, 60–70.

Husnjak, K., and Dikic, I. (2012). Ubiquitin-binding proteins: decoders of ubiquitin-mediated cellular functions. Annu. Rev. Biochem. *81*, 291–322.

Iwai, K., Fujita, H., and Sasaki, Y. (2014). Linear ubiquitin chains: NF-κB signalling, cell death and beyond. Nat. Rev. Mol. Cell Biol. *15*, 503–508.

Kamadurai, H.B., Souphron, J., Scott, D.C., Duda, D.M., Miller, D.J., Stringer, D., Piper, R.C., and Schulman, B.A. (2009). Insights into ubiquitin transfer cascades from a structure of a UbcH5B approximately ubiquitin-HECT(NEDD4L) complex. Mol. Cell *36*, 1095–1102.

Kamadurai, H.B., Qiu, Y., Deng, A., Harrison, J.S., Macdonald, C., Actis, M., Rodrigues, P., Miller, D.J., Souphron, J., Lewis, S.M., et al. (2013). Mechanism of ubiquitin ligation and lysine prioritization by a HECT E3. eLife *2*, e00828–e00828.

Keusekotten, K., Elliott, P.R., Glockner, L., Fiil, B.K., Damgaard, R.B., Kulathu, Y., Wauer, T., Hospenthal, M.K., Gyrd-Hansen, M., Krappmann, D., et al. (2013). OTULIN antagonizes LUBAC signaling by specifically hydrolyzing Met1-linked polyubiquitin. Cell *153*, 1312–1326.

Kim, H.C., and Huibregtse, J.M. (2009). Polyubiquitination by HECT E3s and the determinants of chain type specificity. Mol. Cell. Biol. *29*, 3307–3318.

Kim, W., Bennett, E.J., Huttlin, E.L., Guo, A., Li, J., Possemato, A., Sowa, M.E., Rad, R., Rush, J., Comb, M.J., et al. (2011). Systematic and quantitative assessment of the ubiquitin-modified proteome. Mol. Cell *44*, 325–340.

Kim, J.B., Kim, S.Y., Kim, B.M., Lee, H., Kim, I., Yun, J., Jo, Y., Oh, T., Jo, Y., Chae, H.D., and Shin, D.Y. (2013). Identification of a novel anti-apoptotic E3 ubiquitin ligase that ubiquitinates antagonists of inhibitor of apoptosis proteins SMAC, HtrA2, and ARTS. J. Biol. Chem. *288*, 12014–12021.

Kirkpatrick, D.S., Hathaway, N.A., Hanna, J., Elsasser, S., Rush, J., Finley, D., King, R.W., and Gygi, S.P. (2006). Quantitative analysis of in vitro ubiquitinated cyclin B1 reveals complex chain topology. Nat. Cell Biol. *8*, 700–710.

Komander, D., and Rape, M. (2012). The ubiquitin code. Annu. Rev. Biochem. *81*, 203–229.

Komander, D., Clague, M.J., and Urbé, S. (2009). Breaking the chains: structure and function of the deubiquitinases. Nat. Rev. Mol. Cell Biol. *10*, 550–563.

Kulathu, Y., and Komander, D. (2012). Atypical ubiquitylation - the unexplored world of polyubiquitin beyond Lys48 and Lys63 linkages. Nat. Rev. Mol. Cell Biol. *13*, 508–523.

Kulathu, Y., Akutsu, M., Bremm, A., Hofmann, K., and Komander, D. (2009). Two-sided ubiquitin binding explains specificity of the TAB2 NZF domain. Nat. Struct. Mol. Biol. *16*, 1328–1330.

Licchesi, J.D.F., Mieszczanek, J., Mevissen, T.E.T., Rutherford, T.J., Akutsu, M., Virdee, S., El Oualid, F., Chin, J.W., Ovaa, H., Bienz, M., and Komander, D. (2012). An ankyrin-repeat ubiquitin-binding domain determines TRABID's specificity for atypical ubiquitin chains. Nat. Struct. Mol. Biol. *19*, 62–71.

Maspero, E., Valentini, E., Mari, S., Cecatiello, V., Soffientini, P., Pasqualato, S., and Polo, S. (2013). Structure of a ubiquitin-loaded HECT ligase reveals the molecular basis for catalytic priming. Nat. Struct. Mol. Biol. *20*, 696–701.

Mattiroli, F., and Sixma, T.K. (2014). Lysine-targeting specificity in ubiquitin and ubiquitin-like modification pathways. Nat. Struct. Mol. Biol. *21*, 308–316.

Mevissen, T.E.T., Hospenthal, M.K., Geurink, P.P., Elliott, P.R., Akutsu, M., Arnaudo, N., Ekkebus, R., Kulathu, Y., Wauer, T., El Oualid, F., et al. (2013). OTU deubiquitinases reveal mechanisms of linkage specificity and enable ubiquitin chain restriction analysis. Cell *154*, 169–184.

Ritorto, M.S., Ewan, R., Perez-Oliva, A.B., Knebel, A., Buhrlage, S.J., Wightman, M., Kelly, S.M., Wood, N.T., Virdee, S., Gray, N.S., et al. (2014).

Screening of DUB activity and specificity by MALDI-TOF mass spectrometry. Nat. Commun. *5*, 4763.

Rivkin, E., Almeida, S.M., Ceccarelli, D.F., Juang, Y.-C., MacLean, T.A., Srikumar, T., Huang, H., Dunham, W.H., Fukumura, R., Xie, G., et al. (2013). The linear ubiquitin-specific deubiquitinase gumby regulates angiogenesis. Nature *498*, 318–324.

Rotin, D., and Kumar, S. (2009). Physiological functions of the HECT family of ubiquitin ligases. Nat. Rev. Mol. Cell Biol. *10*, 398–409.

Sato, Y., Yoshikawa, A., Yamashita, M., Yamagata, A., and Fukai, S. (2009). Structural basis for specific recognition of Lys 63-linked polyubiquitin chains by NZF domains of TAB2 and TAB3. EMBO J. *28*, 3903–3909.

Sato, Y., Fujita, H., Yoshikawa, A., Yamashita, M., Yamagata, A., Kaiser, S.E., Iwai, K., and Fukai, S. (2011). Specific recognition of linear ubiquitin chains by the Npl4 zinc finger (NZF) domain of the HOIL-1L subunit of the linear ubiquitin chain assembly complex. Proc. Natl. Acad. Sci. USA *108*, 20520–20525.

Scheffner, M., Huibregtse, J.M., Vierstra, R.D., and Howley, P.M. (1993). The HPV-16 E6 and E6-AP complex functions as a ubiquitin-protein ligase in the ubiquitination of p53. Cell *75*, 495–505.

Schulman, B.A., and Harper, J.W. (2009). Ubiquitin-like protein activation by E1 enzymes: the apex for downstream signalling pathways. Nat. Rev. Mol. Cell Biol. *10*, 319–331.

Shaid, S., Brandts, C.H., Serve, H., and Dikic, I. (2013). Ubiquitination and selective autophagy. Cell Death Differ. *20*, 21–30.

Sims, J.J., Scavone, F., Cooper, E.M., Kane, L.A., Youle, R.J., Boeke, J.D., and Cohen, R.E. (2012). Polyubiquitin-sensor proteins reveal localization and linkage-type dependence of cellular ubiquitin signaling. Nat. Methods *9*, 303–309.

Tran, H., Hamada, F., Schwarz-Romond, T., and Bienz, M. (2008). Trabid, a new positive regulator of Wnt-induced transcription with preference for binding and cleaving K63-linked ubiquitin chains. Genes Dev. *22*, 528–542.

Tran, H., Bustos, D., Yeh, R., Rubinfeld, B., Lam, C., Shriver, S., Zilberleyb, I., Lee, M.W., Phu, L., Sarkar, A.A., et al. (2013). HectD1 E3 ligase modifies adenomatous polyposis coli (APC) with polyubiquitin to promote the APC-axin interaction. J. Biol. Chem. *288*, 3753–3767.

Tsuchiya, H., Tanaka, K., and Saeki, Y. (2013). The parallel reaction monitoring method contributes to a highly sensitive polyubiquitin chain quantification. Biochem. Biophys. Res. Commun. *436*, 223–229.

van Wijk, S.J.L., Fiskin, E., Putyrski, M., Pampaloni, F., Hou, J., Wild, P., Kensche, T., Grecco, H.E., Bastiaens, P., and Dikic, I. (2012). Fluorescence-based sensors to monitor localization and functions of linear and K63-linked ubiquitin chains in cells. Mol. Cell *47*, 797–809.

Varadan, R., Walker, O., Pickart, C., and Fushman, D. (2002). Structural properties of polyubiquitin chains in solution. J. Mol. Biol. *324*, 637–647.

Varadan, R., Assfalg, M., Haririnia, A., Raasi, S., Pickart, C., and Fushman, D. (2004). Solution conformation of Lys63-linked di-ubiquitin chain provides clues to functional diversity of polyubiquitin signaling. J. Biol. Chem. *279*, 7055–7063.

Vijay-Kumar, S., Bugg, C.E., and Cook, W.J. (1987). Structure of ubiquitin refined at 1.8 A resolution. J. Mol. Biol. *194*, 531–544.

Virdee, S., Ye, Y., Nguyen, D.P., Komander, D., and Chin, J.W. (2010). Engineered diubiquitin synthesis reveals Lys29-isopeptide specificity of an OTU deubiquitinase. Nat. Chem. Biol. *6*, 750–757.

Wagner, S.A., Beli, P., Weinert, B.T., Nielsen, M.L., Cox, J., Mann, M., and Choudhary, C. (2011). A proteome-wide, quantitative survey of in vivo ubiquitylation sites reveals widespread regulatory roles. Mol Cell Proteomics, M111.013284.

Wickliffe, K.E., Williamson, A., Meyer, H.-J., Kelly, A., and Rape, M. (2011). K11-linked ubiquitin chains as novel regulators of cell division. Trends Cell Biol. *21*, 656–663.

Williamson, M.P. (2013). Using chemical shift perturbation to characterise ligand binding. Prog. Nucl. Magn. Reson. Spectrosc. *73*, 1–16.

Xu, P., Duong, D.M., Seyfried, N.T., Cheng, D., Xie, Y., Robert, J., Rush, J., Hochstrasser, M., Finley, D., and Peng, J. (2009). Quantitative proteomics reveals the function of unconventional ubiquitin chains in proteasomal degradation. Cell *137*, 133–145.

Ye, Y., and Rape, M. (2009). Building ubiquitin chains: E2 enzymes at work. Nat. Rev. Mol. Cell Biol. *10*, 755–764.

Ye, Y., Blaser, G., Horrocks, M.H., Ruedas-Rama, M.J., Ibrahim, S., Zhukov, A.A., Orte, A., Klenerman, D., Jackson, S.E., and Komander, D. (2012). Ubiquitin chain conformation regulates recognition and activity of interacting proteins. Nature *492*, 266–270.

You, J., and Pickart, C.M. (2001). A HECT domain E3 enzyme assembles novel polyubiquitin chains. J. Biol. Chem. *276*, 19871–19878.

Yuan, W.-C., Lee, Y.-R., Lin, S.-Y., Chang, L.-Y., Tan, Y.P., Hung, C.-C., Kuo, J.-C., Liu, C.-H., Lin, M.-Y., Xu, M., et al. (2014). K33-Linked Polyubiquitination of Coronin 7 by Cul3-KLHL20 Ubiquitin E3 Ligase Regulates Protein Trafficking. Mol. Cell *54*, 586–600.

Molecular Cell, Volume *58*

Supplemental Information

Assembly and Specific Recognition of K29- and K33-Linked Polyubiquitin

Martin A. Michel, Paul R. Elliott, Kirby N. Swatek, Michal Simicek, Jonathan N. Pruneda, Jane L. Wagstaff, Stefan M.V. Freund, and David Komander

Figure S1

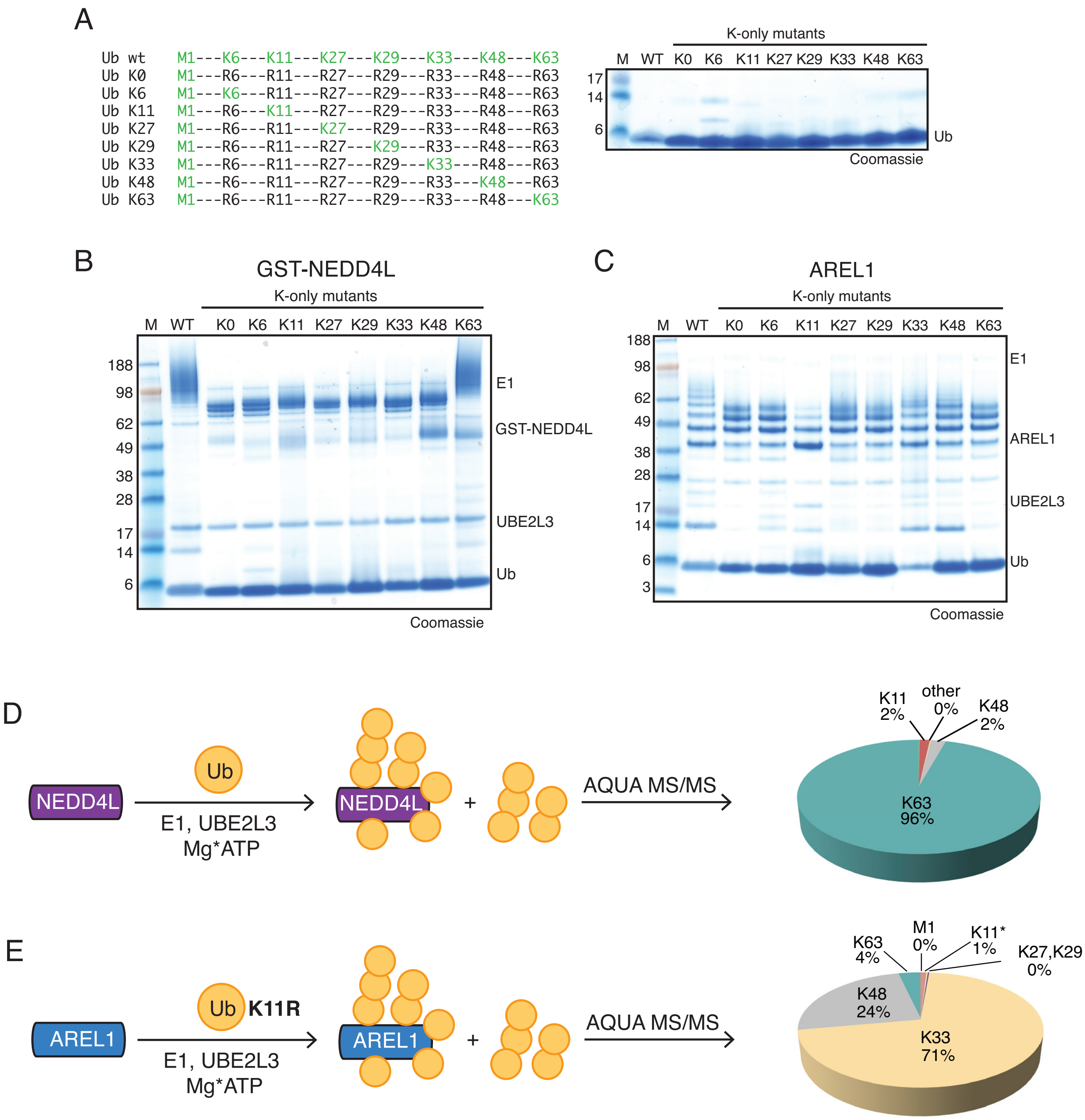

A
Ub wt M1---K6---K11---K27---K29---K33---K48---K63
Ub K0 M1---R6---R11---R27---R29---R33---R48---R63
Ub K6 M1---K6---R11---R27---R29---R33---R48---R63
Ub K11 M1---R6---K11---R27---R29---R33---R48---R63
Ub K27 M1---R6---R11---K27---R29---R33---R48---R63
Ub K29 M1---R6---R11---R27---K29---R33---R48---R63
Ub K33 M1---R6---R11---R27---R29---K33---R48---R63
Ub K48 M1---R6---R11---R27---R29---R33---K48---R63
Ub K63 M1---R6---R11---R27---R29---R33---R48---K63
K-only mutants
M WT K0 K6 K11 K27 K29 K33 K48 K63
17
14
6
Ub
Coomassie
B
GST-NEDD4L
K-only mutants
M WT K0 K6 K11 K27 K29 K33 K48 K63
188
98
62
49
38
28
17
14
6
E1
GST-NEDD4L
UBE2L3
Ub
Coomassie
C
AREL1
K-only mutants
M WT K0 K6 K11 K27 K29 K33 K48 K63
188
98
62
49
38
28
17
14
6
3
E1
AREL1
UBE2L3
Ub
Coomassie
D
NEDD4L
Ub
E1, UBE2L3
Mg*ATP
NEDD4L
+
AQUA MS/MS
K11 2%
other 0%
K48 2%
K63 96%
E
AREL1
Ub K11R
E1, UBE2L3
Mg*ATP
AREL1
+
AQUA MS/MS
K63 4%
M1 0%
K11* 1%
K27,K29 0%
K48 24%
K33 71%

Figure S1 (related to Figure 1). HECT specificity analysis with K-only Ub mutants.

A) Schematic of K-only Ub mutants, and Coomassie-stained SDS-PAGE gel showing input monoUb. **B)** Specificity of GST-tagged NEDD4L (aa 576-955) which is K63 specific (Kamadurai et al., 2009) using a panel of K-only Ub mutants as indicated. Samples were resolved by SDS-PAGE and proteins visualized by Coomassie staining. **C)** Analysis as in **B** for AREL1. **D)** Linkage composition analyzed by AQUA mass-spectrometry for NEDD4L, demonstrating K63-specificity with wt Ub. **E)** Linkage composition analyzed with AQUA mass-spectrometry for AREL1, as in **Figure 1C**, but using Ub K11R (K6-linkage was excluded from the quantitative analysis due to the K11R substitution).

Figure S2

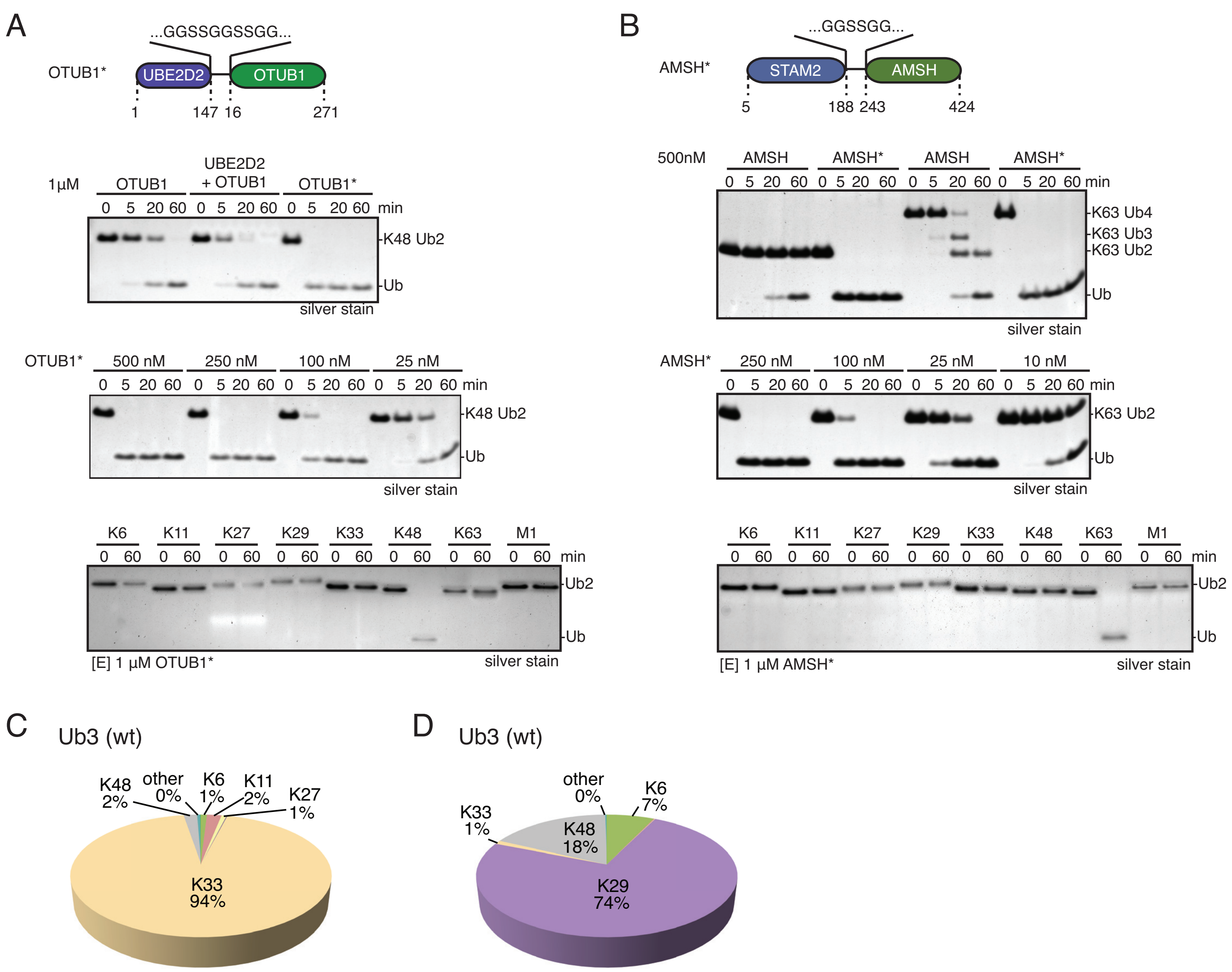

A
...GGSSGGSSGG...
OTUB1*
UBE2D2
OTUB1
1
147 16
271
1µM
OTUB1
UBE2D2 + OTUB1
OTUB1*
0 5 20 60 0 5 20 60 0 5 20 60 min
K48 Ub2
Ub
silver stain
OTUB1*
500 nM
250 nM
100 nM
25 nM
0 5 20 60 0 5 20 60 0 5 20 60 0 5 20 60 min
K48 Ub2
Ub
silver stain
K6 K11 K27 K29 K33 K48 K63 M1
0 60 0 60 0 60 0 60 0 60 0 60 0 60 0 60 min
Ub2
Ub
[E] 1 µM OTUB1*
silver stain
B
...GGSSGG...
AMSH*
STAM2
AMSH
5
188 243
424
500nM
AMSH
AMSH*
AMSH
AMSH*
0 5 20 60 0 5 20 60 0 5 20 60 0 5 20 60 min
K63 Ub4
K63 Ub3
K63 Ub2
Ub
silver stain
AMSH*
250 nM
100 nM
25 nM
10 nM
0 5 20 60 0 5 20 60 0 5 20 60 0 5 20 60 min
K63 Ub2
Ub
silver stain
K6 K11 K27 K29 K33 K48 K63 M1
0 60 0 60 0 60 0 60 0 60 0 60 0 60 0 60 min
Ub2
Ub
[E] 1 µM AMSH*
silver stain
C
Ub3 (wt)
K48 2%
other 0%
K6 1%
K11 2%
K27 1%
K33 94%
D
Ub3 (wt)
other 0%
K6 7%
K33 1%
K48 18%
K29 74%

Figure S2 (related to Figure 2). Purification of unanchored K29/K33 polyUb chains.

A) The UBE2D2-OTUB1 fusion (OTUB1*; see cartoon) is K48-specific and displays significantly higher activity against K48-linked diUb. A deubiquitinase assay is shown of wt OTUB1, OTUB1 mixed with UBE2D2 and the UBE2D2-OTUB1 fusion (OTUB1*) (*top*). (*Middle*) OTUB1* is able to hydrolyze 3 μM diUb at 25 nM enzyme concentration within 60 min. (*Bottom*) OTUB1* is still specific against K48-linked diUb at 1 μM DUB concentration when compared to all other linkage types. **B)** Comparison of AMSH activity versus STAM2-AMSH fusion (AMSH*; see cartoon) against diUb and tetraUb (*top*). AMSH* is able to hydrolyze 3 μM diUb at 25 nM DUB concentration within 60 min (*middle*). (*Bottom*) AMSH* retains its specificity against K63 diUb at 1 μM DUB concentration. **C)** AQUA mass-spectrometry profile for purified wt K33 triUb. **D)** AQUA mass-spectrometry profile for purified wt K29 triUb.

Figure S3

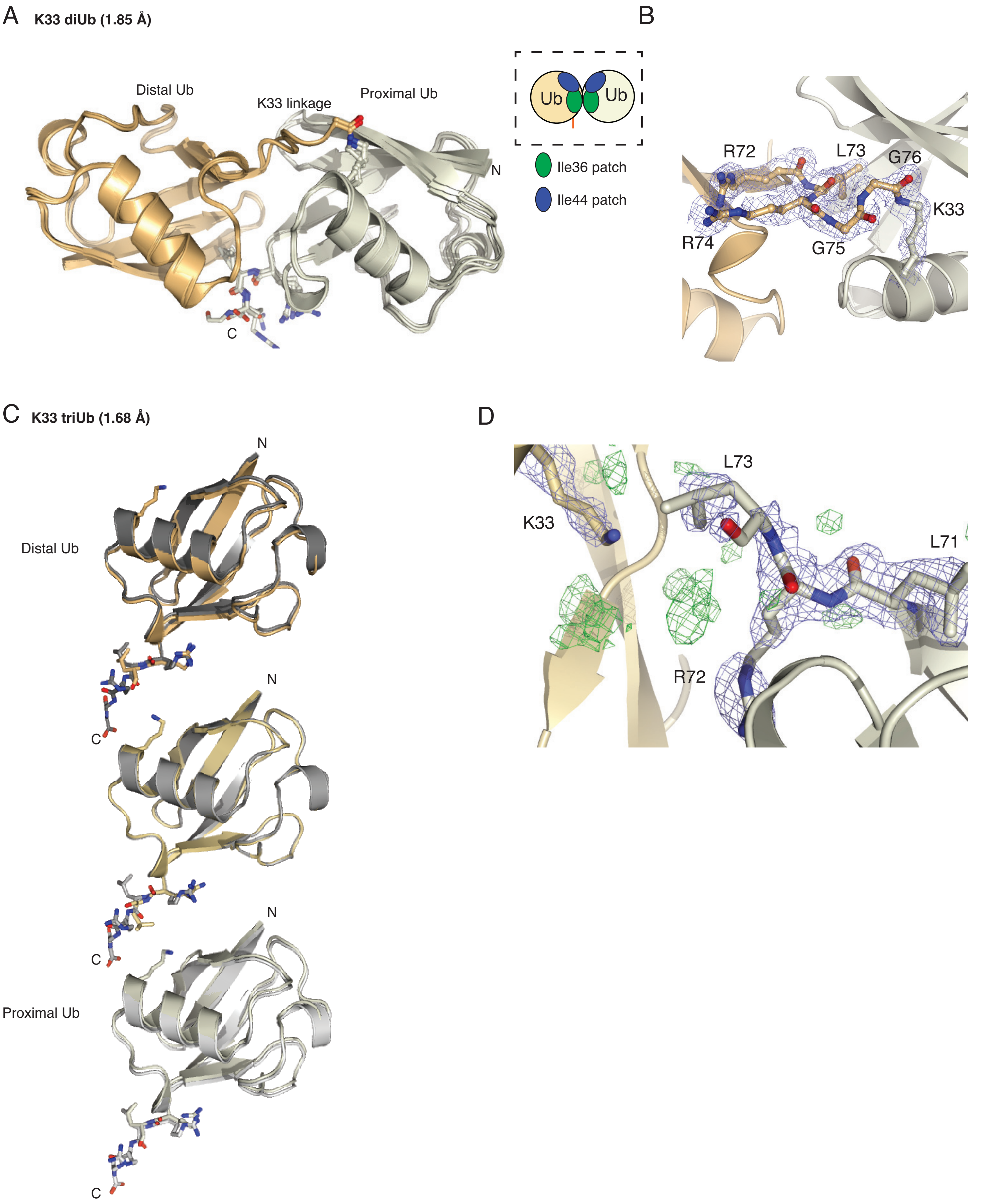
A
K33 diUb (1.85 Å)
Distal Ub
K33 linkage
Proximal Ub
N
C
Ub
Ub
Ile36 patch
Ile44 patch
B
R72
L73
G76
K33
R74
G75
C
K33 triUb (1.68 Å)
N
Distal Ub
C
N
C
N
Proximal Ub
C
D
L73
K33
L71
R72

Figure S3 (related to Figure 3). Structures of K33-linked diUb and triUb. **A)** Crystal structure of K33-linked diUb. The four individual diUb molecules from the asymmetric unit (ASU) are shown superimposed. The isopeptide bond is shown in ball and stick representation, as is the C-terminus of the proximal moiety. (*Insert cartoon*) the Ile36 patch (green) is at the center of the two-fold pseudo-symmetry of the K33 diUb structure and makes the Ile44 patch (blue) appear as a continuous surface across the two Ub moieties. **B)** 2|Fo|-|Fc| electron density, contoured at 1 σ, for the isopeptide linkage in K33-linked diUb. Linkage residues are shown in ball-and-stick representation with red oxygen and blue nitrogen atoms. **C)** Structure of K33-linked triUb. The asymmetric unit contains one Ub and two neighboring ASUs are shown. A K33-linked isopeptide bond can only extend between these symmetry-related molecules. As a comparison, equivalent Ub moieties are shown for monoUb, crystallized in the same space group (1ubq {VijayKumar:1987wy}) and are shown superimposed in shades of grey. Residues corresponding to the Ub C-terminus are shown as sticks for both monoUb and K33 triUb. **D)** Close-up view of the proximal K33 and the C-terminus of the distal Ub moiety. A weighted 2|Fo|-|Fc| electron density map is shown, contoured at 0.8 σ (blue) and a weighted |Fo|-|Fc| difference map showing positive density (green) is shown, contoured at 2.9 σ. Owing to the flexibility of the C-terminus lack of contiguous electron density in this region, no residues were modeled into the density.

Figure S4

A

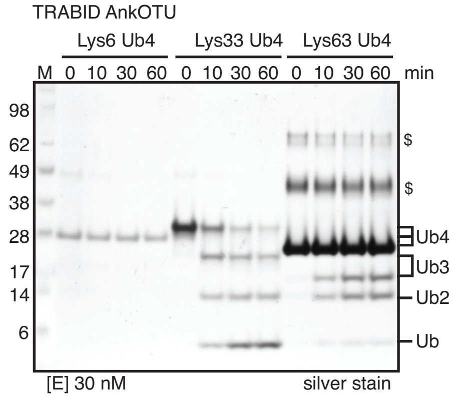

B

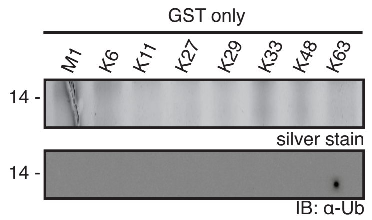

C

	NZF1			
	K_d^{low} (µM)	SEM^{low}	K_d^{high} (µM)	SEM^{high}
monoUb	370	22.9	N.D.	N.D.
M1 diUb	186	5.3	N.D.	N.D.
K6 diUb	295	10.2	N.D.	N.D.
K11 diUb	224	5.6	N.D.	N.D.
K27 diUb	222	7.4	N.D.	N.D.
K29 diUb	201	19.5	3.6	0.3
K33 diUb	179	12.3	4.9	0.2
K48 diUb	339	11.9	N.D.	N.D.
K63 diUb	272	15.9	N.D.	N.D.

D

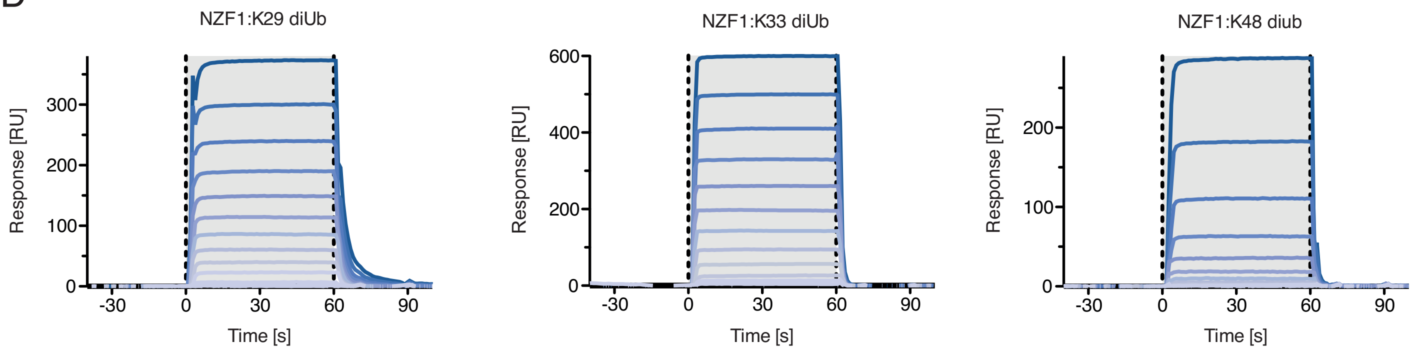

E

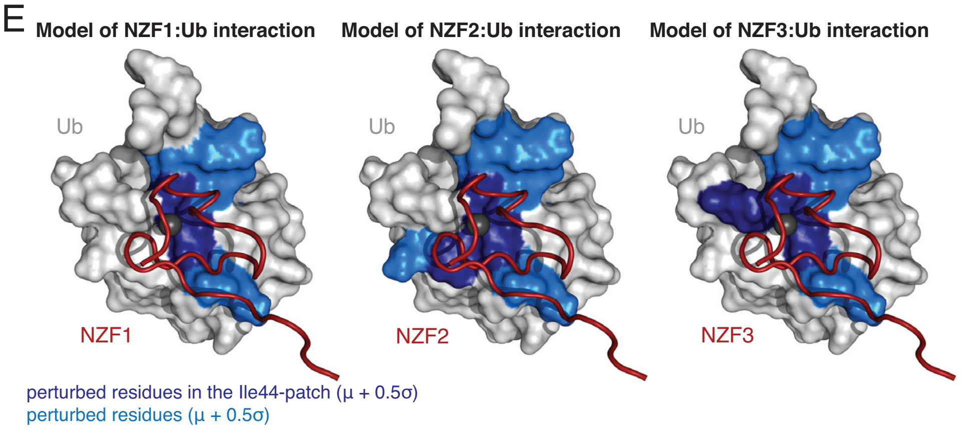

F

	K_d (µM)		
Residue	NZF1	NZF2	NZF3
V5	480	800	440
K6	370	780	370
L8	600	800	270
Q41	420	1000	510
L50	400	1480	1020
V70	430	1410	630
L73	370	830	560
$\langle K_d \rangle$	440	1010	540
STD	30	110	90

G

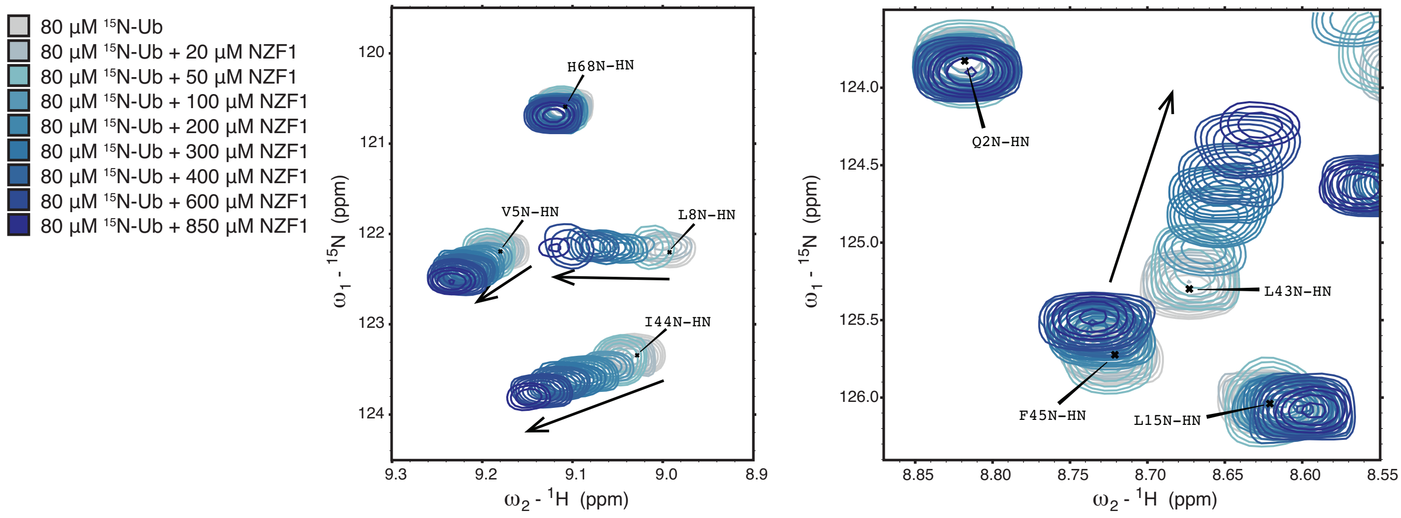

Figure S4 (related to Figure 4). Biochemistry of TRABID Ub interactions. **A)** DUB assay of TRABID AnkOTU against K6-, K33- and K63-linked tetraUb at 30 nM concentration. **B)** Pull-down of a panel of diUb molecules as in **Figure 4C** with GST as a control. **C)** Standard errors of the mean and best-fit parameters for K_ds of the SPR binding experiments for NZF1 to monoUb and the eight differently linked diUbs are shown. The values are derived from two independent experiments. N.D. indicates that binding was not detectable. K29 and K33 diUb were fitted to a two-site binding model with two K_d values to account for the low-affinity binding to the Ile44 patch of the proximal Ub as well as the higher affinity binding to both the Ile44 patch of the distal Ub and the Glu24 site of the proximal Ub at the same time. **D)** Representative SPR traces for experiments in **Figure 4D** for K29 diUb, K33 diUb and K48 diUb. A gradient from dark to light indicates traces for decreasing concentrations of NZF1. Dotted lines show the start and end of injection. **E)** Structure of Npl4 NZF domain bound to monoUb (pdb-id 1q5w, (Alam et al., 2004)), showing Ub under a surface and the model NZF domain as ribbon to indicate the binding site. Residues with significant chemical shift perturbations (μ+0.5σ) on monoUb upon binding to NZF1, NZF2 or NZF3 of TRABID (*left to right*) were mapped on the surface of Ub, in blue (Ile44, Val70, Leu8) and light blue (others). **F)** Binding constants of individual TRABID NZF domains for monoUb as derived by NMR analysis for individual perturbed resonances as indicated. The average K_d is quoted in the main text. **G)** Titration analysis of NZF domain binding to ^{15}N-labeled Ub by NMR. Data for selected resonances is shown for NZF1.

Figure S5

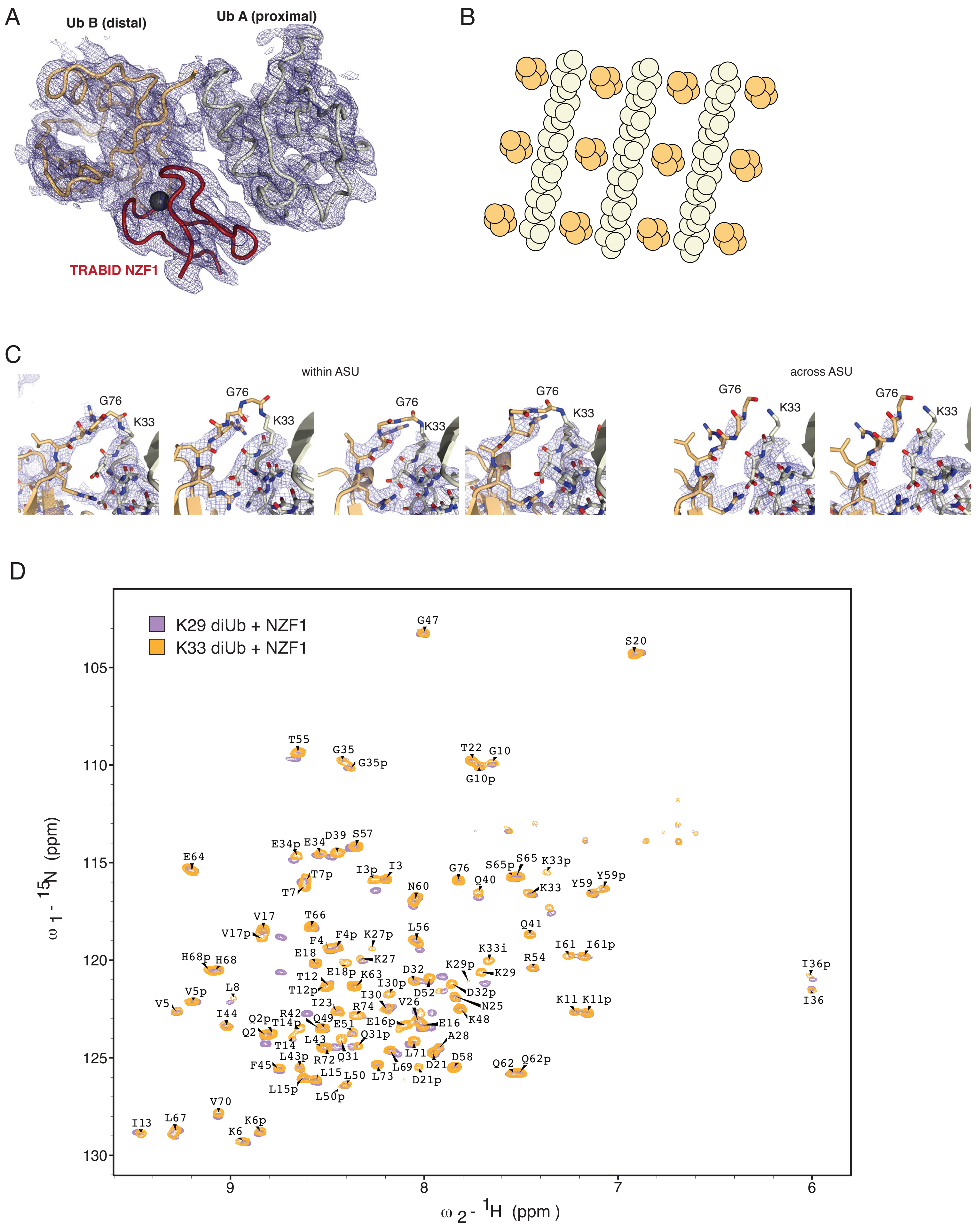

A
Ub B (distal)
Ub A (proximal)
TRABID NZF1
B
C
within ASU
across ASU
G76
K33
D
K29 diUb + NZF1
K33 diUb + NZF1
ω 1 - 15N (ppm)
ω 2 - 1H (ppm)

Figure S5 (related to Figure 5). Structure of TRABID NZF1:K33-linked diUb.

A) Overall view of electron density maps for a K33 diUb:NZF1 complex in the ASU. A 2|Fo|-|Fc| map is shown contoured at 1σ. **B)** Schematic depiction of K33 filaments in the crystal. **C)** 2|Fo|-|Fc| electron density, contoured at 1σ, for residues of the isopeptide bonds between Ub molecules in the ASU (left four panels) and across ASU (right panels). Electron density at the linkage points suffers from low occupancy (0.5) since diUb was crystallized. **D)** BEST-TROSY spectra of ^{15}N-labeled K33 diUb and ^{15}N-labeled K29 diUb each bound to NZF1 at equimolar concentration. Assigned peaks for K33 diUb are labeled. Many peaks overlap suggesting a similar binding mode of NZF1 to K29 and K33 chains.

Figure S6

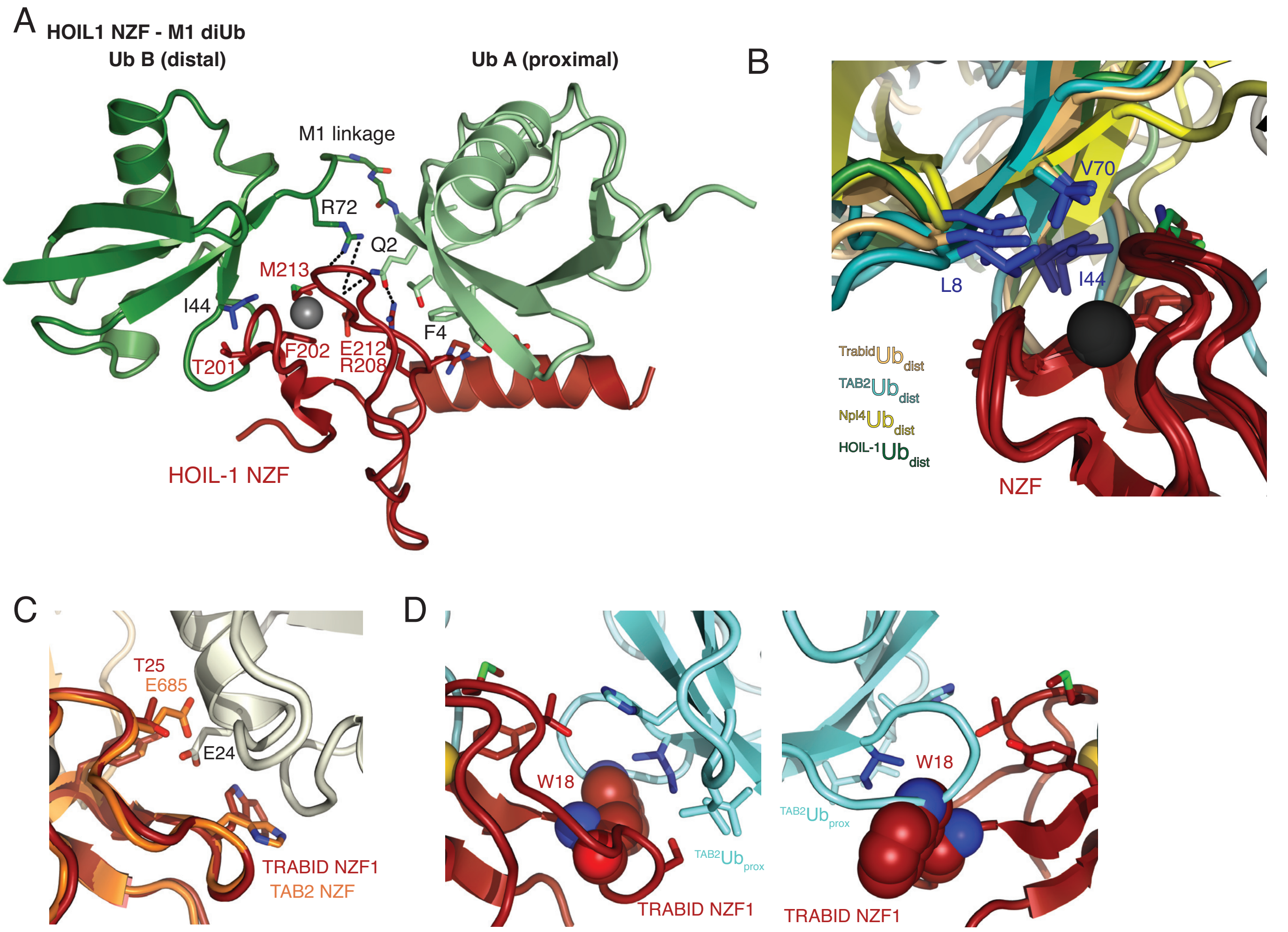

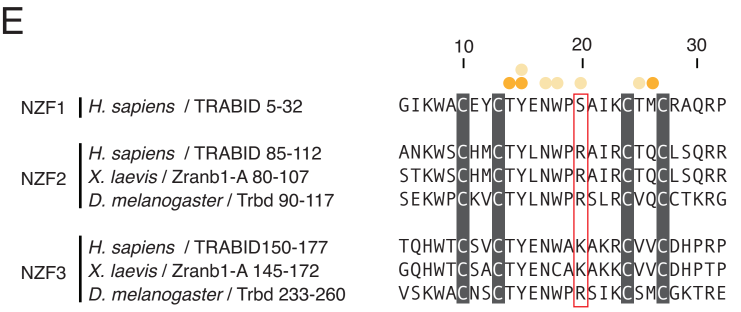

F

		NZF1				
		wt	TY14LV	Y15F	W18A	T25D
K29 diUb	K_d^{low} (μM)	201	N.D.	241	419	229
	SEM^{low}	9.4	N.D.	36.2	20.3	6.1
	K_d^{high} (μM)	3.6	N.D.	10.7	N.D.	N.D.
	SEM^{high}	0.1	N.D.	0.5	N.D.	N.D.
K33 diUb	K_d^{low} (μM)	179	N.D.	251	543	476
	SEM^{low}	5.3	N.D.	28.3	16.2	5.4
	K_d^{high} (μM)	4.9	N.D.	11.4	N.D.	N.D.
	SEM^{high}	0.1	N.D.	0.4	N.D.	N.D.

Figure S6 (related to Figure 6). Specificity of NZF1 for K33-linked chains

A) Structure of HOIL1 NZF domain (red) bound to M1-linked diUb (green). Key residues are depicted in stick representation and hydrogen bonds are indicated as black dashed lines. The proximal Ub interacts *via* its Phe4 patch with a helical extension of the NZF fold. **B)** Structures of NZF domain-Ub complexes (colored as indicated) superimposed on the NZF, which interacts with the Ile44 patch on the distal Ub. **C)** Superposition of TAB2 onto the TRABID NZF1:K33 diUb complex focusing on the proximal Ub binding site. TAB2 Glu685 clashes with Ub Glu24 in the K33 filament. **D)** Superposition of TRABID NZF1 onto the TAB2:K63 diUb complex focusing on the proximal Ub binding site. TRABID Trp18 clashes with the β3-β4 loop of Ub, preventing this Ub orientation. **E)** Alignment of human TRABID NZF1, with TRABID NZF2 and NZF3 from different species. Ser20 in NZF1 is replaced with Arg or Lys NZF2/3, explaining why these NZF domains cannot bind the proximal Ub in an analogous fashion to NZF1. See **Figure 6E** where the NZF1 S20R mutant is tested in pull-down analysis. **F)** A table with best-fit binding parameters from the SPR data for the different NZF1 constructs to K29- and K33-linked diUb are shown. N.D. denotes that no binding could be detected. All data was fitted to a one-site binding model, except for the wt and Y15F constructs that were better described by a two-site binding model (see **Supplementary Experimental Procedures**) and have thus two indicated K_ds.

Figure S7

A

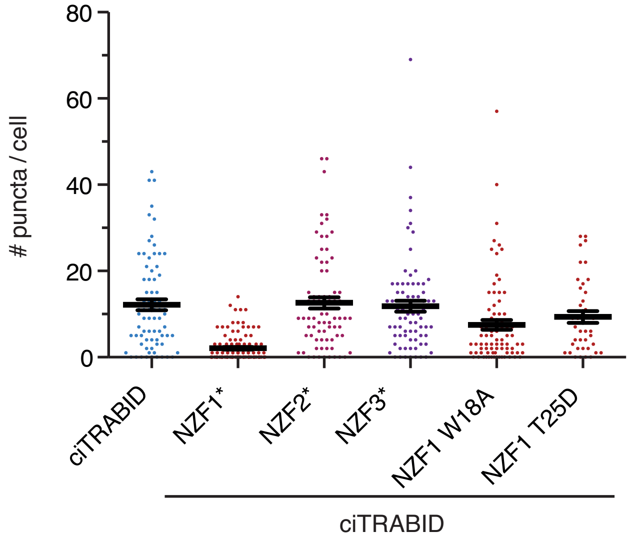

	n	<puncta>	SEM
ciTRABID	76	12.2	1.3
NZF1*	118	2.1	0.3
NZF2*	76	12.6	1.3
NZF3*	78	11.9	1.3
NZF1 W18A	79	7.5	1.1
NZF1 T25D	41	9.8	1.4

B

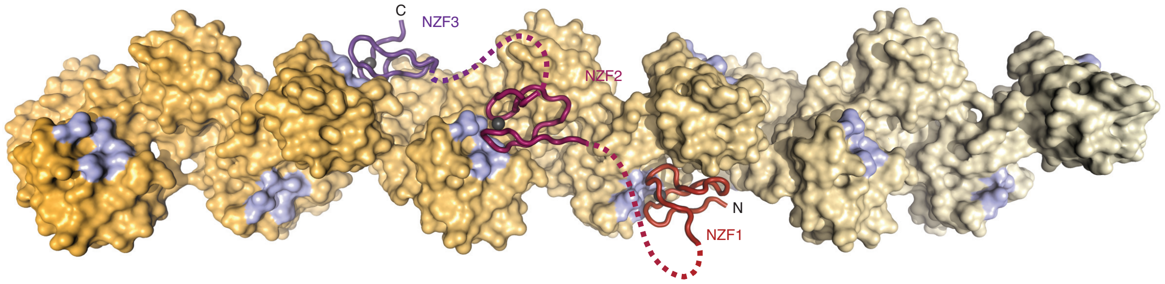

C

H. sapiens / TRABID 33-84 SGTIITEDPFKSGSSDVGRDWDPSSTEGGSSPLICPDSSARPRVKSSYSM-EN
X. laevis / Zranb1-A 33-79 SGAIITEEPFKNSTPDVGSM-----ERDIGSPLICPDSSARPRVKSSYSM-EP
D. rerio / Zranb1-B 33-82 NAPIITEEPFKSSSSLDP---SLCTTQGGSTLLICPDSSARPRVRIADELPET
H. vulgaris / Zranb1 36-70 QN---------------NRWNLVLDETNNDEIINEEIILLS--EKNSANNDK

NZF1 NZF2

H. sapiens / TRABID 113-147 TRSPTESPQS-----SGSGSRPVAFSVDPCEEYNDRNKLN
X. laevis / Zranb1-A 108-142 IRSPTESPQS-----SGSGLRSIPSPIDPCEEYNDRNKLN
D. rerio / Zranb1-B 111-150 QGSQQHSPLSPSETPQTSGSRPSPVTSDPCEEYNDRNRLN
H. vulgaris / Zranb1 109-136 DEFKLVESLKN------------IENNAECRSDLKERKTI

NZF2 NZF3

shortest linkers
NZF1-NZF2: 35 aa
NZF2-NZF3: 28 aa

Figure S7 (related to Figure 7 and Discussion). Analysis of TRABID localization in cells and potential TRABID 3xNZF:K33 filament interaction.

A) *(Left)* Graph summarizing localization data for the different mutants. Each point represents the number of puncta of a single cell. Averages and standard errors are indicated in black. *(Right)* A table summarizing the number of analyzed cells as well as the averages and standard deviations for the different mutants. **B)** Model for TRABID 3xNZF interactions with K33 filaments. Three NZF1 domains are depicted, and their respective N- and C-termini are connected by linkers indicated as dashed lines. A direct connection between NZF domains spans 31 Å. **C)** TRABID NZF linkers are not conserved in sequence or length, but have a minimum length of 35 aa (NZF1-NZF2) or 28 aa (NZF2-NZF3) in TRABID from distinct species. Such linkers would span the required distance easily.

Supplementary Experimental Procedures

Molecular Biology

The coding sequences of AREL1 (aa 436-823), UBE3C (693-1083) and TRABID NZF constructs (NZF1: 1-33; NZF2: 84-114; NZF3: 148-178; NZF3+: 147-263; NZF1-3: 1-178; NZF1-3+: 1-263) were amplified using KOD HotStart DNA polymerase (Novagen). AREL1 and UBE3C PCR products were cloned into pOPIN-S, which encodes an N-terminal His6-SUMO tag (Berrow et al., 2007) whilst TRABID NZF constructs were cloned into pOPIN-K, which encodes a 3C cleavable N-terminal His6-GST tag (Berrow et al., 2007) using Infusion HD cloning (Clontech). SMAC (56-239) and HtrA2 (134-458 and 359-458) were amplified with primers that encoded a C-terminal His6-tag and were ligated into an NcoI and HindIII digested pOPIN-K vector. To create the UBE2D2-OTUB1 fusion construct (OTUB1*), overlap extension PCR was used to amplify the full-length (1-147) human UBE2D2 sequence onto the 5' end of human OTUB1 (residues 16-271) with a 10-residue repeating 'Gly-Gly-Ser-Ser' linker sequence. This gene fusion was then inserted into the pOPIN-B vector to create a construct encoding an N-terminal 3C protease-cleavable His6-tag. A similar approach was used to create the STAM2-AMSH fusion (AMSH*), which encodes the VHS and UIM domains of mouse STAM2 (residues 5-188), followed by a 6-residue 'Gly-Gly-Ser-Ser-Gly-Gly' linker and the catalytic domain of human AMSH (residues 243-424). For TRABID localization experiments, TRABID (2-708) was cloned into pEGFP-C1 using XhoI and EcoRI restriction sites to create a TRABID construct with a C-terminal GFP fusion. Mutations were generated by site-directed mutagenesis using the QuickChange method with KOD HotStart DNA polymerase (Novagen). All constructs have been verified by DNA sequencing.

Protein expression and purification

His6-SUMO-AREL1, His6-SUMO-UBE3C, SMAC-His6, HtrA2-His6 and His6-GST-TRABID NZF constructs were expressed in Rosetta2 (DE3) pLacI cells. Cells were grown in 2xTY medium supplemented with 30 μg/ml kanamycin and 35 μg/ml chloramphenicol. The cultures were cooled to 18 °C prior to overnight induction with 400 μM IPTG. For TRABID NZF1, 200 μM of $ZnCl_2$

was added to the culture prior to induction. All proteins were purified by immobilized metal affinity chromatography using either a HisTrap column (GE Life Sciences) or TALON resin (Clontech). AREL1, UBE3C, SMAC-His6 and HtrA2-His6 cell pellets were resuspended in binding buffer (20 mM Tris pH 8.5, 300 mM NaCl, 50 mM imidazole, 2 mM β-mercaptoethanol) and TRABID NZF1 cell pellets were resuspended in binding buffer (50 mM Tris pH 7.4, 150 mM NaCl, 2 mM β-mercaptoethanol). Both cell suspensions were supplemented with lysozyme, DNaseI (Sigma) and protease inhibitor cocktail (Roche) and lysed by sonication. TRABID NZF1 was eluted from Talon resin and further purified using Glutathione Sepharose 4B resin (GE Life Sciences) before the His6-GST tag was cleaved by overnight incubation with 3C protease. Eluted TRABID NZF1 was further purified by size exclusion chromatography (HiLoad 16/60 Superdex 75, GE Life Sciences) in buffer containing: 20 mM Tris pH 7.4, 150 mM NaCl, 2 mM DTT. The resultant fractions were judged to be of sufficient purity following SDS-PAGE analysis and flash frozen. The His6-SUMO tag was cleaved from AREL1 by overnight incubation with SUMO protease at 4 °C. AREL1 was further purified by anion exchange chromatography (ResourceQ, GE Life Sciences) and size exclusion chromatography (HiLoad 16/60 Superdex 75, GE Life Sciences) in buffer containing: 20 mM Tris pH 8.5, 150 mM NaCl, 4 mM DTT. Fractions containing AREL1 were pooled and concentrated to 6 mg/ml and flash frozen. His6-SUMO-UBE3C, SMAC-His6 and HtrA2-His6 variants were eluted from the HisTrap column (GE Life Sciences) and subjected to size exclusion chromatography (HiLoad 16/60 Superdex 75, GE Life Sciences) in the same buffer used for AREL1. Peak fractions were pooled and concentrated prior to being flash frozen. TRABID AnkOTU (245-697) was expressed in ArcticExpress cells (Novagen) and purified according to (Licchesi et al., 2012). OTUB1* and AMSH* were expressed and purified as above, using Talon resin for initial affinity purification, followed by 3C cleavage and final purification using size exclusion chromatography

Ub chain assembly reactions using K-only Ub mutants

Small-scale analysis of HECT E3 ligases with Ub K-only mutants were carried out in 20 µl reactions containing 0.1 µM E1, 2 µM UBE2L3, 11 µM AREL1 or 4

μM GST-NEDD4L (576-955), 50 μM of indicated Ub mutants and 10 mM ATP in ligation buffer (10 mM $MgCl_2$, 40 mM Tris pH 8.5, 100 mM NaCl, 0.6 mM DTT and 10 % (v/v) glycerol). Reactions were incubated at 37 ºC for 60 min prior to being stopped with 4x LDS sample buffer (Invitrogen) and analyzed by SDS-PAGE, using 4-12% NuPAGE gradient gels (Invitrogen). Gels were stained using InstantBlue (Expedeon).

Substrate assembly reactions with AREL1

To investigate whether AREL1 is able to ubiquitinate inhibitors of apoptosis proteins, SMAC and HtrA2 , 150 μl assembly reactions were prepared containing: 0.1 μM E1, 2 μM UBE2L3, 11 μM AREL1, 400 μM Ub in ligation buffer (10 mM $MgCl_2$, 40 mM Tris pH 8.5, 100 mM NaCl, 0.6 mM DTT and 10 % (v/v) glycerol). Reactions contained 50 μM of substrate (SMAC-His6 (52-239), HtrA2-His6 (134-458 and 359-458) and were incubated for 60 min at 37 ºC with/without 10 mM ATP. Reactions were diluted with 500 μl with HisTrap binding buffer (20 mM Tris pH 8.5, 300 mM NaCl, 50 mM imidazole, 2 mM β-mercaptoethanol) and bound to 15 μl Ni-NTA resin (Qiagen). Unbound E2, E3 and Ub were removed through subsequent washes with HisTrap binding buffer before the bound protein was eluted in 2 x 30 μl washes with elution buffer (20 mM Tris pH 8.5, 300 mM NaCl, 500 mM imidazole, 2 mM β-mercaptoethanol). Reactions were analyzed by SDS-PAGE, using 4-12% NuPAGE gradient gels (Invitrogen) and stained using InstantBlue (Expedeon).

K29 chain generation

Large-scale assembly reactions of K29-linked polyUb chains were performed overnight at 37 °C in a reaction volume of 1 ml consisting of 3 mM Ub, 1 μM E1, 10 μM UBE2L3, 32 μM His6-SUMO UBE3C (693-1083), 10 mM ATP, 10 mM $MgCl_2$, 40 mM Tris pH 8.5, 100 mM NaCl, 0.6 mM DTT and 10 % (v/v) glycerol. Following acid precipitation of the reaction enzymes through the addition of perchloric acid (0.25 % final amount), the unanchored Ub chains were buffer exchanged into 50 mM Tris pH 7.4, 150 mM NaCl, 4 mM DTT using a NAP10 desalting column (GE Life Sciences). Linkage-specific deubiquitinating enzymes, OTUB1* (K48-specific) and AMSH* (K63-specific) were added to a final concentration of 1 μM and Cezanne (K11-specific) was

added at 400 nM and incubated for 60 min at 37 °C. Further acid precipitation (0.25 % perchloric acid) removed the DUBs before the sample was buffer exchanged into cation exchange buffer (50 mM NaOAc pH 4.5, 5 % (v/v) glycerol) using a PD10 desalting column (GE Life Sciences). K29-linked Ub chains were resolved by cation exchange chromatography using a MonoS column (GE Life Sciences) and eluted with elution buffer (50 mM NaOAc pH 4.5, 1M NaCl, 5 % (v/v) glycerol). The inclusion of glycerol was important to prevent monoUb inclusion within peak fractions. Peak fractions were dialyzed overnight against 50 mM Tris pH 7.4 prior to concentration (Amicon spin concentrators, 3 kDa molecular weight cut-off).

K33 chain generation

Large-scale assemblies of K33-linked polyUb chains were performed in an analogous way to K29-linked chains. 36 µM AREL1 was used in an assembly reaction in buffer described for K29-chain assembly. The addition of 5 % (v/v) glycerol in the reaction buffer was important to prevent AREL1 precipitation during the reaction. Acid precipitation followed by linkage-specific DUB addition, removed other Ub linkages. Subsequent acid precipitation of the DUBs and cation exchange chromatography allowed the purification of K33-linked chains. Peak fractions were dialyzed overnight against 50 mM Tris pH 7.4 prior to concentration (Amicon spin concentrators, 3 kDa molecular weight cut-off).

Ub chain composition mass spectrometry analysis

Ub chains were separated on a NuPAGE 4-12% gradient gel (Invitrogen) before in-gel digestion with trypsin and the addition of Ub AQUA peptide internal standards according to (Kirkpatrick et al., 2006). Peptides were extracted from gel slices, lyophilized and stored at -80 °C. Tryptic peptides were resuspended in 30 µl of reconstitution buffer (7.5% ACN, 0.5% TFA, 0.01% H_2O_2). Oxidation of peptides containing methionine residues was performed according to (Phu et al., 2010). Two additional 2-fold dilutions for each sample were prepared in order to assess the linearity of detection. 10 µl of each sample was directly injected onto an EASY-Spray reverse-phase column (C18, 3 µm, 100 Å, 75 µm x 15 cm) using a Dionex UltiMate 3000

HPLC system (Thermo Fisher Scientific). Peptides were eluted using a 25 min ACN gradient (2.5-35%) at a flow rate of 1.4 μl min^{-1}. Peptides were analyzed on a Q-Exactive mass spectrometer (Thermo Fisher Scientific) using parallel reaction monitoring (PRM), similar to (Tsuchiya et al., 2013). For PRM assays, monoisotopic precursor masses were isolated (2 *m/z* window) and fragmented at predetermined chromatographic retention times. Precursor masses were fragmented using the following settings: resolution, 17,500; AGC target, 1E5; maximum injection time, 120 ms; normalized collision energy, 28. Raw files were searched and fragment ions quantified using Skyline version 2.5.0.6157© (MacLean et al., 2010). Data generated from Skyline was exported into a Microsoft Excel spread sheet for further analysis according to (Kirkpatrick et al., 2006).

Pull-down assays

Pull-down assays were generally performed as previously (Kulathu et al., 2009). Briefly, 30 μg of GST-tagged NZF domain constructs were incubated with 25 μl of Glutathione Sepharose 4B (GE Life Sciences) for 1 h at 4 °C in 450 μl of pull-down buffer (PDB; 50 mM Tris pH 7.4, 150 mM NaCl, 2 mM β-mercaptoethanol, 0.1 % NP-40). The beads were washed 3 x with PDB and then incubated with 1.5 μg of the indicated diUb in 450 μl PDB plus 0.2 mg/ml BSA overnight at 4 °C. The beads were then washed 5x with PDB prior to SDS-PAGE. Proteins were visualized by silver staining using the Silver Stain Plus Kit (BioRad) according to manufacturers protocols or by Western blotting using a rabbit anti-Ub antibody (Millipore).

Surface plasmon resonance

SPR experiments were performed using a Biacore 2000 with CM5 chips (GE Healthcare), which were functionalized with a 1:1 mix of EDC/NHS. The differently linked diUbs were diluted in 20 mM sodium acetate buffer pH 5.0 to a concentration of 100 ng/μl and centrifuged at 16,000*g* for 10 min to remove aggregates. The diluted protein sample was injected until a response of ~2000 RU was reached. The chip was then blocked using 1 M ethanolamine pH 8.0. One flow channel per chip was functionalized and blocked without the addition of protein to serve as a reference. For affinity measurements, the

samples were buffer exchanged into SPR buffer (20 mM Tris pH 7.4, 150 mM NaCl, 2 mM DTT), and injected for 60 s followed by 150 s dissociation in SPR buffer at 20 °C. The K_d was determined from the data of two experiments using the reference-corrected equilibrium response with the data fitted in Prism 6 (GraphPad Software). Data was fit to a one-site binding model with reasonable absolute sum of squares (ranging from 24-501), except for K29 diUb:NZF1, K29 diUb:NZF1, K33 diUb:NZF1 and K33 diUb:NZF1 Y15F where a two-site binding model was more appropriate. The absolute sum of squares for the four fits dropped from 11938 to 176.5, from 3431 to 338.9, from 22095 to 151.5 and from 8638 to 506.1, respectively, when switching from a one-site to a two-site binding model.

Protein production for NMR analysis

For NMR experiments, cells were grown in 2M9 medium supplemented with ^{15}N-NH_4Cl and/or ^{13}C-glucose. Labeled Ub was purified and assembled into chains as described above. Prior to NMR analysis, all proteins were buffer exchanged against NMR phosphate buffered saline (NMR PBS, 18 mM Na_2HPO_4, 7 mM NaH_2PO_4 pH 7.2, 150 mM NaCl) and 5% D_2O was added as a lock solvent.

NMR analysis

NMR acquisition was carried out at 298 K on a Bruker Avance III 600 MHz spectrometer equipped with a cryogenic triple resonance TCI probe. Topspin (Bruker) and Sparky (Goddard & Kneller, UCSF; http://www.cgl.ucsf.edu/home/sparky/) software packages were used for data processing and analysis, respectively. ^{1}H,^{15}N 2D BEST-TROSY experiments (Favier and Brutscher, 2011) were acquired with in-house optimized Bruker pulse sequences incorporating a recycling delay of 400 ms and 512*64 complex points in the ^{1}H,^{15}N dimension, respectively. High quality 2D data sets were acquired in ~10 min. Weighted chemical shift perturbation calculations were completed using the equation $\sqrt{(\Delta^{1}H)^2+((\Delta^{15}N)^2/5)}$.

To determine the K_d for each NZF, increasing concentrations of the ligand (20, 50, 100, 200, 300, 400, 600, 850 μM) were titrated into a constant concentration of 80 μM wt ^{15}N-Ub. Changes in either the ^{1}H or ^{15}N chemical

shift of residues were plotted against the NZF ligand concentration and data fitted using the equation $\Delta\delta_{obs} = \Delta\delta_{max} \{([P]_t+[L]_t+K_d)-[([P]_t+[L]_t+K_d)^2-4[P]_t[L]_t]^{1/2}\}/2[P]_t$ where $\Delta\delta_{obs}$ is the change in chemical shift from the unbound state, $\Delta\delta_{max}$ is the maximum chemical shift change that occurs at saturation of the Ub and $[P]_t$ and $[L]_t$ are the total protein concentrations of Ub and NZF in each sample (Williamson, 2013).

The backbone assignment of the wt K33 diUb BEST-TROSY was confirmed using an HNCA triple resonance experiment, collected with 1024*32*64 complex points in the ^{1}H, ^{15}N and ^{13}C dimensions respectively. 16 of the 17 additional cross peaks seen in the diUb in comparison to wt Ub were assigned to the proximal molecule with one additional cross peak identified as the amide formed by the isopeptide linkage between the distal Gly76 and the proximal K33 side chain.

NZF1 was added to both the wt K33 diUb sample and the wt K29 diUb sample at a 1:1 concentration ratio. The assignment of the BEST-TROSY spectrum of the K33 diUb with ZNF1 was again confirmed with a triple resonance HNCA experiment collected as above, supplemented with a CBCACONH spectrum collected with 50% Non Uniform Sampling (NUS) and 1024*32*48 complex points in the ^{1}H, ^{15}N and ^{13}C dimensions respectively. The NUS spectrum was processed using Compressed sensing with the MddNMR software package {Kazimierczuk:2011jy}.

Crystallization, data collection and refinement

Crystals of K33-linked diUb were grown by sitting-drop vapor diffusion by mixing an equal volume of K33 diUb (K11R) at 5.5 mg/ml with reservoir (100 mM Tris pH 8.0, 3.0 M $(NH_4)_2SO_4$). Crystals were transferred to a solution containing 3.2 M $(NH_4)_2SO_4$ prior to cryo-cooling. Crystals of K33 triUb were obtained by mixing an equal volume of K33 triUb (K11R) at 3.2 mg/ml with reservoir (10% PEG 20K, 20% PEG 550 MME, 0.02 M D-glucose, 0.02 M D-mannose, 0.02 M D-galactose, 0.02 M L-fructose, 0.02 M D-xylose, 0.02 M N-acetyl-D-glucosamine, 100 mM Tris/Bicine pH 8.5). Crystals grew after 14 days and did not require cryo-protecting prior to vitrification. For crystallization of the TRABID NZF1:K33-linked diUb complex, K33 diUb (assembled from K11R) was mixed with a 1.2 molar excess of TRABID NZF1 (1-33) to a final

complex concentration of 6 mg/ml. Crystals grew at 4 °C from a 1:2 (v/v) ratio of protein to reservoir solution containing 20% PEG 550 MME, 10% PEG 20K, 0.1 M MES/imidazole (pH 6.5), 0.12 M Na oxamate, 0.12 M NaF, 0.12 M Na citrate and 0.12 M Na/K-tartrate (racemic). Crystals appeared after two days and did not require cryo-protecting prior to vitrification.

Diffraction data were collected at Diamond Light Source beam lines I03 for K33-linked diUb and I24 for K33-linked triUb and K33-linked diUb:TRABID NZF1. Diffraction images were integrated using XDS (Kabsch, 2010) and scaled using AIMLESS (Evans and Murshudov, 2013). The structures of K33 diUb and triUb were solved by molecular replacement using Ub (pdb-id 1ubq, {VijayKumar:1987wy}) as an initial search model in PHASER (McCoy et al., 2007). Eight Ub monomers could be placed for the K33 diUb model. However, owing to the size of the asymmetric unit only one Ub monomer could be placed for the K33 triUb model. Iterative rounds of model building and refinement were performed using PHENIX (Adams et al., 2011) and COOT (Emsley et al., 2010), respectively, with TLS restraints used in the latter rounds of refinement. For the K33 diUb model, electron density was of sufficient quality to unambiguously build the isopeptide linkage for three out of the four K33-linked diUbs. However, for the K33 triUb model owing to the isopeptide bond extending across the asymmetric unit (resulting in 2/3 occupancy) and also flexibility of the C-terminal arginyl-glycyl-glycine sequence, then the electron density was not of sufficient quality to confidently build one/two conformations of the Ub C-terminus. The weighted |Fo|-|Fc| had difference density around the ε-amino group of K33, indicating low occupancy isopeptide linkage and no linkage was modeled. TRABID NZF1 K33-linked diUb structure was also solved by molecular replacement using Ub (pdb-id 1ubq) and the TAB2 NZF domain (pdb-id 2wwz, {Kulathu:2009dd}) as search models and refined similarly as the K33 diUb structure. Crystal averaging coupled with flexibility of the C-terminus resulted in weak electron density for the isopeptide bond.

Final statistics can be found in **Table 1**. All structural figures were generated using Pymol (www.pymol.org).

Analysis of TRABID C443S localization

COS-7 cells were grown on coverslips to a confluence of approximately 70%. 24 h after transfection of GFP-ciTRABID constructs (250ng), cells were fixed with 4% paraformaldehyde and permeabilized with 0.1% (v/v) Triton X-100 in PBS and mounted using Vectashield mounting medium with DAPI (Vector Laboratories). Confocal images were taken using the Zeiss LSM 780 microscope. Images of at least 40 cells were analyzed using the Nikon NIS Elements software (Nikon Instruments). The data was plotted in Prism 6 (GraphPad Software) and because the data was not normally distributed, a Mann-Whitney test was performed to determine statistical significance.
